# Kinome profiling reveals breast cancer heterogeneity and identifies targeted therapeutic opportunities for triple negative breast cancer

**DOI:** 10.18632/oncotarget.1865

**Published:** 2014-03-26

**Authors:** Fares Al-Ejeh, Mariska Miranda, Wei Shi, Peter T. Simpson, Sarah Song, Ana Cristina Vargas, Jodi M. Saunus, Chanel E. Smart, Mythily Mariasegaram, Adrian P. Wiegmans, Georgia Chenevix-Trench, Sunil R. Lakhani, Kum Kum Khanna

**Affiliations:** ^1^ Signal Transduction Laboratory, QIMR Berghofer Medical Research Institute, Herston QLD 4006, Australia; ^2^ The University of Queensland, St Lucia QLD 4072, Australia; ^3^ The University of Queensland, UQ Centre for Clinical Research, Herston QLD 4029, Australia; ^4^ The University of Queensland, QLD Centre for Medical Genomics, St Lucia QLD 4072, Australia; ^5^ Cancer Genetics Laboratory, QIMR Berghofer Medical Research Institute, Herston QLD 4006, Australia; ^6^ The University of Queensland, School of Medicine, Herston QLD 4006, Australia; ^7^ Pathology Queensland: The Royal Brisbane & Women's Hospital, Herston QLD 4029, Australia

**Keywords:** Breast cancer, TNBC, kinome, cancer heterogeneity, targeted therapy

## Abstract

Our understanding of breast cancer heterogeneity at the protein level is limited despite proteins being the ultimate effectors of cellular functions. We investigated the heterogeneity of breast cancer (41 primary tumors and 15 breast cancer cell lines) at the protein and phosphoprotein levels to identify activated oncogenic pathways and developing targeted therapeutic strategies. Heterogeneity was observed not only across histological subtypes, but also within subtypes. Tumors of the Triple negative breast cancer (TNBC) subtype distributed across four different clusters where one cluster (cluster ii) showed high deregulation of many proteins and phosphoproteins. The majority of TNBC cell lines, particularly mesenchymal lines, resembled the cluster ii TNBC tumors. Indeed, TNBC cell lines were more sensitive than non-TNBC cell lines when treated with targeted inhibitors selected based on upregulated pathways in cluster ii. In line with the enrichment of the upregulated pathways with onco-clients of Hsp90, we found synergy in combining Hsp90 inhibitors with several kinase inhibitors, particularly Erk5 inhibitors. The combination of Erk5 and Hsp90 inhibitors was effective *in vitro* and *in vivo* against TNBC leading to upregulation of pro-apoptotic effectors. Our studies contribute to proteomic profiling and improve our understanding of TNBC heterogeneity to provide therapeutic opportunities for this disease.

## INTRODUCTION

Gene expression profiling has contributed significantly to our understanding of breast cancer heterogeneity, biology and prognosis. Patterns of gene expression dissect the heterogeneity of breast cancer into five subtypes (luminal A, luminal B, basal-like, HER-2 and normal-like) that relate to histopathological parameters and immunophenotyping [1, 2]. More recently, integrative mutational and transcriptome profiling divides breast cancer to ten subgroups with distinct clinical outcome [3]. In contrast, our understanding of breast cancer heterogeneity at the proteins level is limited despite proteins being the ultimate effectors of cellular functions.

Reverse phase protein arrays (RPPA) are widely used multiplexed arrays to study selected proteins in clinical specimens. RPPA in microdissected breast tumors recognized the classification of breast cancer to subgroups according to protein and phosphoprotein level [4]. The robustness of RPPA in non-microdissected breast tumors has been illustrated from technical perspectives; variability in tissue handling and intratumoral vs. intertumoral heterogeneity [5]. Importantly, Hennessy *et al* [5] demonstrated that RPPA can classify breast tumors to the same subtypes deduced from transcriptome profiling. Moreover, this study supported the use of RPPA in non-microdissected breast tumors in the comprehensive cancer genome atlas (TCGA) study in breast cancer which also found proteome-based breast cancer subtypes which are highly concordant to transcriptome subtypes [6]. RPPA-based proteomics have also succeeded in the identification of proteins and phosphoproteins which associate with the prognosis of breast cancer [7-9]. Unlike RPPA which is limited to 100 – 200 analytes, mass spectroscopy (MS)-based proteomics can interrogate several hundreds of proteins. Indeed several studies used MS-based proteomics to identify biomarkers and targets for particular subtypes or disease progression and metastasis in breast cancer [10-12]. However, due to the complex nature of this approach, limited studies use MS to investigate the heterogeneity of breast cancer [13, 14]. These two studies, using cell lines, again revealed that the proteome fingerprint classify breast cancer to subtypes similar to transcriptome classification.

Notably, although RPPA- and MS-based proteomic studies reveal concordance with transcriptome-based subtypes, these studies observed low correlation between protein and mRNA levels of their protein classifiers [5, 13, 14]. This suggests that although the proteome fingerprint retains a similar classification of breast cancer to the transcriptome fingerprint, the proteome fingerprint is not identical and may not be predicted from mRNA levels. The lack of strong correlation between mRNA abundance and protein expression is not surprising since this relationship is not direct [15, 16], thus supporting the need for protein profiling.

In this article, we describe the profiling of protein levels and phosphorylation levels in aggressive/high grade primary breast tumors and established cell lines using the Kinex™ antibody microarrays. The Kinex™ antibody microarrays are as simple as RPPA technically but interrogate more than 400 kinases and kinase-associated proteins using validated antibodies [reviewed in 17]. The Kinex™ antibody arrays have been used in several studies to compare cancer cell lines ([e.g. 18]). We focused on triple negative breast cancer (TNBC); a subtype associated with poor prognosis, and found that a subgroup in TNBC showed the highest and complex deregulation of proteins and phosphoproteins in comparison to hormone-positive tumors. We found that breast cancer cell lines recapitulate the patterns observed in the primary tumors. In our effort to functionally translate our finding, we identified TNBC cell lines to be sensitive to targeted inhibitors of several of the activated kinases we identified in patient samples. Finally, based on our findings, we rationalized the combination of Hsp90 and Erk5 inhibition as a therapeutic strategy against TNBC and demonstrated the efficacy of this combination *in vitro* and *in vivo*.

## RESULTS

### Profiling of primary breast cancer tumors and cell lines with the Kinex™ antibody arrays

**Figure 1:**
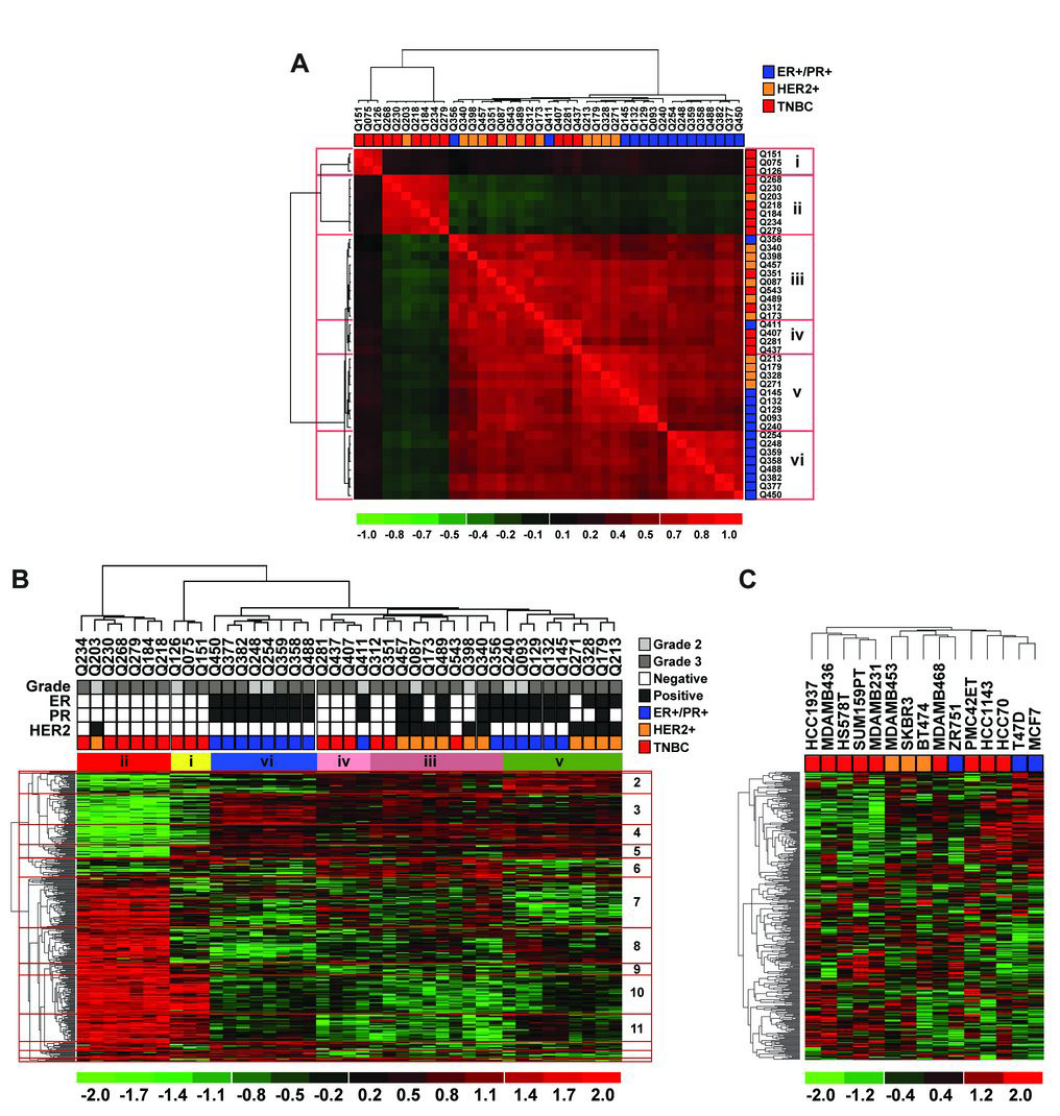
The Kinex antibody arrays confirm the heterogeneity of breast cancer Lysates from fresh frozen primary, high grade, breast tumors prior to therapy were subjected to the Kinex™ antibody arrays (Supplementary Fig. S1). (A) Sample-sample correlation plot using all the probes revealed six clusters (i – vi) which were compared by the SAM method and 1.75-fold change cutoff (Supplementary Fig. S1). (B) Hierarchal clustering of patient tumors based on the differentially expressed proteins/phosphoproteins. The numbers on the right of the heat map mark the 14 branches which differentiate the tumor clusters and these are annotated in Supplementary Table 2S. (C) Lysates from established breast cancer cell lines were also subjected to the Kinex™ arrays. Hierarchal clustering of the breast cancer cell lines was supervised by the probes that were differentially expressed across the six clusters of primary patient tumors

The Kinex™ KAM-1.3 antibody microarrays contain 812 probes (in duplicates; i.e. > 1624 spots) which investigate more than 400 kinases and kinase-associated proteins. We extracted protein from 41 fresh frozen primary breast tumors; 15 triple negative (TNBC), 15 hormone positive (ER/PR-positive/HER2-negative) and 11 HER2-positive tumors. These histological subgroups were similar in terms of median patient age, tumor size, histological grade and tumor cell content (Supplementary Table S1). Moreover, the correlation between the replicates of probes was higher than 0.85 for each tumor (Pearson's correlation coefficient, Supplementary Table S1); illustrating the consistency of these arrays. Using all the probes in the Kinex™ arrays and unsupervised hierarchal clustering, the primary tumors separated to several clusters (Supplementary Fig. S1A), illustrating the heterogeneity of breast cancer at the protein and phosphoprotein levels. At least six clusters were defined by the sample-sample correlation plot shown in Fig.1A (clusters i – vi). Significance Analysis of Microarray (SAM) with a 1.75-fold cutoff (Supplementary Fig. S1B) identified 349 differentially expressed proteins and phosphoproteins between these six clusters (Fig.1B and Supplementary Table S2). TNBC tumors were heterogeneous where six TNBC tumors were in cluster ii along with one HER2-positive tumor and three TNBC tumors were alone in cluster i. Cluster iii contained three TNBC tumors with six HER2-positive tumors and one ER/PR-positive tumor and the remaining three TNBC tumors clustered with one ER/PR-positive tumors in cluster iv. Clusters v consisted of five ER/PR-positive and four HER2-positive tumors and cluster vi of eight ER/PR-positive tumors. We also profiled 15 breast cancer cell lines using the Kinex™ KAM-1.3 antibody microarrays and found that the 349 differentially expressed probes from the patient tumors generated three clusters in the breast cancer cell lines (Fig.1C). One cluster contained five TNBC cell lines while the remaining four TNBC cell lines clustered with HER2-positive or ER-positive cell lines. Of note, most of TNBC cell lines we tested, particularly the mesenchymal breast cancer cell lines (MDA-MB-436, MDA-MB-231, Hs578T and SUM159PT), resembled cluster ii in patients. We focused on cluster ii because it showed the highest deregulation of many probes compared to the other clusters and contained the largest number of TNBC primary tumors and TNBC cell lines.

### Concordance of the Kinex™ array results and the TCGA RPPA data

We analyzed the RPPA data (level 3 data) from the breast cancer TCGA study data [6] in the same way we analyzed our Kinex™ antibody arrays. In agreement with our results, at least six clusters were defined by the sample-sample correlation plot (Supplementary Fig. S2) using all the probes in the TCGA RPPA arrays (166 probes). TNBC tumors were heterogeneous where 16/58 (28%) of TNBC tumors appeared in one cluster, 32/58 (55%) of TNBC tumors appeared in a second cluster and the remaining 10/58 (17%) of TNBC tumors divided equally into two additional clusters (Supplementary Fig. S2). We compared the two main TNBC clusters to all other tumor clusters in the TCGA RPPA data and found that 65 probes that detect 61 proteins and phosphoproteins were deregulated in these tumors (1.5-fold cutoff, Supplementary Table S3). Of these 61 proteins and phosphoproteins, 26 did not have probes to detect them in the Kinex™ antibody arrays; therefore we were able to compare the concordance between our results and the TCGA RPPA data based on 35 proteins/phosphoproteins. Of these 35 proteins/phosphoproteins in the TCGA RPPA, 24 (69%) were also deregulated in the TNBC clusters in our Kinex™ arrays in the same direction (marked in Supplementary Table S3). The remaining 11 proteins/phosphoproteins in the TCGA RPPA results did not meet the threshold criteria in our study (7 proteins/phosphoproteins) or showed deregulation in the opposite direction (4 proteins/phosphoproteins). Thus, the heterogeneity and the clustering of breast cancer in general, and in TNBC specifically, could be replicated in the TCGA RPPA data which profiled more tumors. The advantage of the Kinex™ antibody arrays however, resides in the larger coverage of proteins/phosphoproteins (812 probes in the Kinex™ arrays vs. 166 probes in the TCGA RPPA). Altogether, there is a high agreement between our results and those from TCGA RPPA data.

### Validation of deregulated proteins and phosphoproteins in TNBC cluster ii

**Figure 2:**
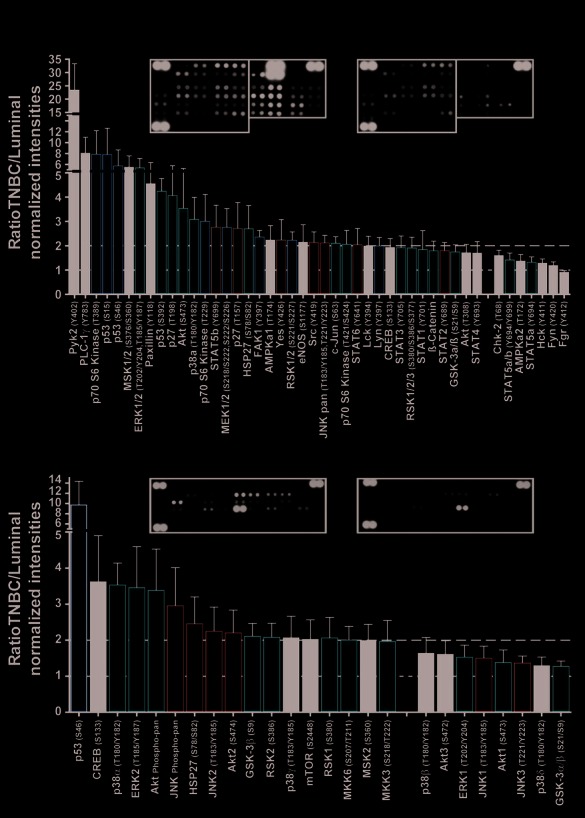
Validation of phosphorylations of proteins identified in the Kinex™ antibody arrays using the Proteome Profiler antibody arrays Lysates from four TNBC cell lines (MDA-MB-231, Hs.578T, BT549 and MDA-MB-435) and four luminal cell lines (MCF7, T47D, MDA-MB-175 and ZR751) were analyzed using the Proteome Profiler™ Array for (A) human phospho-kinases and (B) human phospho-MAPK pathway. Bar graphs show the average ratio of TNBC/luminal normalized intensity for each phosphoprotein (±SEM, n = 4 cell lines each with two technical replicate for each phosphoprotein). Dashed lines mark the 1-fold (no change) and the 2-fold ratios and insets show representative arrays. Red bars: probes which validate exact phosphorylations in the Kinex™ arrays. Orange bars: probes that identify additional phosphorylations of phosphoproteins to those identified in the Kinex™ arrays. Blue bars: probes identifying phosphoproteins that were upregulated at the protein level in the Kinex™ arrays. Black bars: probes that were not present in the Kinex™ arrays or did not meet the cutoff criteria in analysis

To validate the deregulated phosphoproteins discovered in cluster ii in the Kinex™ arrays, we used independent small arrays, the Proteome Profiler™ Arrays for human phospho-kinases and phospho-MAPK pathway (R&D Systems). We compared the phosphorylation levels in four TNBC cell lines (MDA-MB-231, Hs.578T, BT549 and MDA-MB-435) which resemble cluster ii in patients and four luminal cell lines (MCF7, T47D, MDA-MB-175 and ZR751) which resemble cluster vi (pure ER/PR-positive) patients. As shown in Fig.2 and Supplementary Table S2, the R&D arrays validated the upregulation of 18 phosphoantibodies in cluster ii (marked in red). Furthermore, the R&D arrays identified additional phosphorylation sites of 15 upregulated phosphoproteins in cluster ii in the Kinex™ arrays (marked in orange in Fig.2 and Supplementary Table S2). Moreover, 13 upregulated phosphoantibodies in the R&D arrays could be explained by the upregulation of the total protein in the Kinex™ arrays (marked in blue in Fig.2 and Supplementary Table S2). Finally, the R&D arrays detected the upregulation of phosphoproteins (marked in black in Fig.2) which were not present in the differentially expressed phosphoproteins in the Kinex™ arrays. Some of these phosphoantibodies were not present in the Kinex™ arrays while the remaining did not meet the cutoff criteria in the analysis. In summary, of the 59 upregulated (> 1.5-fold) phosphorylations in cluster ii *vs*. cluster vi in the Kinex™ arrays, the R&D arrays contained antibodies against 18 phosphorylation sites and all validated.

**Figure 3:**
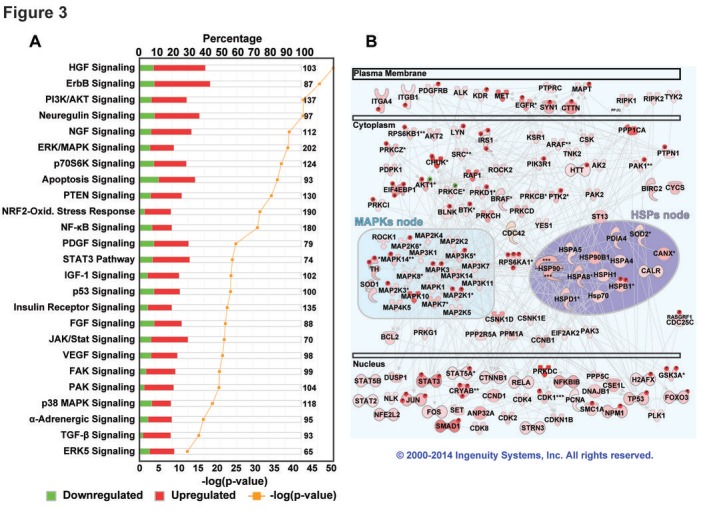
Pathway analysis of deregulated proteins and phosphoproteins in TNBC Pathway analysis in IPA® using only direct protein-protein interaction data was used and the deregulated proteins/phosphoproteins in TNBC cluster ii identified three overlapping networks. These networks were merged and visualized (Supplementary Fig. S3). (A) Canonical signaling pathways using IPA® which were enriched (Fisher's exact test p-value) in the deregulated proteins/phosphoproteins in TNBC cluster ii subgroup. (B) The upregulated phosphoproteins (marked with P) and proteins in cluster ii were analyzed using IPA® and a single network of direct protein-protein interactions was identified. Shades of pink/red reflect the extent of upregulation. At least two nodes, MAPKs and HSPs, were clearly identified in the center of complex signaling cascades.

Next, we used the Ingenuity Pathway Analysis (IPA®) software to determine networks and pathways that describe the protein-protein interactions (PPI) relationships between the deregulated proteins and phosphoproteins in cluster ii tumors compared to cluster vi tumors. Three overlapping networks were enriched in this analysis (Supplementary Fig. S3A) and were merged to obtain one network (Supplementary Fig. S3B). The canonical signaling pathways that were activated by the deregulation of proteins/phosphoproteins in the cluster ii subgroup are shown in Fig.3A. By analyzing the network of direct protein-protein interactions for upregulated proteins and phosphoproteins only, robust and cross-talking signaling cascades from the plasma membrane to the nucleus were identified in cluster ii tumors (Fig.3B). At least two nodes, mitogen activated protein kinases (MAPKs) and heat shock proteins (HSPs) nodes, were at the center of these complex signaling cascades and pathways.

**Figure 4:**
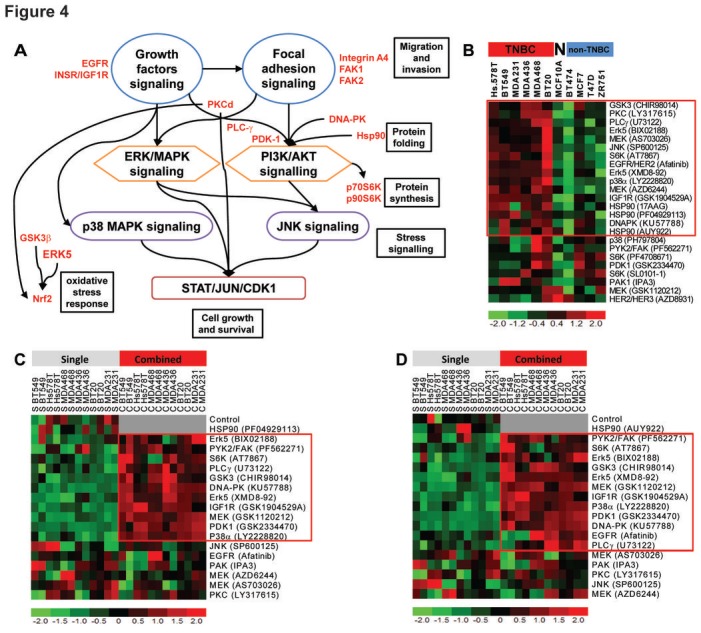
Targeted inhibition of activated kinases in cluster ii kills TNBC cell lines (A) Activated cross-talking canonical signaling pathways in TNBC tumors in cluster ii. (B) Six TNBC cell lines, four non-TNBC cell lines and the “near-normal” MCF10A cell line were treated (in duplicates) with escalating doses of the specified drugs and survival was measured six days after treatment using the CellTitre™ assay. Data shown is -log10[IC50], a measure of sensitivity, where red color denotes relative increase in sensitivity. The red box marks drugs which specifically killed TNBC cell lines in comparison to non-TNBC cell lines. The raw data of dose response curves are shown in Supplementary Fig. S4. (C & D) Two Hsp90 inhibitors (PF04929113 and AUY922) were separately used as single agents or in combination with the specified kinase inhibitors. The combinations were performed using the IC25 of each drug against the specified TNBC cell lines. Data shown is the survival of cells where red color represents more killing than green color. Raw data for panels C and D are shown in Supplementary Fig. S5.

### Proteomic profiles may not be accurately predicted by transcriptome profiling

The phosphorylation of proteins may not be predicted by changes in mRNA levels; however, the upregulation of proteins may be associated with increased abundance of mRNA transcripts. To this end, we interrogated the Oncomine™ database to determine whether the upregulated proteins in our screen were accompanied with elevated mRNA in TNBC. We analyzed two large patient datasets, the TCGA [6] and the METABRIC [3], and the Neve *et al.* breast cancer cell lines dataset [19], and found that only 9 of the 49 (18%) upregulated proteins we identified in TNBC had evidence for elevated mRNA levels (Supplementary Table S4). This poor protein-mRNA correlation was in agreement with previous proteomic-based profiling in breast cancer [5, 13, 14]. Upon closer examination, we found that many of the activated kinases in our screen are oncoclients of the heat shock protein Hsp90; known to stabilize these proteins [20-22]; thus the elevation of proteins in cluster ii may be due to stabilization rather than elevation of mRNA levels. In agreement, the upregulation of Hsp90 protein was consistently detected in TNBC tumors and cell lines using 10 independent probes on the Kinex™ arrays.

### *In vitro* survival and growth after inhibition of signaling networks

Based on the signaling cascades envisioned from the kinome profiling/pathway analysis above and outlined in Fig.4A, we compared the sensitivity of TNBC cell lines (MDA-MB-231, BT549, MDA-MB-436, Hs.578T, MDA-MB-468 and BT20), non-TNBC cell lines (MCF7, T47D, ZR751 and BT474) and the “near-normal” MCF10A cell line to 24 small molecule inhibitors. Cell survival at six days after treatment showed that TNBC breast cancer cell lines were more sensitive than non-TNBC cell lines to 13 drugs (Fig.4B and Supplementary Fig. S4). Given the high level of Hsp90 we identified and the role of this chaperon in oncogene activation, we investigated the combinations of the molecular inhibitors with Hsp90 inhibitors. Two different inhibitors of Hsp90 showed synergy in the killing of TNBC cell lines when combined with inhibitors targeting PAK1, FAK/PYK2, IGF-1R/IR, PKC, AKT/p70S6K, GSK3, p38 MAPK, JNK, DNA-PK or Erk5 (Fig. 4C&D and Supplementary Fig. S5). Particularly, the combination of Hsp90 inhibition with Erk5 inhibition produced the highest and most consistent synergy amongst the six TNBC cell lines tested (Supplementary Fig. S5).

### Erk5 as a therapeutic target in TNBC

**Figure 5:**
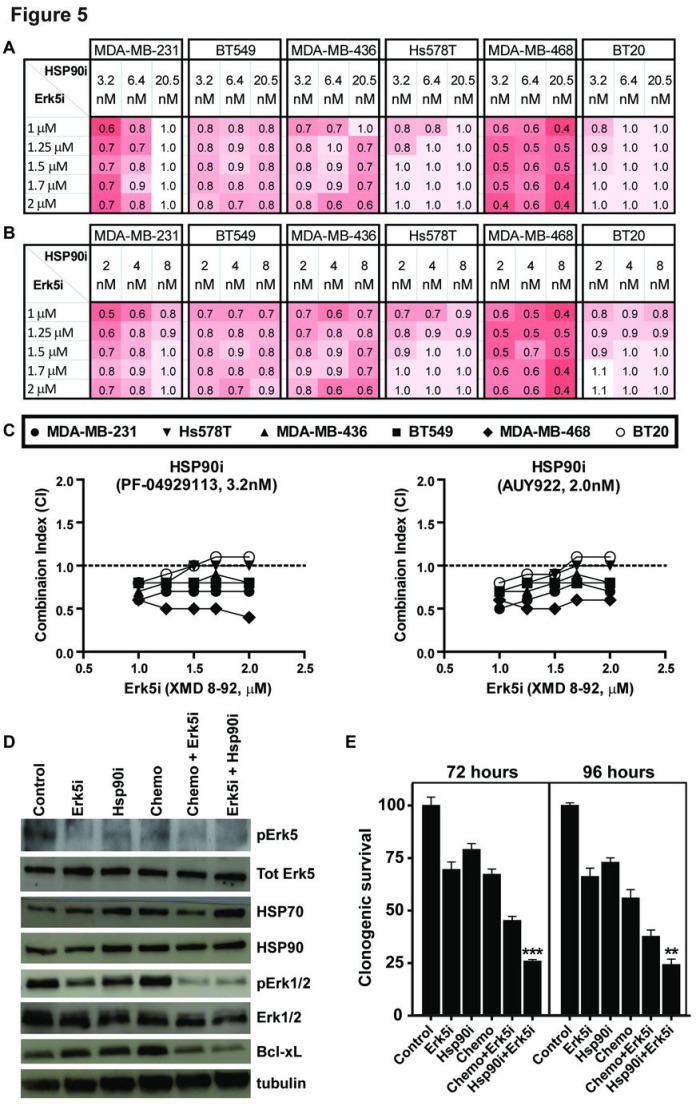
The combination of Hsp90 inhibition and Erk5 inhibition is synergistic in TNBC (A&B) Cultures of six TNBC cell lines were treated with escalating doses of ERK5i XMD 8-92 combined with Hsp90i PF-04929113 (A) or the Hsp90i AUY922 (B) to calculate the combination index (CI, < 1 is synergistic). (C) Graph of CI for the Hsp90 inhibitors at the lowest doses with Erk5 inhibition. (D) Immunoblot analysis of MDA-MB-231 cells treated with 1 µM Erk5i (XMD 8-92) alone, 2 nM of Hsp90i (AUY922) alone, chemotherapy alone (2.5 nM docetaxel and 10 nM doxorubicin) or the combinations at 24 hours after treatment with indicated antibodies. (E) Clonogenic survival of MDA-MB-231 cells when subjected to treatments as indicated in D at 72 hours or 96 hours.

We focused on the inhibition of Erk5 (MAPK7) since Erk5 is an attractive target for cancer therapy [23, 24]. We confirmed the higher level of Erk5 in TNBC cell lines compared to non-TNBC cell lines (Supplementary Fig. S6). Activated Erk5 is not a client of Hsp90 [25] and the ERK5/Nrf2-mediated oxidative stress response pathway is activated while the ERK/MAPK signaling pathway is inhibited in response to Hsp90 inhibition [22]. Hsp90 inhibition in TNBC was shown to inhibit the phosphorylation of Erk1/2 and decrease the anti-apoptotic Bcl-xL protein [26]. Collectively, these observations are consistent with the synergy we observed when combining Erk5 inhibition with Hsp90 inhibition (Supplementary Fig. S6). To expand on this synergy, we investigated the combination of Erk5 inhibition with Hsp90 inhibition in cell killing across various doses. Indeed, low doses of Hsp90 inhibitors (Hsp90i) with the Erk5 inhibitor XMD 8-92 (Erk5i) showed strong synergy (Fig. 5A-C). Using these low doses, the phosphorylation of Erk5 was inhibited by the Erk5i and Hsp70 stabilization (surrogate marker for Hsp90 inhibition) was detected with the Hsp90i, confirming the specificity of these inhibitors (Fig.5D). The combination of Erk5i and Hsp90i or Erk5i with chemotherapy resulted in significant decrease in the phosphorylation of Erk1/2 and decrease in the anti-apoptotic Bcl-xL protein (Fig.5D). This explained the significant decrease in clonogenic survival observed in these combinations (Fig.5E).

**Figure 6:**
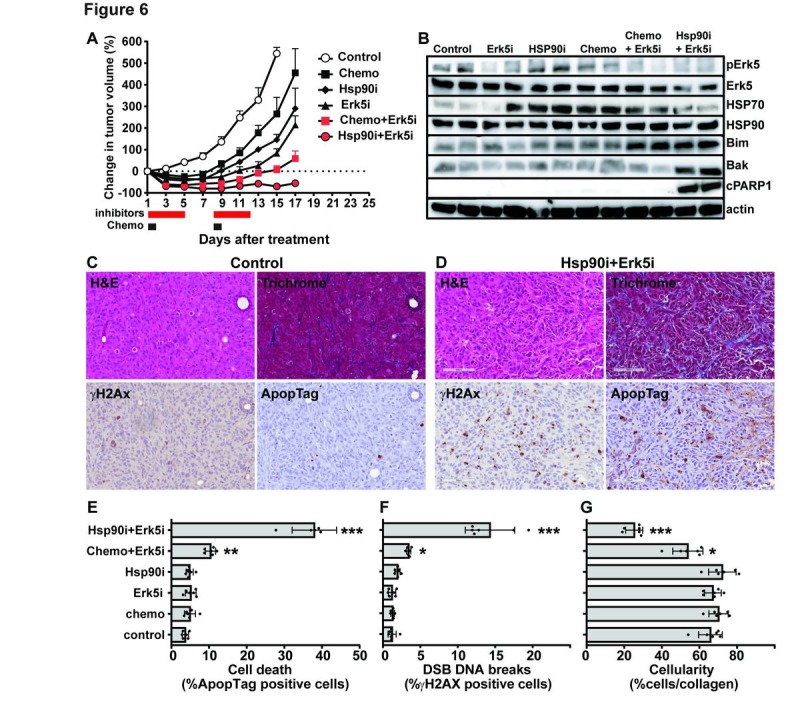
The combination of Erk5 and Hsp90 inhibitors *in vivo* Female nude mice bearing the TNBC MDA-MB-231 xenografts (50mm^3^ volume) were untreated (vehicle control) or treated with chemotherapy (chemo; 2 mg/kg docetaxel and 10 mg/kg doxorubicin administered i.p. on days 1 and 8). Additional groups of mice were treated with Erk5 inhibitor alone (Erk5i XMD 8-92 at 25 mg/kg) or Hsp90 inhibitor alone (Hsp90i AUY922 at 25 mg/kg) daily on days 1-5 and days 8-12. Additional groups of mice were treated with the combinations; chemo+Erk5i or Hsp90i+Erk5i. Six tumors were excised on day 13 (after treatment completion) for *ex vivo* studies. (A) Tumor growth curves based on change in tumor volume compared to day 0 prior to treatment (n=10 tumors/group). (B) Representative immunoblots from two tumors per group with indicated antibodies (cPARP1 – cleaved PARP1). Blots from additional four more tumors/group showed similar results (Supplementary Fig. S7). (D&E) Representative images (20× magnification, scale bar 100 µm) of tumor histology (H&E and trichrome staining) and IHC staining for DNA double strand breaks (DSBs, γH2AX staining) and apoptosis (ApopTag) from control and Hsp90i+Erk5i treated mice on day 13. Quantification of staining from six tumors/group was performed as in Methods; (E) ApopTag staining, (F) γH2AX staining, (G) cellularity: ratio of pink (cells) to blue (collagen) staining from trichrome staining. Error bars represent the standard error of the mean, * p<0.05, ** p<0.01 and *** p<0.001 from One-Way ANOVA (GraphPad® Prism).

Based on our *in vitro* results, we investigated these combinations with Erk5 inhibitor *in vivo* using the MDA-MB-231 TNBC xenografts model. Tumors treated with Erk5i alone (XMD 8-92 at 25 mg/kg) or Hsp90i alone (AUY922 at 25 mg/kg) showed a moderate delay in tumor growth (Fig.6A). We confirmed the inhibition of Erk5 phosphorylation by the Erk5i and the stabilization of Hsp70 by the Hsp90i by immunoblots on tumor lysates prepared on day 13 after the completion of treatments (Fig.6B and Supplementary Fig. S7). While the combination of Erk5i with chemotherapy (docetaxel+doxorubicin) reduced tumor growth, the combination of Erk5i and Hsp90i showed a strong anti-tumor response (Fig.6A). The latter combination was associated with prolonged apoptotic cell death as judged by the increase of pro-apoptotic Bcl family members Bim and Bak and the cleavage of PARP1 (Fig.6B and Supplementary Fig. S7) and ApopTag staining by IHC (Fig.6D&E). We also detected a significant increase in DNA double strand breaks (DSBs) detected by γH2AX staining (Fig.6F) and a significant reduction in tumor cellularity as measured by the ratio of cells to collagen in trichrome staining (Fig.6G). Collectively, our results confirm the synergy of the combination of Hsp90 and Erk5 inhibitors *in vivo* which could be explained by significant induction of apoptotic cell death and tumor tissue degeneration.

## DISCUSSION

Proteomic profiling of high-grade breast cancers using the Kinex™ antibody microarrays confirmed the heterogeneity of breast cancer in general with the identification of at least six subgroups. Particularly, TNBC is heterogeneous at the proteomic level where one subgroup of TNBC showed a wide spectrum of activated oncogenic signaling pathways in comparison to other TNBC tumors as well as non-TNBC subtypes. The activated signaling pathways we found in this TNBC subgroup (cluster ii) did not associate with increased mRNA expression in published gene expression microarray datasets. Our results, and others [5, 13, 14], revealed that the proteomic fingerprint classifies breast cancer to similar subtypes as transcriptome profiling; however, the two fingerprints are not identical.

The TNBC tumors in our proteomic study divided across four different tumor clusters with strong differences at the protein and phosphoprotein levels. Our finding is in line with previous studies reporting heterogeneity within TNBC. Immunophenotypically, TNBC can be at least divided into basal-like and non-basal like groups [27]. At the transcriptome level, while the majority (~ 70%) of TNBC tumors are classified as basal-like, the remainder are spread across the other intrinsic subtypes; luminal A and B, Her2-enriched and normal-like [28]. The extent of TNBC heterogeneity has been further characterized by Lehmann et al. [29] in a study which identified six molecular subtypes of TNBC based on gene expression analysis. Similarly, we found that 9 out of 15 (60%) TNBC tumors clustered together (clusters i and ii) whereas the rest (6 out of 15, 40%) were admixed with hormone-positive or HER2-positive tumors (clusters iii and iv) [5]. Similarly, we found that 9 out of 15 (60%) TNBC tumors clustered together (clusters i and ii) whereas the rest (6 out of 15, 40%) clustered closer to hormone-positive or HER2-positive tumors (clusters iii and iv). In our study, the TNBC tumors in cluster ii were related to TNBC cell lines that are classified as mesenchymal- and mesenchymal-stem like at the transcriptome level [29]. These tumors and cell lines showed robust signaling cascades with cross-talking canonical signaling pathways some of which have been previously described as targets for TNBC such as ERK/MAPK [11], JAK/STAT signaling [30, 31] and PI3K/AKT/PTEN [32, 33].

In an effort to translate our findings into therapeutic strategies, we showed that TNBC cell lines were sensitive to several inhibitors which were selected based on the activated signaling pathways identified by proteomic profiling. Similar differential responses of breast cancer cell lines, according to subtypes and/or pathways, to therapeutics have been previously reported [34]. Our study only shared three inhibitors with the previous study [34] which target MEK or EGFR and our results are in agreement that TNBC cell lines are more sensitive to these inhibitors than luminal breast cancer cell lines. The advantage of our study is that the selection of drugs was envisaged by proteomic characterization of pathway activation. Moreover, we identified synergistic effects when combining activated kinase inhibitors with inhibitors of the chaperone Hsp90. The heat shock protein Hsp90 has been described as a therapeutic target in TNBC [26] and we recognized that not only Hsp90 was upregulated in TNBC tumors and cell lines in our study, but also that several of the activated kinases we identified are onco-clients of Hsp90. We hypothesized that the inhibition of Hsp90 would lead to inhibition of several signaling pathways identified in our study and that the additional inhibition of specific pathway would lead to enhanced toxicity in TNBC cells. Indeed, we found the combined inhibition of Hsp90 and one of 11 other kinases was synergistic, particularly, the combination of Hsp90 and Erk5 inhibition.

We focused on Erk5 as a novel target in TNBC. Erk5 regulates the Nrf2 (NFE2L2) transcription factor [35] which was also upregulated in TNBC in our study. Activated Erk5 is not a client of Hsp90 [25] and the Erk5/Nrf2-mediated oxidative stress response pathway is activated in response to Hsp90 inhibition [22]. Erk5 has been suggested as a target for cancer therapy [23, 24]. Using breast cancer cell lines, studies showed that Erk5 plays roles in cell proliferation by regulating cyclin D1 and CDKs [36-38], epithelial mesenchymal transition [39], MET/HGF-induced migration [40, 41] and integrin/FAK-mediated metastasis [42]. The MEK5-Erk5 pathway mediates progression to an ER-negative, mesenchymal and endocrine therapy resistant phenotype [43]. Experimentally, the knockdown of MEKK2-MEK5-Erk5 pathway affects the primary tumor growth and metastasis in xenografts model of the TNBC cell line MDA-MB-231 [44]. Clinically, Erk5 protein is overexpressed in early stage breast cancer and associated with disease free survival [45]. In a recent study [43], activated (phosphorylated) Erk5 was found in 77% of breast tumors in comparison to adjacent tissue. In our previous study of aggressive breast tumors which metastasized to the brain [46], we also found activated Erk5 in 78% of these tumors and more importantly, we found activated Erk5 in 100% of brain metastasis from breast tumors (*p = 0.032* cf. 78% in matched primary breast tumors [46]). Altogether, Erk5 is an attractive therapeutic target in breast cancer, particularly in TNBC. To our knowledge, we are the first to report targeting Erk5 using a molecular inhibitor *in vivo* in breast cancer. Our *in vivo* study demonstrated the utility of Erk5 inhibition as a therapeutic strategy alone or in combination with chemotherapy and more so when combined with Hsp90 inhibition in TNBC.

In conclusion, we report the first proteomic study of breast cancer using the Kinex™ antibody arrays that can interrogate several hundred more proteins and phosphoproteins than RPPA and in a less complex fashion than mass spectroscopy-based proteomics. The proteomic-fingerprint from the Kinex™ antibody arrays, similar to RPPA- and MS-based proteomics, identified subtypes within breast cancer that reflect transcriptome profiling; however, the proteomic- and transcriptome-fingerprints were not necessarily identical. The more direct translational path of proteomic profiling for identifying therapeutic targets was illustrated by the identification of several inhibitors which affect the survival of the aggressive TNBC cell lines. Our *in vivo* studies support the notion that the inhibition of several oncogenic pathways is required for making an impact against TNBCs which exploit cross-talking pathways to drive their aggressive phenotype.

## MATERIALS AND METHODS

### Primary breast tumor collection and cell culture

The Brisbane Breast Bank collected fresh breast tumor samples from consenting patients; the study was approved by the local research ethics committees. Clinical, histopathological and the status for estrogen [ER] and progesterone [PR] receptors and HER2 were obtained from pathology reports (Supplementary Table S1). The cut off for ER and PR positivity was 1% and the HER2 status was defined using the HercepTest™ (Dako). Breast cancer cell lines from ATCC™ (VA, USA) were cultured as per ATCC™ instruction and tested for mycoplasma and authenticated using STR profiling.

### Kinex™ antibody and Proteome Profiler™ arrays

Fresh frozen primary breast tumors and fresh frozen cell lines were lysed using the Kinex™ lysis buffer as per the manufacturer instructions (Kinexus Corporation, Vancouver, Canada). Dye-labeling of protein lysates, hybridization to the Kinex™ antibody array (KAM-1.3 Antibody Microarray) and signal intensity extraction were performed by Kinexus Corporation. Signal intensities for the 812 probes (in technical replicates), which included pan-specific antibodies for total proteins and phospho-specific antibodies against specific phosphorylations, were analyzed using BRB-ArrayTools [47]. Signal intensities were quantile-normalized to perform unsupervised hierarchical clustering and Significance Analysis of Microarray (SAM). For the Proteome Profiler™ arrays, lysates from cell lines were hybridized to the arrays and phosphorylation levels were detected and quantified as per manufacturer instructions (R&D Systems, MN, USA).

### Ingenuity pathway analysis and Oncomine™ analysis

Pathway analysis was performed using the Ingenuity Pathway Analysis® (IPA) software (Ingenuity Systems®, Redwood City, CA) and we limited the analysis to direct protein-protein interactions only to identify signaling networks and canonical signaling pathways. The Oncomine™ database [48] (Compendia Bioscience, Ann Arbor, MI), a large consortium of gene expression microarray datasets, was interrogated for the mRNA expression levels of proteins identified in our screen.

### *In vitro* drug sensitivity screens

Small molecule inhibitors were purchased from Tocris Biosciences (R&D Systems): PF-4708671, U 73122, GSK2334470, IPA3, SL0101-1 and XMD 8-92 or from Selleck Chemicals LLC (TX, USA): PF-562271, Enzastaurin (LY317615), AUY922 (NVP-AUY922), 17-AAG (Geldanamycin), PF-04929113 (SNX-5422), AZD6244 (Selumetinib), AT7867, CHIR-98014, LY2228820, BIX 02188, AS703026, PH-797804, SP600125, NU7441. All inhibitors were prepaed in DMSO at 100 mM. Cells were treated with inhibitors prepared in culture media where the final concentration of DMSO was 2% v/v. Vehicle control treatments consisted of culture media containing 2% v/v DMSO. Six days after treatment, cell survival was measured in comparison to vehicle controls using the CellTiter 96® Assay as per manufacturer instructions (MTS assay, Promega Corporation, WI, USA). Data were analyzed in GraphPad Prism® version 5.00 for Windows (GraphPad Software, CA, USA) to measure the log_10_ of IC50 for each drug. For combination assays, drugs were added simultaneously. Heatmaps for sensitivities (-log_10_ of IC50) were prepared using D-chip Analyzer software [49].

### *In vivo* models and treatments

The local animal ethics committee gave approval for use of the mice. Female balb/c nude mice at 5 weeks of age (Animal Resources Centre, WA, Australia) were inoculated in the mammary fat-pads with exponentially growing MDA-MB-231 TNBC cells (5×10^6^ per fat pad) in 50 µL of 50:50 PBS:Matrigel™ (BD Biosciences, CA, USA). Treatments started when tumors were 50 ± 1 mm^3^ as calculated from caliper measurement of tumor's longest (a) and shortest (b) diameters: tumor volume (mm^3^) = a/2 × b^2^. Docetaxel, doxorubicin and the Hsp90 inhibitor AUY922 were purchased from Selleck Chemicals and the Erk5 inhibitor XMD 8-92 from Tocris Biosciences. All drugs were diluted in 5% solution of D-glucose in PBS for intraperitoneal injection. Doses and schedule of treatments are detailed in Figure legends. Mice were monitored for change in weight and other toxicity indications and none were observed. Tumor growth was monitored by caliper measurements to calculate the change in tumor volume compared to day 0. Six tumors were excised on day 13 to perform *ex vivo* studies as below.

### In *vitro* and *ex vivo* studies for the combination of *Hsp90* and *Erk5* inhibition

For the combination index studies *in vitro*, cultures of TNBC cell lines were left untreated or treated with escalating doses of the Erk5 inhibitor alone, Hsp90 inhibitor alone or the combinations. After 6 days of treatment, the MTS assay was performed to measure survival in comparison to control untreated cells. The combination index (CI) was calculated for each combination according to Chou [50]. For immunoblot assays, the MDA-MB-231 cells were treated with 1 µM Erk5i alone, 2 nM of Hsp90i alone, chemotherapy alone (2.5 nM docetaxel and 10 nM doxorubicin) or the combinations. After 24 hours of treatments, lysates were prepared and standard immunobloting was performed. For *ex vivo* studies, tumors excised from mice on day 13 (after treatment completion) were bisected where one half was fixed in 10% saline-buffered formalin solution and the second half was used to prepare protein lysates.

Standard immunoblots were performed on lysates from cells grown in vitro and from tumors excised after treatments. The rabbit polyclonal antibodies against Erk5, phospho-Erk5 (Thr218/Tyr220), Hsp70, Hsp90, Erk1/2 and phosphor-Erk1/2 (Thr202/Tyr204) were purchased from Cell Signaling (MA, USA). The rabbit monoclonal antibodies against Bak and Bim were from Epitomics (CA, USA). The mouse monoclonal antibodies against Bcl-xL and β-actin were from BD Biosciences (CA, USA). The rabbit monoclonal antibody against COXIV was purchased from LI-COR Biosciences (NE, USA). Secondary antibodies against mouse and rabbit IgG conjugated to HRP were from Cell Signaling and blots were developed using developed using the chemiluminescence reagent plus (Millipore, MA, USA).

Formalin fixed, paraffin embedded tumors were used for standard H&E or Masson's trichrome staining. Immunohistochemical staining (IHC) was performed to detect double strand DNA breaks (DSBs) using the anti-phospho-histone H2AX (Ser139, γH2AX) mAb (clone JBW301, Merk Millipore. MA, USA) or apoptosis using ApopTag® Peroxidase In Situ Apoptosis Detection Kit (Merk Millipore) as per manufacturer instructions. For IHC quantification, images of ten random regions from each tumor section were analyzed using ImageJ [51] (V1.46d) with ImmunoRatio plugin [52]. Masson's trichrome was analyzed using ImageJ as previously described [53] using images of ten random regions from each tumor section. Standard immunoblotting was performed using lysates prepared from freshly isolated tumors.

### Statistical analysis

Statistical analysis of the Kinex™ antibody arrays was performed in ArrayTools as described above. Other statistical analyses were performed in GraphPad Prism®.

## SUPPLEMENTARY FIGURES AND TABLES





## References

[1] Sorlie T, Perou CM, Tibshirani R, Aas T, Geisler S, Johnsen H, Hastie T, Eisen MB, van de Rijn M, Jeffrey SS, Thorsen T, Quist H, Matese JC, Brown PO, Botstein D, Eystein Lonning P (2001). Gene expression patterns of breast carcinomas distinguish tumor subclasses with clinical implications. Proceedings of the National Academy of Sciences of the United States of America.

[2] Perou CM, Sorlie T, Eisen MB, van de Rijn M, Jeffrey SS, Rees CA, Pollack JR, Ross DT, Johnsen H, Akslen LA, Fluge O, Pergamenschikov A, Williams C, Zhu SX, Lonning PE, Borresen-Dale AL (2000). Molecular portraits of human breast tumours. Nature.

[3] Curtis C, Shah SP, Chin SF, Turashvili G, Rueda OM, Dunning MJ, Speed D, Lynch AG, Samarajiwa S, Yuan Y, Graf S, Ha G, Haffari G, Bashashati A, Russell R, McKinney S (2012). The genomic and transcriptomic architecture of 2,000 breast tumours reveals novel subgroups. Nature.

[4] Wulfkuhle JD, Speer R, Pierobon M, Laird J, Espina V, Deng J, Mammano E, Yang SX, Swain SM, Nitti D, Esserman LJ, Belluco C, Liotta LA, Petricoin EF (2008). Multiplexed cell signaling analysis of human breast cancer applications for personalized therapy. J Proteome Res.

[5] Hennessy BT, Lu Y, Gonzalez-Angulo AM, Carey MS, Myhre S, Ju Z, Davies MA, Liu W, Coombes K, Meric-Bernstam F, Bedrosian I, McGahren M, Agarwal R, Zhang F, Overgaard J, Alsner J (2010). A Technical Assessment of the Utility of Reverse Phase Protein Arrays for the Study of the Functional Proteome in Non-microdissected Human Breast Cancers. Clin Proteomics.

[6] TCGA (2012). Comprehensive molecular portraits of human breast tumours. Nature.

[7] Gonzalez-Angulo AM, Hennessy BT, Meric-Bernstam F, Sahin A, Liu W, Ju Z, Carey MS, Myhre S, Speers C, Deng L, Broaddus R, Lluch A, Aparicio S, Brown P, Pusztai L, Symmans WF (2011). Functional proteomics can define prognosis and predict pathologic complete response in patients with breast cancer. Clin Proteomics.

[8] Gonzalez-Angulo AM, Liu S, Chen H, Chavez-Macgregor M, Sahin A, Hortobagyi GN, Mills GB, Do KA, Meric-Bernstam F (2013). Functional proteomics characterization of residual breast cancer after neoadjuvant systemic chemotherapy. Ann Oncol.

[9] Sohn J, Do KA, Liu S, Chen H, Mills GB, Hortobagyi GN, Meric-Bernstam F, Gonzalez-Angulo AM (2013). Functional proteomics characterization of residual triple-negative breast cancer after standard neoadjuvant chemotherapy. Ann Oncol.

[10] Bateman NW, Sun M, Bhargava R, Hood BL, Darfler MM, Kovatich AJ, Hooke JA, Krizman DB, Conrads TP (2011). Differential proteomic analysis of late-stage and recurrent breast cancer from formalin-fixed paraffin-embedded tissues. J Proteome Res.

[11] Duncan JS, Whittle MC, Nakamura K, Abell AN, Midland AA, Zawistowski JS, Johnson NL, Granger DA, Jordan NV, Darr DB, Usary J, Kuan PF, Smalley DM, Major B, He X, Hoadley KA (2012). Dynamic reprogramming of the kinome in response to targeted MEK inhibition in triple-negative breast cancer. Cell.

[12] Cabezon T, Gromova I, Gromov P, Serizawa R, Timmermans Wielenga V, Kroman N, Celis JE, Moreira JM (2013). Proteomic Profiling of Triple-negative Breast Carcinomas in Combination With a Three-tier Orthogonal Technology Approach Identifies Mage-A4 as Potential Therapeutic Target in Estrogen Receptor Negative Breast Cancer. Mol Cell Proteomics.

[13] Hochgrafe F, Zhang L, O'Toole SA, Browne BC, Pinese M, Porta Cubas A, Lehrbach GM, Croucher DR, Rickwood D, Boulghourjian A, Shearer R, Nair R, Swarbrick A, Faratian D, Mullen P, Harrison DJ (2010). Tyrosine phosphorylation profiling reveals the signaling network characteristics of Basal breast cancer cells. Cancer research.

[14] Kennedy JJ, Abbatiello SE, Kim K, Yan P, Whiteaker JR, Lin C, Kim JS, Zhang Y, Wang X, Ivey RG, Zhao L, Min H, Lee Y, Yu MH, Yang EG, Lee C (2013). Demonstrating the feasibility of large-scale development of standardized assays to quantify human proteins. Nat Methods [Epub ahead of print].

[15] Tian Q, Stepaniants SB, Mao M, Weng L, Feetham MC, Doyle MJ, Yi EC, Dai H, Thorsson V, Eng J, Goodlett D, Berger JP, Gunter B, Linseley PS, Stoughton RB, Aebersold R (2004). Integrated genomic and proteomic analyses of gene expression in Mammalian cells. Mol Cell Proteomics.

[16] Brockmann R, Beyer A, Heinisch JJ, Wilhelm T (2007). Posttranscriptional expression regulation: what determines translation rates?. PLoS Comput Biol.

[17] Zhang H, Pelech S (2012). Using protein microarrays to study phosphorylation-mediated signal transduction. Semin Cell Dev Biol.

[18] Astanehe A, Finkbeiner MR, Krzywinski M, Fotovati A, Dhillon J, Berquin IM, Mills GB, Marra MA, Dunn SE (2012). MKNK1 is a YB-1 target gene responsible for imparting trastuzumab resistance and can be blocked by RSK inhibition. Oncogene.

[19] Neve RM, Chin K, Fridlyand J, Yeh J, Baehner FL, Fevr T, Clark L, Bayani N, Coppe JP, Tong F, Speed T, Spellman PT, DeVries S, Lapuk A, Wang NJ, Kuo WL (2006). A collection of breast cancer cell lines for the study of functionally distinct cancer subtypes. Cancer cell.

[20] Moulick K, Ahn JH, Zong H, Rodina A, Cerchietti L, Gomes DaGama EM, Caldas-Lopes E, Beebe K, Perna F, Hatzi K, Vu LP, Zhao X, Zatorska D, Taldone T, Smith-Jones P, Alpaugh M (2011). Affinity-based proteomics reveal cancer-specific networks coordinated by Hsp90. Nat Chem Biol.

[21] Taipale M, Krykbaeva I, Koeva M, Kayatekin C, Westover KD, Karras GI, Lindquist S (2012). Quantitative analysis of HSP90-client interactions reveals principles of substrate recognition. Cell.

[22] Wu Z, Moghaddas Gholami A, Kuster B (2012). Systematic identification of the HSP90 candidate regulated proteome. Mol Cell Proteomics.

[23] Drew BA, Burow ME, Beckman BS (2012). MEK5/ERK5 pathway: the first fifteen years. Biochimica et biophysica acta.

[24] Yang Q, Lee JD (2011). Targeting the BMK1 MAP kinase pathway in cancer therapy. Clin Cancer Res.

[25] Erazo T, Moreno A, Ruiz-Babot G, Rodriguez-Asiain A, Morrice NA, Espadamala J, Bayascas JR, Gomez N, Lizcano JM (2013). Canonical and kinase activity-independent mechanisms for extracellular signal-regulated kinase 5 (ERK5) nuclear translocation require dissociation of Hsp90 from the ERK5-Cdc37 complex. Molecular and cellular biology.

[26] Caldas-Lopes E, Cerchietti L, Ahn JH, Clement CC, Robles AI, Rodina A, Moulick K, Taldone T, Gozman A, Guo Y, Wu N, de Stanchina E, White J, Gross SS, Ma Y, Varticovski L (2009). Hsp90 inhibitor PU-H71, a multimodal inhibitor of malignancy, induces complete responses in triple-negative breast cancer models. Proceedings of the National Academy of Sciences of the United States of America.

[27] Choi YL, Oh E, Park S, Kim Y, Park YH, Song K, Cho EY, Hong YC, Choi JS, Lee JE, Kim JH, Nam SJ, Im YH, Yang JH, Shin YK (2010). Triple-negative, basal-like, and quintuple-negative breast cancers: better prediction model for survival. BMC cancer.

[28] Prat A, Perou CM (2011). Deconstructing the molecular portraits of breast cancer. Mol Oncol.

[29] Lehmann BD, Bauer JA, Chen X, Sanders ME, Chakravarthy AB, Shyr Y, Pietenpol JA (2011). Identification of human triple-negative breast cancer subtypes and preclinical models for selection of targeted therapies. The Journal of clinical investigation.

[30] Marotta LL, Almendro V, Marusyk A, Shipitsin M, Schemme J, Walker SR, Bloushtain-Qimron N, Kim JJ, Choudhury SA, Maruyama R, Wu Z, Gonen M, Mulvey LA, Bessarabova MO, Huh SJ, Silver SJ (2011). The JAK2/STAT3 signaling pathway is required for growth of CD44(+)CD24(-) stem cell-like breast cancer cells in human tumors. The Journal of clinical investigation.

[31] Britschgi A, Andraos R, Brinkhaus H, Klebba I, Romanet V, Muller U, Murakami M, Radimerski T, Bentires-Alj M (2012). JAK2/STAT5 Inhibition Circumvents Resistance to PI3K/mTOR Blockade: A Rationale for Cotargeting These Pathways in Metastatic Breast Cancer. Cancer cell.

[32] Adamo B, Deal AM, Burrows E, Geradts J, Hamilton E, Blackwell KL, Livasy C, Fritchie K, Prat A, Harrell JC, Ewend MG, Carey LA, Miller CR, Anders CK (2011). Phosphatidylinositol 3-kinase (PI3K) pathway activation in breast cancer brain metastases. Breast Cancer Res.

[33] Gordon V, Banerji S (2013). Molecular pathways: PI3K pathway targets in triple-negative breast cancers. Clin Cancer Res.

[34] Heiser LM, Sadanandam A, Kuo WL, Benz SC, Goldstein TC, Ng S, Gibb WJ, Wang NJ, Ziyad S, Tong F, Bayani N, Hu Z, Billig JI, Dueregger A, Lewis S, Jakkula L (2012). Subtype and pathway specific responses to anticancer compounds in breast cancer. Proceedings of the National Academy of Sciences of the United States of America.

[35] Kim M, Kim S, Lim JH, Lee C, Choi HC, Woo CH (2012). Laminar flow activation of ERK5 protein in vascular endothelium leads to atheroprotective effect via NF-E2-related factor 2 (Nrf2) activation. The Journal of biological chemistry.

[36] Esparis-Ogando A, Diaz-Rodriguez E, Montero JC, Yuste L, Crespo P, Pandiella A (2002). Erk5 participates in neuregulin signal transduction and is constitutively active in breast cancer cells overexpressing ErbB2. Molecular and cellular biology.

[37] Mulloy R, Salinas S, Philips A, Hipskind RA (2003). Activation of cyclin D1 expression by the ERK5 cascade. Oncogene.

[38] Perez-Madrigal D, Finegan KG, Paramo B, Tournier C (2012). The extracellular-regulated protein kinase 5 (ERK5) promotes cell proliferation through the down-regulation of inhibitors of cyclin dependent protein kinases (CDKs). Cellular signalling.

[39] Zhou C, Nitschke AM, Xiong W, Zhang Q, Tang Y, Bloch M, Elliott S, Zhu Y, Bazzone L, Yu D, Weldon CB, Schiff R, McLachlan JA, Beckman BS, Wiese TE, Nephew KP (2008). Proteomic analysis of tumor necrosis factor-alpha resistant human breast cancer cells reveals a MEK5/Erk5-mediated epithelial-mesenchymal transition phenotype. Breast Cancer Res.

[40] Locatelli A, Lange CA (2011). Met receptors induce Sam68-dependent cell migration by activation of alternate extracellular signal-regulated kinase family members. The Journal of biological chemistry.

[41] Castro NE, Lange CA (2010). Breast tumor kinase and extracellular signal-regulated kinase 5 mediate Met receptor signaling to cell migration in breast cancer cells. Breast Cancer Res.

[42] Sawhney RS, Liu W, Brattain MG (2009). A novel role of ERK5 in integrin-mediated cell adhesion and motility in cancer cells via Fak signaling. Journal of cellular physiology.

[43] Antoon JW, Martin EC, Lai R, Salvo VA, Tang Y, Nitzchke AM, Elliott S, Nam SY, Xiong W, Rhodes LV, Collins-Burow B, David O, Wang G, Shan B, Beckman BS, Nephew KP (2013). MEK5/ERK5 signaling suppresses estrogen receptor expression and promotes hormone-independent tumorigenesis. PLoS ONE.

[44] Cronan MR, Nakamura K, Johnson NL, Granger DA, Cuevas BD, Wang JG, Mackman N, Scott JE, Dohlman HG, Johnson GL (2012). Defining MAP3 kinases required for MDA-MB-231 cell tumor growth and metastasis. Oncogene.

[45] Montero JC, Ocana A, Abad M, Ortiz-Ruiz MJ, Pandiella A, Esparis-Ogando A (2009). Expression of Erk5 in early stage breast cancer and association with disease free survival identifies this kinase as a potential therapeutic target. PLoS ONE.

[46] Da Silva L, Simpson PT, Smart CE, Cocciardi S, Waddell N, Lane A, Morrison BJ, Vargas AC, Healey S, Beesley J, Pakkiri P, Parry S, Kurniawan N, Reid L, Keith P, Faria P (2010). HER3 and downstream pathways are involved in colonization of brain metastases from breast cancer. Breast Cancer Res.

[47] Zhao Y, Simon R (2008). BRB-ArrayTools Data Archive for human cancer gene expression: a unique and efficient data sharing resource. Cancer Inform.

[48] Rhodes DR, Yu J, Shanker K, Deshpande N, Varambally R, Ghosh D, Barrette T, Pandey A, Chinnaiyan AM (2004). ONCOMINE: a cancer microarray database and integrated data-mining platform. Neoplasia (New York, NY.

[49] Li C, Wong WH (2001). Model-based analysis of oligonucleotide arrays: expression index computation and outlier detection. Proceedings of the National Academy of Sciences of the United States of America.

[50] Chou TC (2006). Theoretical basis, experimental design, and computerized simulation of synergism and antagonism in drug combination studies. Pharmacological reviews.

[51] Abramoff MD, Magalhaes PJ, Ram SJ (2004). Image Processing with ImageJ. Biophonotics International.

[52] Tuominen VJ, Ruotoistenmaki S, Viitanen A, Jumppanen M, Isola J (2010). ImmunoRatio: a publicly available web application for quantitative image analysis of estrogen receptor (ER), progesterone receptor (PR), and Ki-67. Breast Cancer Res.

[53] Collins TJ (2007). ImageJ for microscopy. Biotechniques.

