# Supplementary material for: Kinome profiling reveals breast cancer heterogeneity and identifies targeted therapeutic opportunities for triple negative breast cancer
Source: Oncotarget. 2014 Mar 26;5(10):3145–58. doi: 10.18632/oncotarget.1865 (PMC4102798; doi:10.18632/oncotarget.1865)
Supplement: Supplementary file 1 [file oncotarget-05-3145-s001.pdf]

Kinome profiling reveals breast cancer heterogeneity and identifies targeted therapeutic opportunities for triple negative breast cancer - Al-Ejeh et al

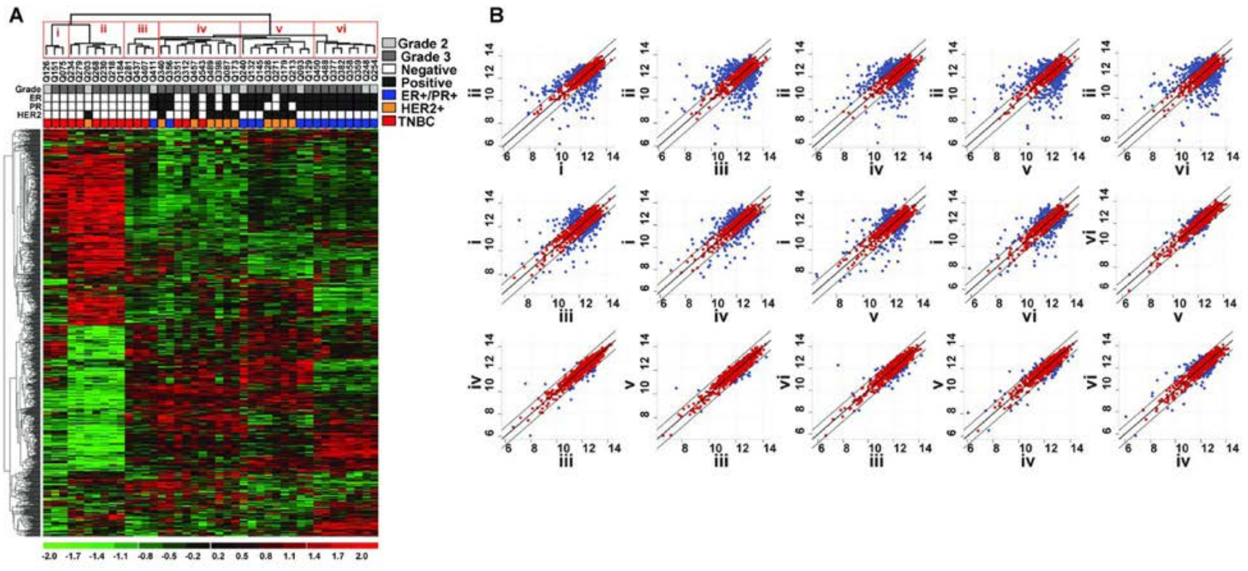

Supplementary Figure 1: Kinex^{TM} antibody arrays on lysates from 41 primary breast tumors. Lysates from fresh frozen primary breast tumors (listed in Supplementary Table 1S) were used for the Kinex^{TM} KAM 1.3 antibody arrays. (**A**) After quantile normalization, the arrays were visualized using ArrayTools by unsupervised hierarchal clustering of both samples and probes. All probes (812 probes) were used in the clustering and 6 sample clusters (i - vi) were observed. (**B**) Dot plots for the comparisons of clusters i to vi showing all 812 probes on the Kinex^{TM} arrays with probes showing more than 1.75-fold up-regulation or down-regulation marked in blue. The data is the quantile normalized log2 expression values and the diagonal middle line in each plot represents 1:1 ratio and the lines on the sides of the middle diagonal line represents the 1.75-fold cutoff lines. Significance analysis of microarray (SAM) was performed on the entire array in A using ArrayTools then filtered for proteins and phosphoproteins with more than 1.75-fold deregulation between any two groups. The results from this analysis are shown in Supplementary Table 2S and were used for clustering in Fig.1B.

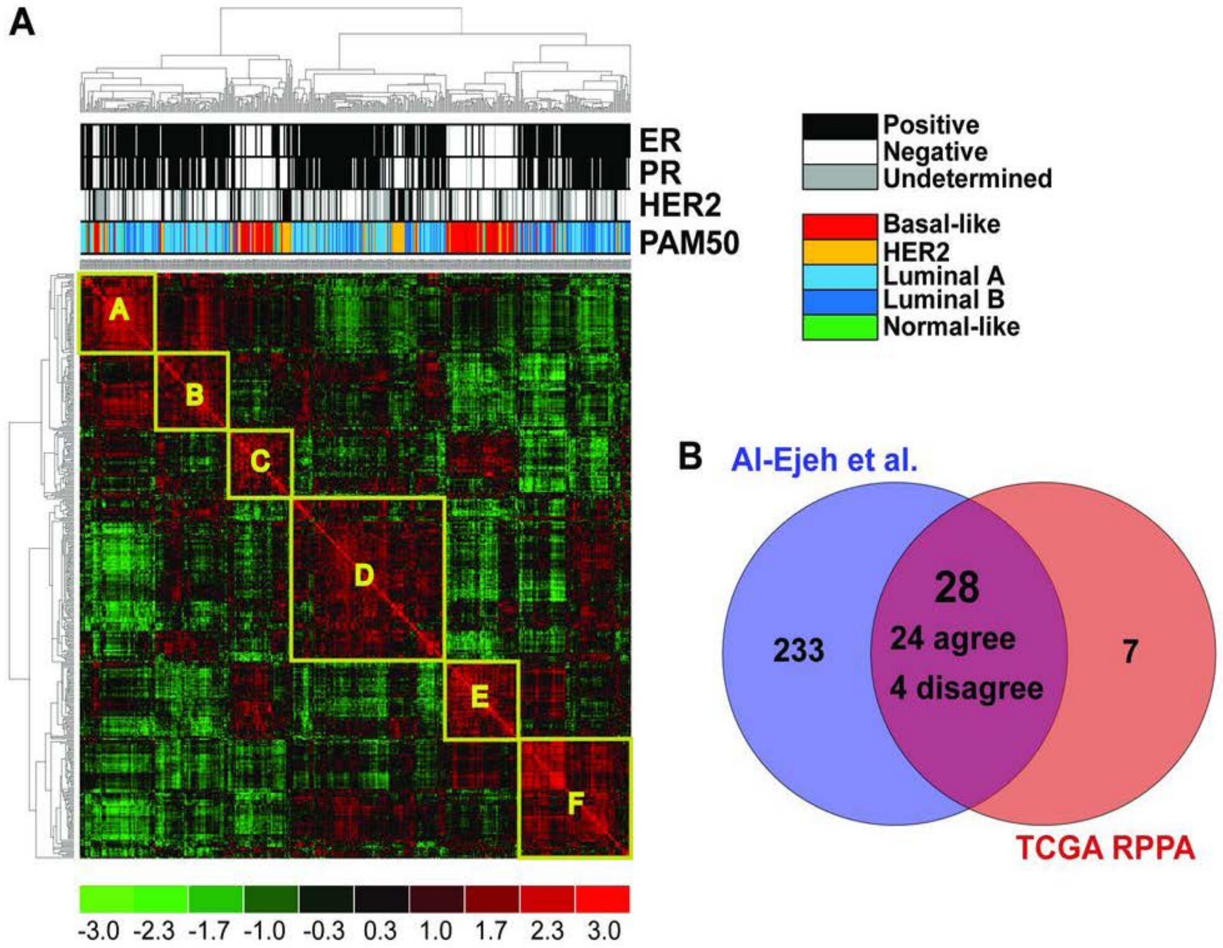

Supplementary Figure 2: Heterogeneity of breast cancer based on the TCGA RPPA data. The RPPA data from the breast cancer TCGA study (https://tcga-data.nci.nih.gov/docs/publications/brca_2012/) and analyzed identically to our analysis of the Kinex^{TM} antibody arrays (Figure 1 in article). (**A**) Sample-sample correlation plot using all the probes revealed six clusters in the TCGA RPPA data. TNBC (ER/PR/HER2 negative) primary tumors appeared in two main clusters (C and E) and to a lesser extent in cluster A. The tumors used from the TCGA RPPA were also annotated for their PAM50 subtypes and this information was also overlaid on the sample-sample correlation heat map. The basal-like breast cancer subtype, which is enriched in TNBC tumors also followed the three clusters (A, C and E). We compared tumors in clusters C and E to all other clusters and to each other with 1.5-fold change cutoff to identify deregulated proteins and phosphoproteins in these clusters compared to other clusters (Supplementary Table 3). There were 65 probes (total of 61 proteins/phosphoproteins) which were deregulated in tumors from cluster C or E compared to tumors from at least one other cluster. The Kinex^{TM} array contained probes for the detection of 35 out of the 61 deregulated proteins/phosphoproteins. (**B**) Venn diagram showing the overlap between our study and the TCGA RPPA data for proteins and phosphoproteins, which were deregulated in TNBC sample groups. The 28 common proteins and phosphoproteins are labeled in Supplementary Table 3 and the concordance between our results and the TCGA RPPA data is marked (reached 85%); 24 proteins/phosphoproteins were deregulated in TNBC in our study and the TCGA RPPA in the same direction whereas 4 proteins/phosphoproteins were deregulated in opposite directions in the two studies. The remaining 11 proteins/phosphoproteins in the TCGA RPPA study did not reach the cutoff threshold in our Kinex^{TM} arrays.

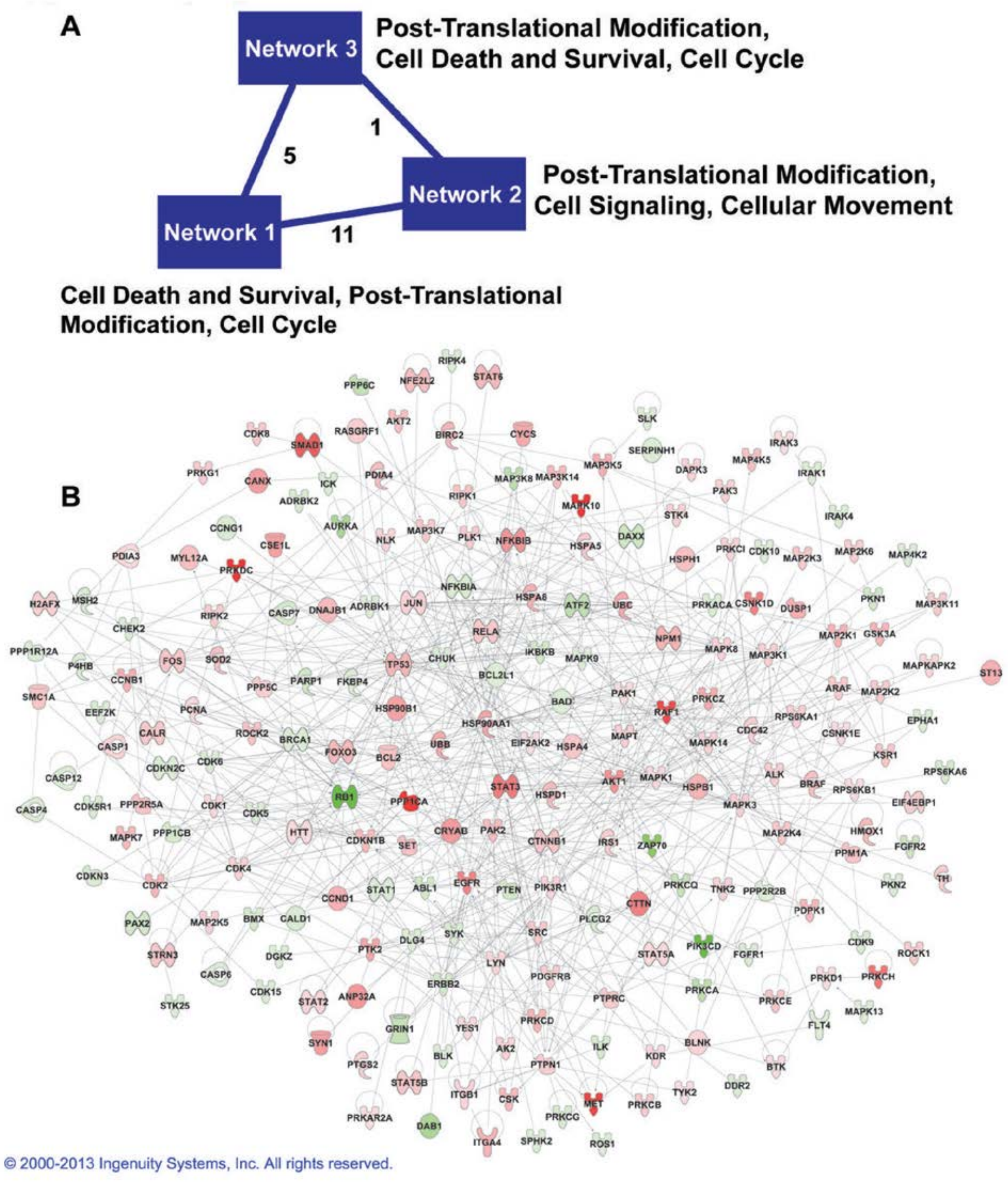

Supplementary Figure 3: Ingenuity Pathway Analysis (IPA®) of deregulated proteins/phosphoproteins in cluster ii subgroup. Pathway analysis of the upregulated (red) and downregulated (green) proteins and phosphoproteins was performed using IPA® limiting the analysis to direct protein-protein interactions only. (**A**) Three overlapping networks were identified (numbers at the lines connecting the networks show the number of overlapping proteins between these networks). (**B**) The overlapping networks were merged to represent a major network of upregulated and down-regulated proteins/phosphoproteins. This major network was also visualized for upregulated proteins/phosphoproteins only which was presented in Fig.3.

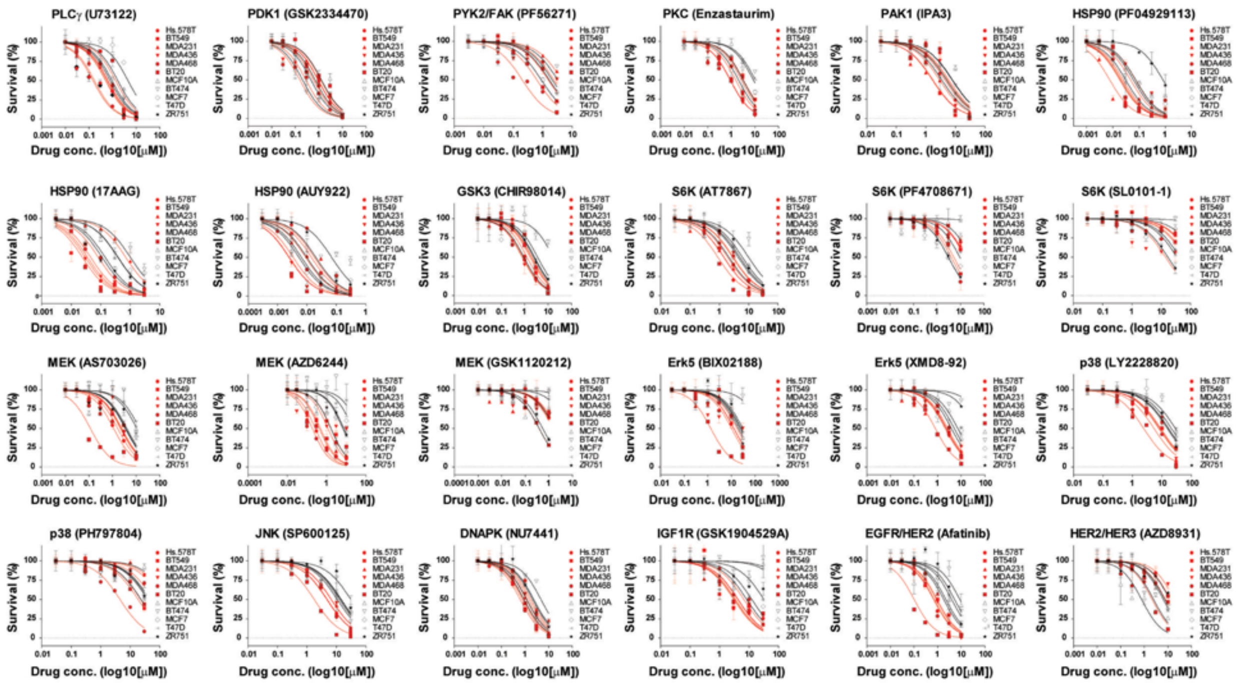

Supplementary Figure 4: Treatment of TNBC and non-TNBC cell lines with targeted inhibitors against kinases identified in cluster ii. Cultures of six TNBC cell lines (resembling cluster ii) and four non-TNBC cell lines (resembling cluster vi) and the "near-normal" MCF10A cell line were treated with escalating doses of the specified drugs which were selected based on upregulated kinases in cluster ii vs. cluster vi. Six days after treatment the CellTitre^{TM} MTS/MTA assay was performed to compare the survival of cells to cells incubated with vehicle control (2% DMSO). Graphs were constructed using GraphPad$^{®}$ Prism using a dose-response curve equation to measure the inhibitory concentration that kills 50% of cells (IC50). The graphs produced the log10[IC50] which was inverted and used to generate the heat maps shown in Fig. 4.

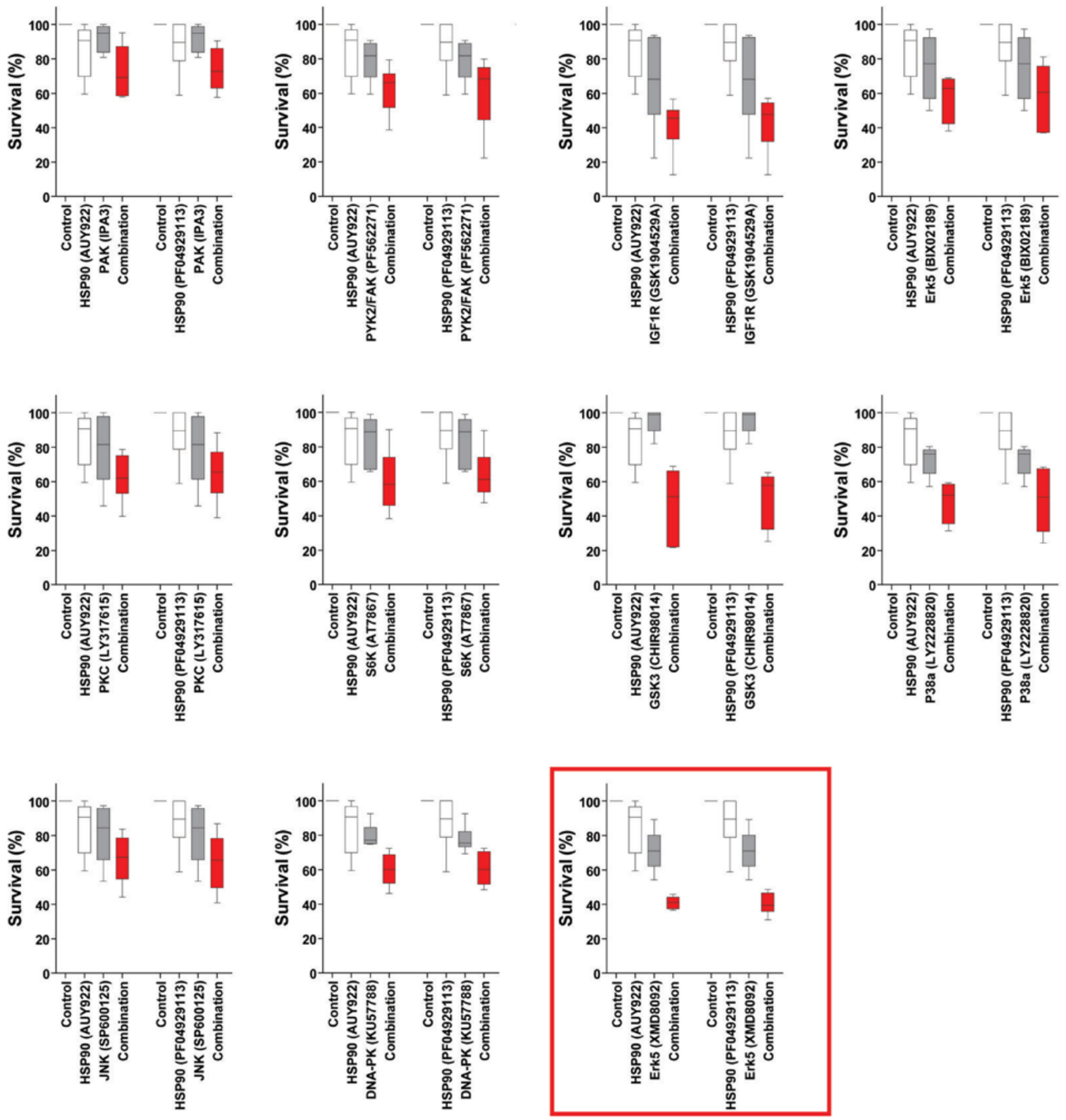

Supplementary Figure 5: The combination of Hsp90 inhibitors with inhibitors of other activated kinases in cluster ii tumors. Culture of six TNBC cell lines were left untreated (Control, vehicle 2% DMSO) or treated with Hsp90 inhibitors alone (white boxes), kinase inhibitors alone (gray boxes) or the combination (red boxes). The IC25 of single drugs were used alone and in combinations. Only combinations which showed statistical differences ($p < 0.05$, One-Way Anova, GraphPad® Prism) are shown. Box plots show the mean survival from the six TNBC cell lines with minimum and maximum spread shown. The combination of Hsp90 inhibitors with the Erk5 inhibitor XMD 8-92 (bottom right corner) showed the most significant and consistent synergy across the six cell lines. Other combinations were also synergistic, particularly the combinations of Hsp90 inhibitors with DNA-PK, p38a MAPK and GSK3a/b and could be promising combination therapies against TNBC.

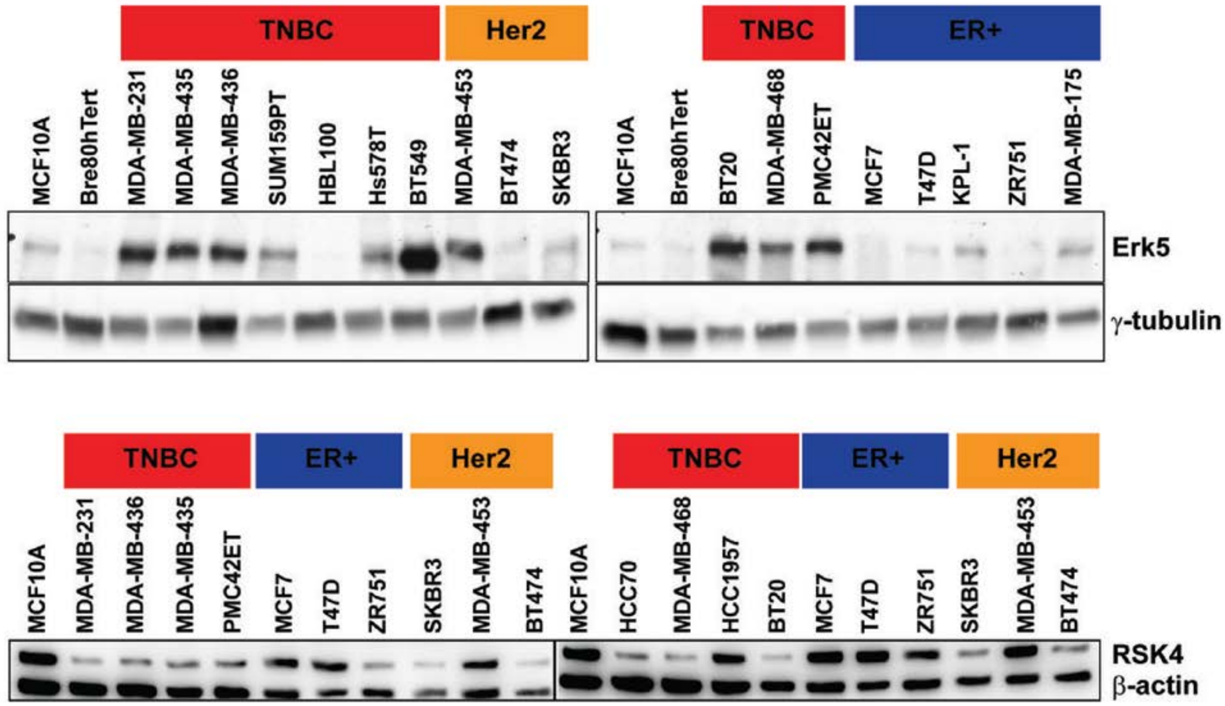

Supplementary Figure 6: Erk5 is upregulated in TNBC cell lines. The near normal MCF10A cells, the immortalized human mammary epithelial cell line Bre80-hTERT, and the panel of breast cancer cell lines specified were used for immunoblots for Erk5 which was upregulated in cluster ii (and to some extent in cluster i) patient subgroup in the Kinex^{TM} arrays. Erk5 was upregulated in TNBC cell lines compared to normal and non-TNBC cell lines. In contrast, RSK4 was downregulated in TNBC cell lines compared to other cells. RSK4 was downregulated in cluster ii patient subgroup in the Kinex^{TM} arrays. Cells were cultured in reduced serum condition (2 % FCS) for 24 hours to investigate basal (resting) levels. g-tubulin and b-actin were used as loading control.

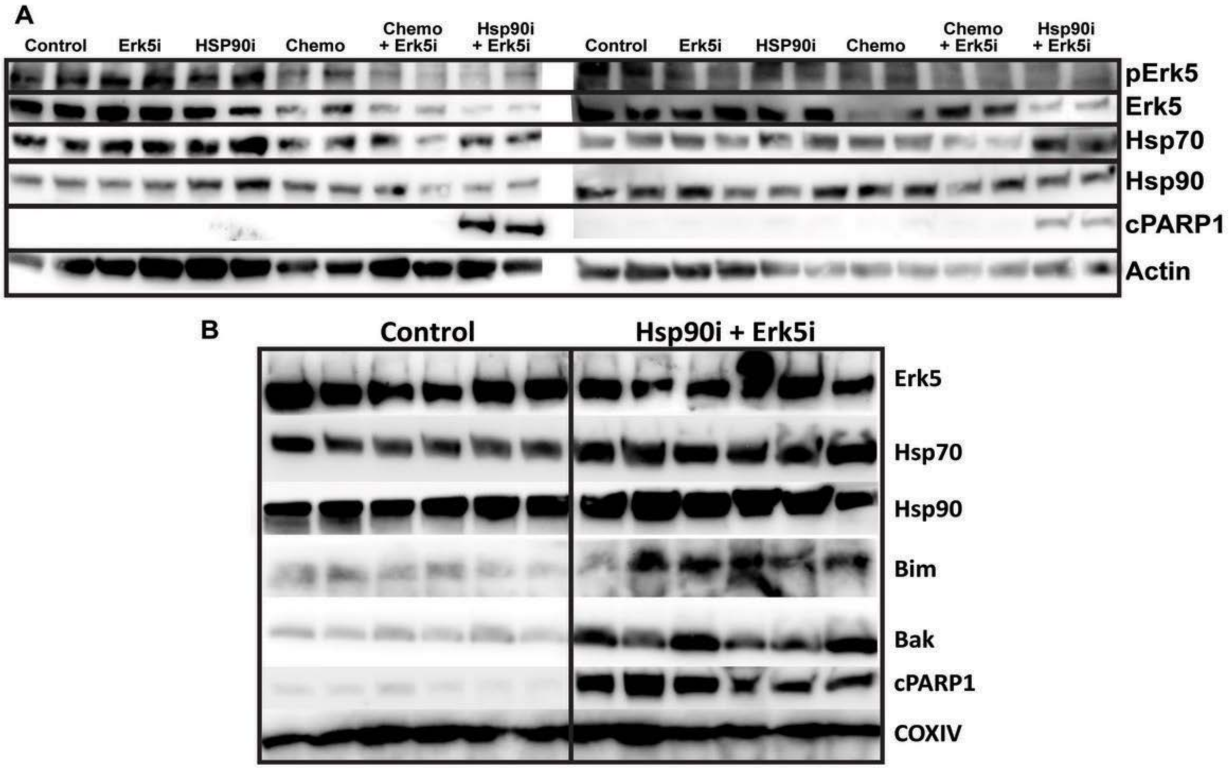

Supplementary Figure 7: Combined inhibition of Hsp90 and Erk5 induces apoptotic cell death *in vivo*. Female balb/c nude mice bearing 50mm^3 xenografts from the TNBC MDA-MB-231 cell line were treated as described in Fig.6 in the article and 6 tumors were excised on day 13 (after treatment completion) for immunoblot analysis. (**A**) Immunoblots from the remaining four tumors per group (2 tumors per treatment group per membrane) for the detection of total Erk5, phosphorylated Erk5 (pErk5), Hsp70 and cleaved PARP1 (cPARP1). (**B**) Immunoblots for Erk5, Hsp70, Hsp90, Bim, Bak and cPARP1 for the 6 control tumors and 6 tumors treated with the Hsp90i+Erk5i combination.
