# Supplementary material for: Kinome profiling reveals breast cancer heterogeneity and identifies targeted therapeutic opportunities for triple negative breast cancer
Source: Oncotarget. 2014 Mar 26;5(10):3145–58. doi: 10.18632/oncotarget.1865 (PMC4102798; doi:10.18632/oncotarget.1865)
Supplement: Supplementary file 2 [file oncotarget-05-3145-s002.pdf]

Supplementary Table 1: Primary breast tumors for Kinex™ antibody arrays

Triple negative (Medians. Age: 57 years; Size: 30 mm; Grade: 3; Tumor content: 60%)

ID	Age	Subtype	Size (mm)	ER	PR	HER2	Grade	Tumor content	Technical replicates Pearson's correlation (R^2)
**Q075**	57	TNBC	43	Neg	Neg	Neg	3	60%	0.929
**Q126**	49	TNBC	15	Neg	Neg	Neg	2	70%	0.913
**Q151**	67	TNBC	30	Neg	Neg	Neg	3	50%	0.939
**Q184**	69	TNBC	38	Neg	Neg	Neg	2	60%	0.899
**Q218**	62	TNBC	30	Neg	Neg	Neg	3	50%	0.885
**Q230**	45	TNBC	15	Neg	Neg	Neg	3	40%	0.902
**Q234**	85	TNBC	12	Neg	Neg	Neg	3	60%	0.852
**Q268**	58	TNBC	21	Neg	Neg	Neg	3	70%	0.927
**Q279**	47	TNBC	40	Neg	Neg	Neg	3	80%	0.887
Q281	88	TNBC	45	Neg	Neg	Neg	3	90%	0.889
Q312	54	TNBC	19	Neg	Neg	Neg	3	70%	0.883
Q351	43	TNBC	18	Neg	Neg	Neg	2	50%	0.884
Q407	39	TNBC	30	Neg	Neg	Neg	3	80%	0.935
Q437	70	TNBC	17	Neg	Neg	Neg	3	50%	0.903
Q543	49	TNBC	40	Neg	Neg	Neg	3	70%	0.928

ER/PR positive (Medians. Age: 52 years;Size: 26mm; Grade: 3; Tumor content: 60%)

ID	Age	Subtype	Size (mm)	ER	PR	HER2	Grade	Tumor content	Technical replicates Pearson's correlation (R^2)
Q093	79	ER/PR+	45	Pos	Pos	Neg	2	50%	0.920
Q129	54	ER/PR+	22	Pos	Pos	Neg	3	70%	0.858
Q132	73	ER/PR+	17	Pos	Pos	Neg	3	70%	0.924
Q145	49	ER/PR+	20	Pos	Pos	Neg	3	60%	0.910
Q240	47	ER/PR+	50	Pos	Pos	Neg	2	50%	0.852
Q248	63	ER/PR+	16	Pos	Pos	Neg	2	50%	0.900
Q254	60	ER/PR+	30	Pos	Pos	Neg	2	70%	0.893
Q356	90	ER/PR+	60	Pos	Pos	Neg	3	50%	0.904
Q358	45	ER/PR+	18	Pos	Pos	Neg	3	70%	0.902
Q359	45	ER/PR+	45	Pos	Pos	Neg	3	60%	0.942
Q377	49	ER/PR+	18	Pos	Pos	Neg	3	40%	0.920
Q382	59	ER/PR+	21	Pos	Pos	Neg	3	60%	0.936
Q411	38	ER/PR+	50	Pos	Pos	Neg	2	80%	0.932
Q450	50	ER/PR+	15	Pos	Pos	Neg	3	80%	0.909
Q488	44	ER/PR+	75	Pos	Pos	Neg	3	50%	0.894

HER2 positive (Medians. Age: 51 years;Size: 28mm; Grade: 3; Tumor content: 60%)

ID	Age	Subtype	Size (mm)	ER	PR	HER2	Grade	Tumor content	Technical replicates Pearson's correlation (R2)
Q173	50	HER2+	28	Neg	Neg	Pos	3	60%	0.885
Q203	54	HER2+	12	Neg	Neg	Pos	2	50%	0.904
Q271	86	HER2+	45	Neg	Neg	Pos	3	60%	0.901
Q398	42	HER2+	40	Neg	Neg	Pos	2	80%	0.895
Q213	30	HER2+	20	Pos	Neg	Pos	3	70%	0.868
Q328	61	HER2+	28	Pos	Neg	Pos	3	50%	0.873
Q087	52	HER2+	28	Pos	Pos	Pos	2	50%	0.924
Q179	37	HER2+	20	Pos	Pos	Pos	3	50%	0.931
Q340	57	HER2+	15	Pos	Pos	Pos	3	80%	0.916
Q457	51	HER2+	18	Pos	Pos	Pos	3	50%	0.915
Q489	46	HER2+	160	Pos	Pos	Pos	3	60%	0.902

Supplementary Table 2: Differential analysis between all primary tumor clusters

	Probes							i		ii		iii		iv		v		vi		Activity Scores						Cluster
	UniqueID	Name	CloneID	Symbol	Uniprot	Official name	Gene names	Mean	Stdev	Mean	Stdev	Mean	Stdev	Mean	Stdev	Mean	Stdev	Mean	Stdev	ii	i	vi	iv	iii	v	Branch
	NP023-2	Phosphatidyli	Pan-specific	PTEN	P60484	PTEN_HUMAN	PTEN MMAC1	2296	1	795	3	2300	1	1862	1	2056	1	2544	1	0.31	0.90	1.00	0.73	0.90	0.81	1
1	PK103	Ribosomal S6	T359+S363/T356+S36(	RSK1/3	Q15418	KS6A1_HUMA	RPS6KA1 MAP	1263	1	1135	1	1147	1	375	1	1030	2	1881	1	0.60	0.67	1.00	0.20	0.61	0.55	
	PK074	Protein-serine	T638/T641	PKCa/b2	P17252	KPCA_HUMAN	PRKCA PKCA P	835	1	2062	1	2476	1	2258	1	2214	2	1550	2	0.83	0.34	0.63	0.91	1.00	0.89	2
	NK082	Interleukin 1 r	Pan-specific	IRAK3	Q9Y616	IRAK3_HUMA	IRAK3	2023	1	4944	1	4743	2	5708	1	5880	1	2998	2	0.84	0.34	0.51	0.97	0.81	1.00	
	PK084	Protein-serine	T674	PKCg	P05129	KPCG_HUMAN	PRKCG PKCG	5502	1	5405	1	14072	1	13214	1	13708	1	12198	1	0.38	0.39	0.87	0.94	1.00	0.97	
	NN069	DNA mismatcl	Pan-specific	MSH2	P43246	MSH2_HUMA	MSH2	8366	1	4455	1	9802	1	10926	1	11395	1	9102	1	0.39	0.73	0.80	0.96	0.86	1.00	
	PK073	Protein-serine	S657	PKCa	P17252	KPCA_HUMAN	PRKCA PKCA P	8760	1	2665	1	10274	1	11661	1	11913	1	9928	1	0.22	0.74	0.83	0.98	0.86	1.00	
	NN077	p18 INK4c cyc	Pan-specific	p18 INK4c	P42773	CDN2C_HUM/	CDKN2C CDKN	8530	1	8590	1	15301	1	15743	1	16452	1	13088	1	0.52	0.52	0.80	0.96	0.93	1.00	
	PK025	G protein-cou	S670	GRK2 (BARK1)	P25098	ARBK1_HUMA	ADRBK1 BARK	8171	1	6671	1	12046	1	13178	1	13149	1	9530	1	0.51	0.62	0.72	1.00	0.91	1.00	
	NK042	Osaka thyroid	Pan-specific	COT	P41279	M3K8_HUMA	MAP3K8 COT	1788	1	1573	1	2507	1	2158	1	2911	1	1606	1	0.54	0.61	0.55	0.74	0.86	1.00	
	NK028-4	Cyclin-depend	Pan-specific	CDK5	Q00535	CDK5_HUMAN	CDK5 CDKN5	3638	1	3026	1	4178	1	4565	1	6121	1	2865	1	0.49	0.59	0.47	0.75	0.68	1.00	
	PK119	Checkpoint pr	T68	Chk2	O96017	CHK2_HUMAN	CHEK2 CDS1 C	4229	1	2998	2	5248	1	5714	1	6221	1	4309	1	0.48	0.68	0.69	0.92	0.84	1.00	
	NK008-2	Aurora Kinase	Pan-specific	Aurora A (AIK)	O14965	AURKA_HUM/	AURKA AIK AIF	4120	1	788	2	4552	1	4273	1	5202	1	3796	1	0.15	0.79	0.73	0.82	0.88	1.00	
	NK076-1	Inhibitor of NI	Pan-specific	IKKb	O14920	IKKB_HUMAN	IKBKB IKKB	6763	1	2627	1	7223	1	6274	1	8595	1	6148	1	0.31	0.79	0.72	0.73	0.84	1.00	
	NN032	Cyclin G1	Pan-specific	Cyclin G1	P51959	CCNG1_HUM/	CCNG1 CCNG	5933	1	4547	2	10299	1	10331	1	11196	1	7781	1	0.41	0.53	0.69	0.92	0.92	1.00	
	NK001	Abelson proto	Pan-specific	Abl	P00519	ABL1_HUMAN	ABL1 ABL JTK7	3416	1	2008	2	5797	1	5568	1	6842	1	4529	1	0.29	0.50	0.66	0.81	0.85	1.00	
	NK013	Bromodomain	Pan-specific	BRD2	P25440	BRD2_HUMAI	BRD2 KIAA900	4211	1	3474	1	6195	1	6307	1	7422	1	6323	1	0.47	0.57	0.85	0.85	0.83	1.00	
	PN010	Bcl2-antagoni	S75	Bad	Q92934	BAD_HUMAN	BAD BBC6 BCL	9512	1	7607	1	9231	1	11035	1	13623	1	8832	1	0.56	0.70	0.65	0.81	0.68	1.00	
	NK071	Haploid germ	Pan-specific	Haspin	Q8TF76	HASP_HUMAN	GSG2	5658	1	3015	1	4156	1	4203	1	5366	1	3249	1	0.53	1.00	0.57	0.74	0.73	0.95	
	NK075-5	Inhibitor of NI	Pan-specific	IKKa	O15111	IKKA_HUMAN	CHUK IKKA TCI	5786	1	2702	1	4199	1	4670	1	5452	1	3766	1	0.47	1.00	0.65	0.81	0.73	0.94	
	NK181-2	Protein-tyrosi	Pan-specific	Tyk2	P29597	TYK2_HUMAN	TYK2	3290	1	3830	1	4022	1	3625	1	5003	1	2623	1	0.77	0.66	0.52	0.72	0.80	1.00	
	NN156	1-phosphatidy	Pan-specific	PLC R(PLCg2)	P16885	PLCG2_HUMA	PLCG2	2498	1	2939	1	4187	1	3761	1	5490	1	2832	1	0.54	0.46	0.52	0.69	0.76	1.00	
	PK071-2	Protein-serine	T308	PKBa (Akt1)	P31749	AKT1_HUMAN	AKT1 PKB RAC	389	1	1560	1	3096	1	2912	1	3304	1	2211	1	0.47	0.12	0.67	0.88	0.94	1.00	
	NK198	Casein kinase	Pan-specific	CK1g	Q8IXA3	Q8IXA3_HUM	hCG_2004507	1643	1	1696	1	2584	1	2397	1	2549	1	1363	1	0.66	0.64	0.53	0.93	1.00	0.99	
	NK187-2	Zeta-chain (TC	Pan-specific	ZAP70	P43403	ZAP70_HUMA	ZAP70 SRK	2697	1	5460	1	8863	1	6971	1	7615	1	4387	1	0.62	0.30	0.49	0.79	1.00	0.86	
	NK201	Protein-serine	Pan-specific	PKC	P17252	KPCA_HUMAN	PRKCA PKCA P	1229	1	1744	1	2286	1	1802	1	2350	1	1335	1	0.74	0.52	0.57	0.77	0.97	1.00	
	PN015	Caldesmon	S789	Caldesmon	Q05682	CALD1_HUMA	CALD1 CAD CD	7099	1	2238	3	5629	1	4785	1	3714	1	6531	1	0.32	1.00	0.92	0.67	0.79	0.52	3
	NK162	ROR2 neurotr	Pan-specific	ROR2	Q01974	ROR2_HUMAI	ROR2 NTRKR2	3441	1	3915	1	5121	1	5894	1	3424	1	6300	1	0.62	0.55	1.00	0.94	0.81	0.54	
	NK199	Cyclin-depend	Pan-specific	CDKL1	Q00532	CDKL1_HUMA	CDKL1	2631	1	1217	2	3637	1	3757	1	2324	1	4151	2	0.29	0.63	1.00	0.91	0.88	0.56	
	NK118	NIMA (never-i	Pan-specific	Nek4	P51957	NEK4_HUMAN	NEK4 STK2	2391	1	2885	1	5248	1	4103	1	3101	1	4050	1	0.55	0.46	0.77	0.78	1.00	0.59	
	NK192	phosphatidyli	Pan-specific	PI4KCB	Q9UBF8	PI4KB_HUMAI	PI4KB PIK4CB	3451	1	3837	1	6734	1	6117	1	4203	1	7453	1	0.51	0.46	1.00	0.82	0.90	0.56	
	NP022-2	Protein-serine	Pan-specific	PP6C	O00743	PPP6_HUMAN	PPP6C PPP6	2738	1	1002	1	4382	1	3419	1	3003	1	4190	1	0.23	0.62	0.96	0.78	1.00	0.69	
	NK104	MAPK/ERK pr	Pan-specific	MEK5 (MAP2K5)	Q13163	MP2K5_HUM/	MAP2K5 MEK5	4801	1	7544	2	5797	1	6845	1	5599	1	1879	1	1.00	0.64	0.25	0.91	0.77	0.74	
	PK095-PK096	Protein kinase	T774, T816	PRK1/2 (PKN1/2)	Q16512	PKN1_HUMAN	PKN1 PAK1 PK	7738	1	3564	1	10018	1	10591	1	8601	1	9720	1	0.34	0.73	0.92	1.00	0.95	0.81	
	NK011	B lymphoid ty	Pan-specific	BLK	P51451	BLK_HUMAN	BLK	3247	1	3840	2	8201	1	7274	1	8608	1	7452	1	0.45	0.38	0.87	0.84	0.95	1.00	
	NN015	Caspase 5 (ICI	Pan-specific	CASP5	P51878	CASP5_HUMA	CASP5 ICH3	4292	1	3103	1	8244	1	7127	1	7724	1	8073	1	0.38	0.52	0.98	0.86	1.00	0.94	
	NK219	Diacylglycerol	Pan-specific	DGKz	Q13574	DGKZ_HUMAI	DGKZ DAGK6	5675	1	3590	1	7101	1	6237	1	7366	1	7636	1	0.47	0.74	1.00	0.82	0.93	0.96	
	NK189	Jun N-terminu	Pan-specific	JNK2	P45984	MK09_HUMAI	MAPK9 JNK2 P	6915	1	4477	1	7551	1	7597	1	6839	1	8135	1	0.55	0.85	1.00	0.93	0.93	0.84	
	NN034	Death-associa	Pan-specific	DAXX	Q9UER7	DAXX_HUMAI	DAXX BING2 D	6604	1	3823	1	11281	1	12091	1	10827	1	11825	1	0.32	0.55	0.98	1.00	0.93	0.90	
	NK080	Interleukin 1 r	Pan-specific	IRAK1	P51617	IRAK1_HUMA	IRAK1 IRAK	6464	1	5177	1	9382	1	9047	1	8701	1	9024	1	0.55	0.69	0.96	0.96	1.00	0.93	
	NK053	Ephrin type-A	Pan-specific	EphA1	P21709	EPHA1_HUMA	EPHA1 EPH EP	5169	1	3811	1	7781	1	8017	1	5603	1	8655	1	0.44	0.60	1.00	0.93	0.90	0.65	
	NK078-2	Integrin-linke	Pan-specific	ILK1	Q13418	ILK_HUMAN	ILK ILK1 ILK2	4971	1	3308	1	8846	1	8370	1	7415	1	9491	1	0.35	0.52	1.00	0.88	0.93	0.78	
	NK006	Ankyrin repea	Pan-specific	ANKRD3	P57078	RIPK4_HUMAI	RIPK4 ANKRD3	6071	1	4418	1	5507	1	5801	1	5031	1	8229	1	0.54	0.74	1.00	0.70	0.67	0.61	
	NK008-1	Aurora Kinase	Pan-specific	Aurora A (AIK)	O14965	AURKA_HUM/	AURKA AIK AIF	4135	1	1272	2	4333	1	4618	1	3610	1	5375	1	0.24	0.77	1.00	0.86	0.81	0.67	
	NK183-2	Neurotrophic	Pan-specific	Tyro10 (DDR2)	Q16832	DDR2_HUMAI	DDR2 NTRKR3	5411	1	3393	1	8100	1	5531	1	6449	1	9359	1	0.36	0.58	1.00	0.59	0.87	0.69	
	PN131	Retinoblastom	S795	Rb	P06400	RB_HUMAN	RB1	7769	1	547	3	7646	1	6398	1	6388	1	10320	1	0.05	0.75	1.00	0.62	0.74	0.62	
	NK064	Vascular endo	Pan-specific	FLT4	P35916	VGFR3_HUMA	FLT4 VEGFR3	7425	1	7774	1	11198	1	11281	1	11290	1	14070	1	0.55	0.53	1.00	0.80	0.80	0.80	
	NK012	Bone marrow	Pan-specific	BMX (Etk)	P51813	BMX_HUMAN	BMX	6882	1	3601	1	8183	1	7724	1	8076	1	10792	1	0.33	0.64	1.00	0.72	0.76	0.75	
	NK019-1	Calcium/calm	Pan-specific	CAMK2d	Q13557	KCC2D_HUMA	CAMK2D CAM	8781	1	6646	1	10804	1	11708	1	12412	1	13998	1	0.47	0.63	1.00	0.84	0.77	0.89	
	NK042-2	Osaka thyroid	Pan-specific	COT	P41279	M3K8_HUMA	MAP3K8 COT	6584	1	2590	2	9859	1	10475	1	10562	1	11744	1	0.22	0.56	1.00	0.89	0.84	0.90	
	NN058	Heat shock 47	Pan-specific	Hsp47	P50454	SERPH_HUMA	SERPINH1 CBP	4131	1	4887	1	8611	1	8756	1	7008	1	9797	1	0.50	0.42	1.00	0.89	0.88	0.72	
	NK062	Fibroblast gro	Pan-specific	FGFR1	P11362	FGFR1_HUMA	FGFR1 BFGFR	7222	1	5532	1	11921	1	10071	1	11707	1	15188	1	0.36	0.48	1.00	0.66	0.78	0.77	
	NK191	Phosphatidyli	Pan-specific	PI3K p110 delta	O00329	PK3CD_HUMA	PIK3CD	4293	1	448	2	6027	1	5302	1	5916	1	7593	1	0.06	0.57	1.00	0.70	0.79	0.78	
	NK063	Fibroblast gro	Pan-specific	FGFR2	P21802	FGFR2_HUMA	FGFR2 BEK KG	4975	1	2957	1	9372	1	8644	1	8557	1	10987	1	0.27	0.45	1.00	0.79	0.85	0.78	
	NK121	Mitogen-activ	Pan-specific	p38d MAPK	O15264	MK13_HUMAI	MAPK13 PRKM	7838	1	6165	1	11281	1	10258	1	10804	1	13338	1	0.46	0.59	1.00	0.77	0.85	0.81	
	NP010	Protein-serine	Pan-specific	PP1/Cb	P62140	PP1B_HUMAN	PPP1CB	4230	1	3291	1	4290	1	4806	1	4672	1	7060	1	0.47	0.60	1.00	0.68	0.61	0.66	
	NK073	Intestinal cell	Pan-specific	ICK	Q9UPZ9	ICK_HUMAN	ICK KIAA0936	3717	1	3175	1	4779	1	5091	1	4623	1	6037	1	0.53	0.62	1.00	0.84	0.79	0.77	
	NK173	FLJ35932 prot	Pan-specific	STK33	Q9BYT3	STK33_HUMA	STK33	5262	1	1704	1	5989	1	6144	1	6227	1	9622	1	0.18	0.55	1.00	0.64	0.62	0.65	
	NN160	Activating trar	Pan-specific	ATF2	P15336	ATF2_HUMAN	ATF2 CREB2 CI	4467	1	1513	2	5330	1	5218	1	4890	1	8480	1	0.18	0.53	1.00	0.62	0.63	0.58	
	NP034	Phosphatidyli	Pan-specific	TPIPb	Q6XPS3	TPTE2_HUMA	TPTE2 TPIP	3284	1	624	1	3226	1	2541	1	2891	1	4306	1	0.14	0.76	1.00	0.59	0.75	0.67	
	NK171	STE20-like pro	Pan-specific	SLK	Q9H2G2	SLK_HUMAN	SLK KIAA0204	4495	1	4683	1	10098	1	6977	1	8917	1	11242	1	0.42	0.40	1.00	0.62	0.90	0.79	
	NN101	Sphingosine k	Pan-specific	SPHK2	Q9NRA0	SPHK2_HUMA	SPHK2	7194	1	4282	1	8322	1	7587	1	7901	1	10402	1	0.41	0.69	1.00	0.73	0.80	0.76	
	NK209	Phosphatidyli	Pan-specific	PIP5K2a	P48426	PI42A_HUMAI	PIP4K2A PIP5K	4226	1	4284	1	6005	1	4948	1	5525	1	8272	1	0.52	0.51	1.00	0.60	0.73	0.67	
	NN114	Phosphoinosit	Pan-specific	PI3KR4	Q99570	PI3R4_HUMAI	PIK3R4	3634	1	3457	1	5345	1	5256	1	4371	1	6827	1	0.51	0.53	1.00	0.77	0.78	0.64	
	PK013-1	ErbB2 (Neu) r	Y1248	ErbB2 (HER2)	P04626	ERBB2_HUMA	ERBB2 HER2 M	7132	1	2357	1	7318	1	5330	1	8047	1	5750	1	0.29	0.89	0.71	0.66	0.91	1.00	4
	NP010-2	Protein-serine	Pan-specific	PP1/Cb	P62140	PP1B_HUMAN	PPP1CB	7299	1	3993	1	11423	1	9255	1	10468	1	8121	1	0.35	0.64	0.71	0.81	1.00	0.92	
	PN026	Disabled hom	Y198	Dab1	O75553	DAB1_HUMAI	DAB1	4876	2	1262	2	7514	1	6847	1	8121	1	5337	1	0.16	0.60	0.66	0.84	0.93	1.00	
	NK174	Spleen proteir	Pan-specific	Syk	P43405	KSYK_HUMAN	SYK	6142	1	3727	1	9954	1	7828	1	9381	1	8007	1	0.37	0.62	0.80	0.79	1.00	0.94	
	PK113	Zeta-chain (TC	Y315+Y319	ZAP70	P43403	ZAP70_HUMA	ZAP70 SRK	4829	1	795	2	7759	1	5715	1	7208	1	6753	1	0.10	0.62	0.87	0.74	1.00	0.93	
	NN097	Suppressor of	Pan-specific	SOCS4	Q8WXH5	SOCS4_HUMA	SOCS4 SOCS7	6676	1	5420	1	9585	1	6842	1	9593	1	9044	1	0.57	0.70	0.94	0.71	1.00	1.00	
	NP035	Protein-serine	Pan-specific	PP2A/Bb	Q00005	2ABB_HUMAN	PPP2R2B	6734	1	4864	1	8071	1	7495	1	8086	1	8593	1	0.57	0.78	1.00	0.87	0.94	0.94	
	PN064	Rad17 homolo	S645	Rad17	O75943	RAD17_HUMA	RAD17 R24L	4096	1	2847	1	8259	1	7351	1	7780	1	6703	1	0.34	0.50	0.81	0.89	1.00	0.94	
	NK152	Protein-serine	Pan-specific	PRP4K	Q13523	PRP4B_HUMA	PRPF4B KIAA0	3886	1	3812	1	8648	1	6818	1	7914	1	7012	1	0.44	0.45	0.81	0.79	1.00	0.92	
	NP019	Protein-serine	Pan-specific	PP4/Aâ€™2	Q8TF05	PP4R1_HUMA	PPP4R1 MEG1	6323	1	4699	1	9609	1	8310	1	9451	1	8848	1	0.49	0.66	0.92	0.86	1.00	0.98	
	NK149	Protein kinase	Pan-specific	PRK2 (PKN2)	Q16513	PKN2_HUMAN	PKN2 PRK2 PR	7895	1	6317	1	12452	1	9907	1	11564	1	12097	1	0.51	0.63	0.97	0.80	1.00	0.93	
	NK163	Orosomucoid	Pan-specific	ROS	P08922	ROS1_HUMAN	ROS1 MCF3 R	4560	1	5359	1	10209	1	6532	1	8676	1	8629	1	0.52	0.45	0.85	0.64	1.00	0.85	
	NK151	Putative prote	Pan-specific	PRKWNK4	Q96J92	WNK4_HUMA	WNK4 PRKWN	5203	1	4910	1	9862	1	7658	1	8123	1	8484	1	0.50	0.53	0.86	0.78	1.00	0.82	
	NK166	Ribosomal S6	Pan-specific	RSK4	Q9UK32	KS6A6_HUMA	RPS6KA6 RSK4	5114	1	3696	1	11094	1	7983	1	6941	1	10609	1	0.33	0.46	0.96	0.72	1.00	0.63	
	NK066	Germinal cent	Pan-specific	GCK	Q12851	M4K2_HUMAI	MAP4K2 GCK	6414	1	4741	1	6803	1	6478	1	8537	1	7657	1	0.56	0.75	0.90	0.76	0.80	1.00	
	NP004	Cyclin-depend	Pan-specific	KAP	Q16667	CDKN3_HUM/	CDKN3 CDI1 C	2541	1	3451	1	5587	1	4459	1	6296	1	5793	1	0.55	0.40	0.92	0.71	0.89	1.00	
	PN055	N-methyl-D-a	S896	NR1	Q05586	NMDZ1_HUM	GRIN1 NMDAF	7495	1	1742	2	6347	1	5662	1	7114	1	6875	1	0.23	1.00	0.92	0.76	0.85	0.95	
2	PN078-PN135	Signal transdu	S727	STAT1a/b	P42224	STAT1_HUMA	STAT1	6603	1	2586	1	7532	1	7034	1	9024	1	8484	1	0.29	0.73	0.94	0.78	0.83	1.00	
1	PN079-PN136	Signal transdu	Y701	STAT1a/b	P42224	STAT1_HUMA	STAT1	971	1	1953	1	2284	1	1300	1	3699	1	4519	1	0.43	0.21	1.00	0.29	0.51	0.82	
	NP022	Protein-serine	Pan-specific	PP6C	O00743	PPP6_HUMAN	PPP6C PPP6	5479	1	4682	1	9051	1	8251	1	10047	1	10118	1	0.46	0.54	1.00	0.82	0.89	0.99	
	PN071	Retinoblastom	T826	Rb	P06400	RB_HUMAN	RB1	9072	1	6003	1	10095	1	9560	1	10766	1	11659	1	0.51	0.78	1.00	0.82	0.87	0.92	
	PN051-1	Myristoylated	S158+S162	MARCKS	P29966	MARCS_HUM	MARCKS MAC	526	1	2696	2	6144	1	6422	1	6252	1	7178	1	0.38	0.07	1.00	0.89	0.86	0.87	
	NN085-2	Poly [ADP-ribc	Pan-specific	PARP1	P09874	PARP1_HUMA	PARP1 ADPRT	1783	1	1562	1	4117	1	4145	1	4912	1	4572	1	0.32	0.36	0.93	0.84	0.84	1.00	
	PN058	Paired box prc	S394	Pax2	Q02962	PAX2_HUMAN	PAX2	1011	1	2343	2	4952	1	6703	1	7107	1	5934	1	0.33	0.14	0.83	0.94	0.70	1.00	
	NN081-NN120	CDK5 regulato	Pan-specific	p35, p25	Q15078	CD5R1_HUM/	CDK5R1 CDK5	4349	1	1623	1	2636	1	2090	1	2518	1	2476	1	0.37	1.00	0.57	0.48	0.61	0.58	5
	NK032	Cyclin-depend	Pan-specific	CDK9	P50750	CDK9_HUMAN	CDK9 CDC2L4	3771	1	1808	1	1809	1	1577	1	2513	1	2370	1	0.48	1.00	0.63	0.42	0.48	0.67	
	PK013-2	ErbB2 (Neu) r	Y1248	ErbB2 (HER2)	P04626	ERBB2_HUMA	ERBB2 HER2 M	3875	1	1695	1	2389	1	2572	1	3197	1	3210	1	0.44	1.00	0.83	0.66	0.62	0.83	
	NK050	DAP kinase-re	Pan-specific	DRAK2	O94768	ST17B_HUMA	STK17B DRAK2	1844	1	674	2	1940	1	2361	1	2257	1	2019	1	0.29	0.78	0.86	1.00	0.82	0.96	
	NN141-1	Protein disulfi	Pan-specific	PDI	P07237	PDIA1_HUMA	P4HB ERBA2L	9385	1	4955	1	9275	1	9715	1	10488	1	9409	1	0.47	0.89	0.90	0.93	0.88	1.00	
	PK088	Protein-serine	T538	PKCq	Q04759	KPCT_HUMAN	PRKCQ PRKCT	8661	1	2361	1	8406	1	9176	1	7403	1	8511	1	0.26	0.94	0.93	1.00	0.92	0.81	
	NK148	Protein kinase	Pan-specific	PRK1 (PKN1)	Q16512	PKN1_HUMAN	PKN1 PAK1 PK	8364	1	2066	1	6321	1	8732	1	7055	1	7718	1	0.24	0.96	0.88	1.00	0.72	0.81	
	PN049-PN112-	Mitogen-activ	T334	MAPKAPK2ab	P49137	MAPK2_HUM	MAPKAPK2	8720	1	2041	1	8707	1	10972	1	9654	1	9686	1	0.19	0.79	0.88	1.00	0.79	0.88	
	NK068	G protein-cou	Pan-specific	GRK3 (BARK2)	P35626	ARBK2_HUMA	ADRBK2 BARK	4520	2	2560	2	4914	1	6186	1	6354	1	6297	1	0.40	0.71	0.99	0.97	0.77	1.00	
	NK051	Elongation fac	Pan-specific	eEF2K	O00418	EF2K_HUMAN	EEF2K	9663	1	4983	1	6368	1	7615	1	8403	1	8098	1	0.52	1.00	0.84	0.79	0.66	0.87	
	NK083-1	Interleukin 1 r	Pan-specific	IRAK4	Q9NWZ3	IRAK4_HUMA	IRAK4	8616	1	4126	1	6795	1	8292	1	8791	1	9304	1	0.44	0.93	1.00	0.89	0.73	0.94	
	NK004	Amyotrophic l	Pan-specific	ALS2CR7 (PFTAIRE2)	Q96Q40	CDK15_HUMA	CDK15 ALS2CR	14997	1	7953	1	11101	1	12095	1	15305	1	13080	1	0.52	0.98	0.85	0.79	0.73	1.00	
	NN064-4	Inhibitor of NI	Pan-specific	IkBa	P25963	IKBA_HUMAN	NFKBIA IKBA M	8742	1	3306	1	7157	1	7137	1	8059	1	7745	1	0.38	1.00	0.89	0.82	0.82	0.92	
	NK029-3	Cyclin-depend	Pan-specific	CDK6	Q00534	CDK6_HUMAN	CDK6 CDKN6	9481	1	3913	1	7132	1	8346	1	7932	1	7400	1	0.41	1.00	0.78	0.88	0.75	0.84	
	NK214	Serine/threon	Pan-specific	YSK1	O00506	STK25_HUMA	STK25 SOK1 Y	11512	1	5854	1	7195	1	8762	1	8902	1	8262	1	0.51	1.00	0.72	0.76	0.63	0.77	
	NN017	Pro-caspase 7	Pan-specific	CASP7	P55210	CASP7_HUMA	CASP7 MCH3	8740	1	3155	1	7040	1	9120	1	7639	1	7888	1	0.35	0.96	0.86	1.00	0.77	0.84	
	NK127-1	cAMP-depend	Pan-specific	PKA Ca/b	P17612	KAPCA_HUMA	PRKACA PKAC	10414	1	8923	1	9304	1	9283	1	8915	1	9125	1	0.86	1.00	0.88	0.89	0.89	0.86	6
	PN093	Tyrosine hydr	S71	Tyrosine Hydroxylase	P07101	TY3H_HUMAN	TH TYH	2877	1	2256	1	549	5	1611	2	446	3	422	5	0.78	1.00	0.15	0.56	0.19	0.15	
	PK083	Protein-serine	T655	PKCg	P05129	KPCG_HUMAN	PRKCG PKCG	9503	1	11212	1	11880	2	14338	1	6629	2	10458	2	0.78	0.66	0.73	1.00	0.83	0.46	
	NK146	Polo-like prote	Pan-specific	Plk2	Q9NYY3	PLK2_HUMAN	PLK2 SNK	4334	1	5280	1	6247	1	6831	1	3327	1	5296	1	0.77	0.63	0.78	1.00	0.91	0.49	
	PK081-1	Protein-serine	S729	PKCe	Q02156	KPCE_HUMAN	PRKCE PKCE	4277	1	4828	1	4802	1	7027	1	2852	1	3366	1	0.69	0.61	0.48	1.00	0.68	0.41	
	NN142	Disks large ho	Pan-specific	PSD-95	P78352	DLG4_HUMAN	DLG4 PSD95	3190	1	2420	1	4232	2	3661	2	2184	1	2954	1	0.57	0.75	0.70	0.87	1.00	0.52	
	NN014	Pro-caspase 4	Pan-specific	CASP4	P49662	CASP4_HUMA	CASP4 ICH2	6352	1	4257	1	5743	2	6747	1	3366	1	6279	2	0.63	0.94	0.93	1.00	0.85	0.50	
	PN035	Histone H1 ph	phospho CDK1 sites	Histone H1	Q02539	H11_HUMAN	HIST1H1A H1F	5007	1	3250	1	3836	1	6080	1	2735	1	4230	1	0.53	0.82	0.70	1.00	0.63	0.45	
	NN016	Pro-caspase 6	Pan-specific	CASP6	P55212	CASP6_HUMA	CASP6 MCH2	3995	1	3028	1	5366	2	5881	2	4144	2	2292	3	0.51	0.68	0.39	1.00	0.91	0.70	
	NN007	Bcl2-like prote	Pan-specific	Bcl-xL	Q07817	B2CL1_HUMA	BCL2L1 BCL2L	20947	1	12891	1	19273	1	18396	1	8866	2	9341	2	0.62	1.00	0.45	0.88	0.92	0.42	
	NP032	Protein-serine	Pan-specific	PP2A/Bg2	Q9Y2T4	2ABG_HUMAI	PPP2R2C	6202	1	2505	1	5452	1	3246	2	4125	2	4133	1	0.40	1.00	0.67	0.52	0.88	0.67	
2	NK206-1	Extracellular r	Pan-specific	Erk5	Q13164	MK07_HUMAI	MAPK7 BMK1	5164	1	10877	2	4964	1	5627	1	5570	1	2668	1	1.00	0.47	0.25	0.52	0.46	0.51	
	NN020	Pro-caspase 1	Pan-specific	CASP12	O08736	CASPC_MOUS	Casp12	3885	2	3919	1	6691	1	5013	1	4610	2	2668	2	0.59	0.58	0.40	0.75	1.00	0.69	
	NK020	Calcium/calm	Pan-specific	CAMK2g	Q13555	KCC2G_HUMA	CAMK2G CAM	3642	1	958	1	3626	1	3823	1	2371	1	2538	1	0.25	0.95	0.66	1.00	0.95	0.62	
	NN074	Nucleotide di	Pan-specific	NME7	Q9Y5B8	NDK7_HUMAI	NME7	2076	1	901	1	3103	1	2896	1	1943	2	1915	1	0.29	0.67	0.62	0.93	1.00	0.63	
	NK040	Casein protein	Pan-specific	CK1g2	P78368	KC1G2_HUMA	CSNK1G2 CK1	3283	1	2258	1	6089	1	5705	1	3983	1	3213	1	0.37	0.54	0.53	0.94	1.00	0.65	
	NN085-1	Poly [ADP-ribc	Pan-specific	PARP1	P09874	PARP1_HUMA	PARP1 ADPRT	5369	1	3088	1	8465	1	8918	1	6278	1	5852	1	0.35	0.60	0.66	1.00	0.95	0.70	
	NK037	Casein protein	Pan-specific	CK1e	P49674	KC1E_HUMAN	CSNK1E	2814	1	6506	1	6184	1	7006	1	4915	1	4532	1	0.93	0.40	0.65	1.00	0.88	0.70	
	PK069	cAMP-depend	S98	PKA R2a	P13861	KAP2_HUMAN	PRKAR2A PKR2	5368	1	6493	1	7197	1	6867	1	6032	1	3572	1	0.90	0.75	0.50	0.95	1.00	0.84	
	PN052	Myosin phosp	T696	MYPT1	O14974	MYPT1_HUM/	PPP1R12A MB	6841	1	4290	1	7270	1	7559	1	5923	1	3912	1	0.57	0.91	0.52	1.00	0.96	0.78	
	NN127	FK506-binding	Pan-specific	FKBP52	Q02790	FKBP4_HUMA	FKBP4 FKBP52	2362	1	2078	1	3945	2	3409	1	1982	1	2163	1	0.53	0.60	0.55	0.86	1.00	0.50	
	PK004	Bruton's agarr	Y223	Btk	Q06187	BTK_HUMAN	BTK AGMX1 A	1625	1	1530	1	1731	2	1719	1	995	1	946	1	0.88	0.94	0.55	0.99	1.00	0.57	
	PN014	Breast cancer	S1497	BRCA1	P38398	BRCA1_HUMA	BRCA1 RNF53	6093	1	4513	1	5535	1	6971	1	3888	1	3524	1	0.65	0.87	0.51	1.00	0.79	0.56	
	NK014	Bruton's agarr	Pan-specific	Btk	Q06187	BTK_HUMAN	BTK AGMX1 A	1943	1	3782	1	3333	1	3425	1	4433	1	3857	1	0.85	0.44	0.87	0.77	0.75	1.00	7
	PK061	p21-activated	S144/S141/S154	PAK1/2/3	Q13153	PAK1_HUMAN	PAK1	1267	1	2113	1	1916	1	1259	1	2365	1	2322	1	0.89	0.54	0.98	0.53	0.81	1.00	
1	NN105	Signal transdu	Pan-specific	STAT5A	P42229	STA5A_HUMA	STAT5A STATS	297	1	2357	3	2089	1	2318	1	2807	1	2277	1	0.84	0.11	0.81	0.83	0.74	1.00	
	PN125	Structural mai	S957	SMC1	Q14683	SMC1A_HUM	SMC1A DXS42	777	1	2161	2	1103	1	1079	1	1147	1	1106	1	1.00	0.36	0.51	0.50	0.51	0.53	
	NN068	Manganese su	Pan-specific	Mn SOD	P04179	SODM_HUMA	SOD2	3596	1	3544	1	4080	2	2147	1	3366	1	2222	2	0.87	0.88	0.54	0.53	1.00	0.83	
3	PK102	Ribosomal S6	T573/T577/T570	RSK1/2/3	Q15418	KS6A1_HUMA	RPS6KA1 MAP	4084	1	8256	1	8823	1	6991	1	9355	1	8924	1	0.88	0.44	0.95	0.75	0.94	1.00	
	PN127	Phosphatidyli	Y467/Y199	PI3K p85/p55	P27986	P85A_HUMAN	PIK3R1 GRB1	472	1	775	1	600	1	424	1	633	1	511	1	1.00	0.61	0.66	0.55	0.77	0.82	
	NK167	RYK tyrosine-p	Pan-specific	RYK	P34925	RYK_HUMAN	RYK JTK5A	3602	1	5446	1	7302	1	2697	1	3151	2	4180	2	0.75	0.49	0.57	0.37	1.00	0.43	
3	PN083	Signal transdu	Y694	STAT5A	P42229	STA5A_HUMA	STAT5A STATS	2126	1	3849	1	4059	1	2886	1	3664	1	2493	1	0.95	0.52	0.61	0.71	1.00	0.90	
	NK156	RafB proto-on	Pan-specific	RafB (Braf)	P15056	BRAF_HUMAN	BRAF BRAF1 R	5897	1	3583	1	3596	1	3887	1	4346	1	2295	1	0.61	1.00	0.39	0.66	0.61	0.74	
	PN044	Integrin beta	S785	Integrin b1	P05556	ITB1_HUMAN	ITGB1 FNRB M	3748	1	9065	1	8678	2	4973	2	3705	1	8506	1	1.00	0.41	0.94	0.55	0.96	0.41	
	PN103	Huntington's c	S421	Huntingtin	P42858	HD_HUMAN	HTT HD IT15	1171	1	3551	1	3306	1	2041	1	2851	2	3807	1	0.93	0.31	1.00	0.54	0.87	0.75	
	PK133	Vascular endo	Y1214	VEGFR2 (KDR)	P35968	VGFR2_HUMA	KDR FLK1 VEG	2039	1	6221	1	4336	1	5171	1	4025	2	5463	1	1.00	0.33	0.88	0.83	0.70	0.65	
4	PN081-2	Signal transdu	S727	STAT3	P40763	STAT3_HUMA	STAT3 APRF	4004	1	7937	1	5408	1	4051	1	4865	1	6320	1	1.00	0.50	0.80	0.51	0.68	0.61	
2	NK069-NK070	Glycogen synt	Pan-specific	GSK3ab	P49840	GSK3A_HUMA	GSK3A	4036	1	7236	1	4187	1	4448	1	4615	1	4646	1	1.00	0.56	0.64	0.61	0.58	0.64	
	PN008	B23 (nucleoph	T199	B23 (NPM)	P06748	NPM_HUMAN	NPM1 NPM	1605	1	5343	1	2290	1	1840	2	1668	2	2411	2	1.00	0.30	0.45	0.34	0.43	0.31	
	PN022-2	Cortactin (am	Y466	Cortactin	Q60598	SRC8_MOUSE	Cttn Ems1	324	4	2423	1	461	1	713	2	511	2	493	1	1.00	0.13	0.20	0.29	0.19	0.21	
	NK122-4	p21-activated	Pan-specific	PAK1	Q13153	PAK1_HUMAN	PAK1	4914	2	9228	1	5607	1	4587	1	5086	1	6107	1	1.00	0.53	0.66	0.50	0.61	0.55	
	NN041	ER protein 72	Pan-specific	ERP72	P13667	PDIA4_HUMA	PDIA4 ERP70 E	2539	1	4038	1	2121	1	1585	1	1514	1	2213	1	1.00	0.63	0.55	0.39	0.53	0.37	
	NN003	Adenylate kin	Pan-specific	AK2	P54819	KAD2_HUMAI	AK2 ADK2	6655	1	9831	1	6164	1	6591	1	4977	1	8649	1	1.00	0.68	0.88	0.67	0.63	0.51	
4	PK101-2	Ribosomal S6	S380/S386	RSK1/2	Q15418	KS6A1_HUMA	RPS6KA1 MAP	2303	1	5061	1	2506	1	2279	1	1820	1	3582	1	1.00	0.46	0.71	0.45	0.50	0.36	
	NK155-4	Raf1 proto-on	Pan-specific	Raf1	P04049	RAF1_HUMAN	RAF1 RAF	241	3	2932	2	405	1	719	2	340	2	345	1	1.00	0.08	0.12	0.25	0.14	0.12	
	PK091	Protein-serine	T410/T403	PKCz/l	Q05513	KPCZ_HUMAN	PRKCZ PKC2	570	1	1754	2	704	1	649	1	433	2	626	1	1.00	0.32	0.36	0.37	0.40	0.25	
5	PN048-2	Jun proto-onc	S73	Jun	P05412	JUN_HUMAN	JUN	9006	1	11658	1	6949	2	9658	1	4810	1	9874	2	1.00	0.77	0.85	0.83	0.60	0.41	
	PK003	Bone marrow	Y40	BMX (Etk)	P51813	BMX_HUMAN	BMX	1234	1	2082	1	1684	1	2878	1	1168	1	1859	1	0.72	0.43	0.65	1.00	0.59	0.41	
	PN101-2	Histone H3.3	T3	Histone H3	P84243	H33_HUMAN	H3F3A H3.3A	4668	1	7618	1	5273	1	7348	1	4176	1	7525	1	1.00	0.61	0.99	0.96	0.69	0.55	
	PN001	Eukaryotic tra	S64	4E-BP1	Q13541	4EBP1_HUMA	EIF4EBP1	6399	1	4877	1	1295	1	1223	1	1128	1	1088	1	0.76	1.00	0.17	0.19	0.20	0.18	
5	NK206-2	Extracellular r	Pan-specific	Erk5	Q13164	MK07_HUMAI	MAPK7 BMK1	1430	1	2367	1	869	2	633	1	902	1	746	1	1.00	0.60	0.32	0.27	0.37	0.38	
	NK110	Hepatocyte gr	Pan-specific	Met	P08581	MET_HUMAN	MET	593	1	2663	2	486	1	362	1	449	1	283	1	1.00	0.22	0.11	0.14	0.18	0.17	
	PN032	Forkhead-like	T32	FKHRL1	O43524	FOXO3_HUM/	FOXO3 FKHRL1	2604	1	4029	1	1836	2	1968	1	1803	2	1303	1	1.00	0.65	0.32	0.49	0.46	0.45	
	PN128	Eukaryotic tra	T69	4E-BP1	Q13541	4EBP1_HUMA	EIF4EBP1	2113	1	2699	1	1448	2	1431	1	1451	2	890	1	1.00	0.78	0.33	0.53	0.54	0.54	
3	NK120-4	Mitogen-activ	Pan-specific	p38a MAPK	Q16539	MK14_HUMAI	MAPK14 CSBP	4518	1	7309	1	4863	1	4558	1	3562	1	4937	1	1.00	0.62	0.68	0.62	0.67	0.49	
	NN098	Superoxide di	Pan-specific	SOD (Cu/Zn)	Q6ND84	Q6ND84_RHO	sodC RPA0225	5027	1	7968	1	4342	1	3955	1	3037	1	3488	1	1.00	0.63	0.44	0.50	0.54	0.38	
6	PK014-PK015-	Extracellular r	T202+Y204; T184/Y18	Erk1/2	P27361	MK03_HUMAI	MAPK3 ERK1	3429	1	4270	1	2878	1	2773	1	2394	1	2878	1	1.00	0.80	0.67	0.65	0.67	0.56	
4	NK056	Extracellular r	Pan-specific	Erk2	P28482	MK01_HUMAI	MAPK1 ERK2	2822	1	3958	2	3368	1	3734	1	2259	1	2803	1	1.00	0.71	0.71	0.94	0.85	0.57	
	NK022	Calcium/calm	Pan-specific	CaMKK (CaMKK2)	Q8N5S9	KKCC1_HUMA	CAMKK1 CAM	4647	1	7386	1	5825	1	6315	1	4160	1	5349	1	1.00	0.63	0.72	0.86	0.79	0.56	
	NK113-3	Mammalian S	Pan-specific	MST1	Q13043	STK4_HUMAN	STK4 KRS2 MS	3340	1	6726	1	4380	1	5444	1	3703	1	4183	1	1.00	0.50	0.62	0.81	0.65	0.55	
	NP009-2	Protein-serine	Pan-specific	PP1/Ca	P62136	PP1A_HUMAN	PPP1CA PPP1A	208	2	1094	1	147	2	203	3	92	2	163	2	1.00	0.19	0.15	0.19	0.13	0.08	
	PN020	Cofilin 2	S3	Cofilin 2	Q9Y281	COF2_HUMAN	CFL2	6144	1	7785	1	6569	1	8487	1	4682	1	5290	1	0.92	0.72	0.62	1.00	0.77	0.55	
5	NK105-2	MAPK/ERK pr	Pan-specific	MEK6 (MAP2K6)	P52564	MP2K6_HUM/	MAP2K6 MEK6	4330	1	4278	1	4121	1	6348	1	2399	1	3020	1	0.67	0.68	0.48	1.00	0.65	0.38	
7	PK014-PK015-	Extracellular r	T202+Y204; T184/Y18	Erk1/2	P27361	MK03_HUMAI	MAPK3 ERK1	6530	1	7729	1	5417	1	8310	1	3968	1	5245	1	0.93	0.79	0.63	1.00	0.65	0.48	
	PK065	Platelet-derive	Y716	PDGFRb	P09619	PGFRB_HUMA	PDGFRB PDGF	7144	1	8873	1	5053	1	7728	1	3901	1	4678	1	1.00	0.81	0.53	0.87	0.57	0.44	
	NK021	Calcium/calm	Pan-specific	CaMK4	Q16566	KCC4_HUMAN	CAMK4 CAMK	920	1	2653	1	630	1	667	1	479	2	498	2	1.00	0.35	0.19	0.25	0.24	0.18	
	NK048-2	DNA-depende	Pan-specific	DNAPK	P78527	PRKDC_HUMA	PRKDC HYRC	5971	1	7803	1	821	1	1119	1	594	1	805	1	1.00	0.77	0.10	0.14	0.11	0.08	
6	NK129-2	Protein-serine	Pan-specific	PKBa (Akt1)	P31749	AKT1_HUMAN	AKT1 PKB RAC	2955	1	6466	1	1769	1	2084	1	1489	1	1375	1	1.00	0.46	0.21	0.32	0.27	0.23	
	NK031-3	Cyclin-depend	Pan-specific	CDK8	P49336	CDK8_HUMAN	CDK8	3016	1	4033	1	2219	1	2729	1	1763	1	2464	1	1.00	0.75	0.61	0.68	0.55	0.44	
7	NK168-NK169-	p70/p85 ribos	Pan-specific	S6Ka (p70 S6Ka, p85 S6Ka)	P23443	KS6B1_HUMA	RPS6KB1 STK1	2751	1	6817	1	3535	1	4372	1	2072	1	3079	1	1.00	0.40	0.45	0.64	0.52	0.30	
	NK089	Kinase homol	Pan-specific	KHS	Q9Y4K4	M4K5_HUMAI	MAP4K5	3435	1	6838	1	3848	2	4973	2	3247	2	1147	1	1.00	0.50	0.17	0.73	0.56	0.47	
8	PK026-PK027-	Glycogen synt	S21/S9	GSK3ab	P49840	GSK3A_HUMA	GSK3A	2871	1	4099	1	3507	1	4453	1	2252	1	1674	1	0.92	0.64	0.38	1.00	0.79	0.51	
8	NN102-NN124	Signal transdu	Pan-specific	STAT1a/b	P42224	STAT1_HUMA	STAT1	1477	1	4144	1	1631	1	1566	2	1978	1	1050	1	1.00	0.36	0.25	0.38	0.39	0.48	
	NK107-3	MAPK/ERK kin	Pan-specific	MEKK1 (MAP3K1)	Q13233	M3K1_HUMAN	MAP3K1 MAP	2673	1	3771	1	2397	1	2559	1	2401	1	2081	1	1.00	0.71	0.55	0.68	0.64	0.64	
	NK205	A-Raf proto-o	Pan-specific	RafA (Araf)	P10398	ARAF_HUMAN	ARAF ARAF1 P	3994	1	5958	1	3753	1	3974	1	3358	1	2837	1	1.00	0.67	0.48	0.67	0.63	0.56	
	NK159-2	RhoA protein-	Pan-specific	ROKa (ROCK2)	O75116	ROCK2_HUMA	ROCK2 KIAA0	6272	1	9228	1	6100	1	4504	1	4133	1	3244	1	1.00	0.68	0.35	0.49	0.66	0.45	
	NK156-2	RafB proto-on	Pan-specific	RafB (Braf)	P15056	BRAF_HUMAN	BRAF BRAF1 R	2768	1	6282	1	3424	2	4452	1	2403	2	1580	2	1.00	0.44	0.25	0.71	0.55	0.38	
	NN046	GroEL homolo	Pan-specific	GroEL	P10809	CH60_HUMAN	HSPD1 HSP60	10072	1	10351	1	7936	1	9708	1	5576	1	6118	1	1.00	0.97	0.59	0.94	0.77	0.54	
6	PK028-PK029-	Glycogen synt	Y279/ Y216	GSK3ab	P49840	GSK3A_HUMA	GSK3A	7025	1	9306	1	4960	1	4510	1	3063	2	2568	2	1.00	0.75	0.28	0.48	0.53	0.33	
	PN111	Synapsin 1 iso	S603	Synapsin 1	P17600	SYN1_HUMAN	SYN1	2196	1	5902	1	1696	1	1881	1	1874	1	1613	1	1.00	0.37	0.27	0.32	0.29	0.32	
9	NK197	Jun N-terminu	Pan-specific	JNK3	P53779	MK10_HUMAI	MAPK10 JNK3	301	2	759	2	117	2	129	2	99	2	64	2	1.00	0.40	0.08	0.17	0.15	0.13	
	NN049-2	Glucose regul	Pan-specific	Grp94	P14625	ENPL_HUMAN	HSP90B1 GRP9	856	1	2313	2	731	2	1003	2	554	2	625	1	1.00	0.37	0.27	0.43	0.32	0.24	
	NK141	Protein-serine	Pan-specific	PKCz	Q05513	KPCZ_HUMAN	PRKCZ PKC2	2794	1	6307	1	3955	2	3742	2	2678	2	3191	2	1.00	0.44	0.51	0.59	0.63	0.42	
	NN155	Myelin expres	Pan-specific	MEF-2	Q9P2K5	MYEF2_HUM/	MYEF2 KIAA13	1277	2	4921	2	2541	1	2390	1	2698	1	2894	1	1.00	0.26	0.59	0.49	0.52	0.55	
	NN040	ER protein 57	Pan-specific	ERP57	P30101	PDIA3_HUMA	PDIA3 ERP57	2555	1	2124	1	2074	1	1126	1	1991	2	1372	1	0.83	1.00	0.54	0.44	0.81	0.78	8
	PP005	Cell division c	S216	Cdc25C	Q13972	RGRF1_HUMA	RASGRF1 CDC	1715	1	2776	2	1832	1	1730	1	2218	2	1132	1	1.00	0.62	0.41	0.62	0.66	0.80	
	PK007-1	Cyclin-depend	Y15	CDK1/2	P06493	CDK1_HUMAN	CDK1 CDC2 CD	2656	1	4686	1	3815	1	3340	1	3781	1	3685	1	1.00	0.57	0.79	0.71	0.81	0.81	
9	PK105-PK141	p70/p85 ribos	T444+S447, T421+S42	S6Ka (p70 S6Ka, p85 S6Ka)	P23443	KS6B1_HUMA	RPS6KB1 STK1	3418	1	6572	1	5983	1	4944	1	6785	1	5654	1	0.97	0.50	0.83	0.73	0.88	1.00	
10	PK127	MAPK/ERK pr	S218	MEK3 (MAP2K3)	P46734	MP2K3_HUM/	MAP2K3 MEK3	2988	1	6279	1	4028	1	2237	1	4385	1	3429	1	1.00	0.48	0.55	0.36	0.64	0.70	
	PN091	Microtubule-a	S716	Tau	P10636	TAU_HUMAN	MAPT MAPTL	3893	1	9592	1	6955	1	3782	2	7570	1	6531	1	1.00	0.41	0.68	0.39	0.73	0.79	
	NP021-2	Protein-serine	Pan-specific	PP5C	P53041	PPP5_HUMAN	PPP5C PPP5	2769	1	4122	1	3315	1	2170	1	2406	1	2338	1	1.00	0.67	0.57	0.53	0.80	0.58	
7	PK100	Ribosomal S6	S363/S369	RSK1/2	Q15418	KS6A1_HUMA	RPS6KA1 MAP	6528	1	8698	1	7342	1	4128	1	4968	1	4108	2	1.00	0.75	0.47	0.47	0.84	0.57	
	PN046-2	Insulin recept	Y1179	IRS1	P35568	IRS1_HUMAN	IRS1	4565	1	9029	1	6906	1	6359	1	5786	1	3647	2	1.00	0.51	0.40	0.70	0.76	0.64	
	NK113-1	Mammalian S	Pan-specific	MST1	Q13043	STK4_HUMAN	STK4 KRS2 MS	4762	1	7710	1	6076	1	5135	1	5454	1	4364	1	1.00	0.62	0.57	0.67	0.79	0.71	
10	NN080	p27 cyclin-dep	Pan-specific	p27 Kip1	P46527	CDN1B_HUM/	CDKN1B KIP1	2021	1	4469	1	2103	1	1950	1	2167	1	1547	1	1.00	0.45	0.35	0.44	0.47	0.48	
	PN113	Retinoblastom	S608	Rb	P06400	RB_HUMAN	RB1	2123	2	6137	1	4553	1	4229	1	4405	1	3815	1	1.00	0.35	0.62	0.69	0.74	0.72	
	NK026-2	Cyclin-depend	Pan-specific	CDK2	P24941	CDK2_HUMAN	CDK2 CDKN2	1074	1	3288	1	1362	1	1104	1	1503	1	1384	1	1.00	0.33	0.42	0.34	0.41	0.46	
	PN114	Eukaryotic tra	T45	4E-BP1	Q13541	4EBP1_HUMA	EIF4EBP1	4830	1	8940	1	5504	1	4994	1	5850	1	6146	1	1.00	0.54	0.69	0.56	0.62	0.65	
8	PK100-2	Ribosomal S6	S363/S369	RSK1/2	Q15418	KS6A1_HUMA	RPS6KA1 MAP	4239	1	7943	1	5024	1	4954	1	5607	1	4862	1	1.00	0.53	0.61	0.62	0.63	0.71	
	PN109	Tyrosine hydr	S19	Tyrosine Hydroxylase	P07101	TY3H_HUMAN	TH TYH	2601	1	4378	1	2493	1	2384	1	2995	1	2614	1	1.00	0.59	0.60	0.54	0.57	0.68	
	NK123	p21-activated	Pan-specific	PAK3	O75914	PAK3_HUMAN	PAK3 OPHN3	6466	1	9223	1	6167	1	4978	1	7047	1	6037	1	1.00	0.70	0.65	0.54	0.67	0.76	
	NK090	Protein-serine	Pan-specific	Ksr1	Q8IVT5	KSR1_HUMAN	KSR1 KSR	3717	1	7016	1	3082	1	3655	1	4405	1	3641	1	1.00	0.53	0.52	0.52	0.44	0.63	
	NK143	Protein-serine	Pan-specific	PKG1	Q13976	KGP1_HUMAN	PRKG1 PRKG1	5132	1	9685	1	5429	1	6051	1	6645	1	5149	1	1.00	0.53	0.53	0.62	0.56	0.69	
	NK126-2	3-phosphoino	Pan-specific	PDK1	O15530	PDPK1_HUMA	PDPK1 PDK1	3215	1	7061	1	3246	1	3225	1	3282	1	2231	1	1.00	0.46	0.32	0.46	0.46	0.46	
	NK188-2	ZIP kinase (de	Pan-specific	ZIPK	O43293	DAPK3_HUM/	DAPK3 ZIPK	3474	1	7011	1	3591	1	3392	1	4055	1	2724	1	1.00	0.50	0.39	0.48	0.51	0.58	
9	NK101	MAPK/ERK pr	Pan-specific	MEK3 (MAP2K3)	P46734	MP2K3_HUM/	MAP2K3 MEK3	2949	1	4052	1	2869	1	2891	1	3200	1	2096	1	1.00	0.73	0.52	0.71	0.71	0.79	
10	PK048-2	MAPK/ERK pr	T385	MEK1 (MAP2K1)	Q02750	MP2K1_HUM/	MAP2K1 MEK1	3760	1	7908	1	3116	1	3187	1	3417	1	2617	1	1.00	0.48	0.33	0.40	0.39	0.43	
	NK142	Protein-serine	Pan-specific	PKCm (PKD)	Q15139	KPCD1_HUMA	PRKD1 PKD PK	4678	1	6309	1	3271	1	3297	1	3810	1	2207	1	1.00	0.74	0.35	0.52	0.52	0.60	
11	NK172-4	Src proto-onc	Pan-specific	Src	P12931	SRC_HUMAN	SRC SRC1	4260	1	5936	1	3284	1	3139	1	4035	1	2342	1	1.00	0.72	0.39	0.53	0.55	0.68	
	PN117	Insulin recept	S312	IRS1	P35568	IRS1_HUMAN	IRS1	3963	1	7649	1	5055	1	4403	1	5727	1	3045	1	1.00	0.52	0.40	0.58	0.66	0.75	
	PN118	Insulin recept	S639	IRS1	P35568	IRS1_HUMAN	IRS1	3333	1	6363	1	4629	1	4320	1	5388	1	2835	1	1.00	0.52	0.45	0.68	0.73	0.85	
12	NK060	Focal adhesio	Pan-specific	FAK	Q05397	FAK1_HUMAN	PTK2 FAK FAK1	6456	1	9151	1	6381	1	5854	1	7190	1	5064	1	1.00	0.71	0.55	0.64	0.70	0.79	
	NK007	Apoptosis sigr	Pan-specific	ASK1 (MAP3K5)	Q99683	M3K5_HUMAI	MAP3K5 ASK1	4291	1	5815	1	4113	1	4109	1	4998	1	3318	1	1.00	0.74	0.57	0.71	0.71	0.86	
	NN122	Hsp 70-relatec	Pan-specific	APG2	P34932	HSP74_HUMA	HSPA4 APG2	4905	1	7962	1	4282	1	4347	1	5107	1	3422	1	1.00	0.62	0.43	0.55	0.54	0.64	
	NN057	DnaJ homolog	Pan-specific	Hsp40	P25685	DNJB1_HUMA	DNAJB1 DNAJ1	2763	1	6824	1	2900	1	3179	1	3260	1	2248	1	1.00	0.40	0.33	0.47	0.42	0.48	
	NN071	NF-kappa-B p(	Pan-specific	NFkappaB p65	Q04206	TF65_HUMAN	RELA NFKB3	7581	1	10637	1	6379	1	5941	1	6589	1	4826	1	1.00	0.71	0.45	0.56	0.60	0.62	
	NN062	Heat shock 10	Pan-specific	Hsp105	Q92598	HS105_HUMA	HSPH1 HSP105	4541	1	7826	1	3845	1	3855	1	4514	1	2649	1	1.00	0.58	0.34	0.49	0.49	0.58	
11	PK022-2	Focal adhesio	S843	FAK	Q05397	FAK1_HUMAN	PTK2 FAK FAK1	1212	1	2447	1	626	1	907	2	1338	1	300	1	1.00	0.50	0.12	0.37	0.26	0.55	
	NK204	PSTAIRE	Pan-specific	PSTAIRE	NA			2080	1	3051	1	1864	1	1449	1	1995	1	1722	1	1.00	0.68	0.56	0.47	0.61	0.65	
12	PK046-2	MAPK/ERK pr	T291	MEK1 (MAP2K1)	Q02750	MP2K1_HUM/	MAP2K1 MEK1	606	1	1685	2	718	1	682	1	745	1	538	1	1.00	0.36	0.32	0.40	0.43	0.44	
	PK118	Apoptosis sigr	S1046	ASK1 (MAP3K5)	Q99683	M3K5_HUMAI	MAP3K5 ASK1	711	1	1959	1	696	1	789	2	783	1	580	1	1.00	0.36	0.30	0.40	0.36	0.40	
13	NK186	Yamaguchi sai	Pan-specific	Yes	P07947	YES_HUMAN	YES1 YES	288	2	505	2	366	1	376	1	504	1	320	1	1.00	0.57	0.63	0.74	0.73	1.00	
14	NK168-NK169-	p70/p85 ribos	Pan-specific	S6Ka (p70 S6Ka, p85 S6Ka)	P23443	KS6B1_HUMA	RPS6KB1 STK1	521	5	1921	2	912	1	895	1	1178	1	786	1	1.00	0.27	0.41	0.47	0.47	0.61	
11	PN057-2	Tumor suppre	S392	p53	P04637	P53_HUMAN	TP53 P53	287	1	1619	1	654	1	488	2	878	1	522	2	1.00	0.18	0.32	0.30	0.40	0.54	
15	NK088-3	Jun N-terminu	Pan-specific	JNK1/2/3	P45983	MK08_HUMAI	MAPK8 JNK1	444	1	1072	1	677	1	1006	1	931	1	669	1	1.00	0.41	0.62	0.94	0.63	0.87	
	PK087	Protein-serine	T555	PKCl/i	P41743	KPCI_HUMAN	PRKCI DXS1179	605	1	2001	2	1392	1	1513	1	1800	1	907	1	1.00	0.30	0.45	0.76	0.70	0.90	
	PK093-2	Protein-serine	S910	PKCm (PKD)	Q15139	KPCD1_HUMA	PRKD1 PKD PK	1123	1	2741	1	2386	1	3146	1	3029	1	1562	1	0.87	0.36	0.50	1.00	0.76	0.96	
	NK025-4	Cyclin-depend	Pan-specific	CDK1 (CDC2)	P06493	CDK1_HUMAN	CDK1 CDC2 CD	2364	1	2815	1	1513	1	1684	1	1598	1	1688	1	1.00	0.84	0.60	0.60	0.54	0.57	9
	NK103-3	MAPK/ERK pr	Pan-specific	MEK4 (MAP2K4)	P45985	MP2K4_HUM/	MAP2K4 JNKK	2941	1	5108	1	2356	1	3688	1	2761	1	2564	1	1.00	0.58	0.50	0.72	0.46	0.54	
	NN025	Cellular inhibi	Pan-specific	c-IAP1	Q13490	BIRC2_HUMAI	BIRC2 API1 IAF	3198	1	5271	1	2072	1	2052	1	2192	1	2096	1	1.00	0.61	0.40	0.39	0.39	0.42	
16	NN106	Signal transdu	Pan-specific	STAT5B	P51692	STA5B_HUMA	STAT5B	6572	1	7894	1	4181	1	3607	1	4527	1	4415	1	1.00	0.83	0.56	0.46	0.53	0.57	
12	PK129	MAPK/ERK pr	S207	MEK6 (MAP2K6)	P52564	MP2K6_HUM/	MAP2K6 MEK6	4204	1	4805	1	2983	1	2441	1	3063	1	3388	1	1.00	0.88	0.71	0.51	0.62	0.64	
17	NN102-NN124	Signal transdu	Pan-specific	STAT1a/b	P42224	STAT1_HUMA	STAT1	393	2	531	1	245	1	67	2	278	1	193	1	1.00	0.74	0.36	0.13	0.46	0.52	
13	PN082	Signal transdu	Y705	STAT3	P40763	STAT3_HUMA	STAT3 APRF	1823	1	4705	1	1263	1	760	1	1068	1	955	1	1.00	0.39	0.20	0.16	0.27	0.23	
13	PN042-2	Heat shock 27	S15	Hsp27	P04792	HSPB1_HUMA	HSPB1 HSP27	2611	1	7353	1	2605	1	2243	1	3053	1	3578	1	1.00	0.36	0.49	0.31	0.35	0.42	
	NK158	Receptor-inte	Pan-specific	RIPK1	Q13546	RIPK1_HUMAI	RIPK1 RIP RIP1	12634	1	16092	1	7783	1	7633	1	8545	1	9016	1	1.00	0.79	0.56	0.47	0.48	0.53	
	NK175-5	TGF-beta-acti	Pan-specific	TAK1	O43318	M3K7_HUMAI	MAP3K7 TAK1	5509	1	8407	1	4138	1	3830	1	5057	1	5386	1	1.00	0.66	0.64	0.46	0.49	0.60	
	PN051-2	Myosin regula	S19	MLC(MLRC2)	P19105	ML12A_HUM/	MYL12A MLCB	1831	1	2684	1	1333	1	1158	1	1100	1	1409	1	1.00	0.68	0.53	0.43	0.50	0.41	
	PK010	Epidermal gro	Y1172	EGFR	P00533	EGFR_HUMAN	EGFR ERBB ER	1906	1	6262	1	1458	1	1130	1	1405	1	1781	1	1.00	0.30	0.28	0.18	0.23	0.22	
	NN030-2	Cyclin D1 (PR/	Pan-specific	Cyclin D1	P24385	CCND1_HUM/	CCND1 BCL1 P	1215	1	2265	1	784	1	736	1	638	1	1285	2	1.00	0.54	0.57	0.32	0.35	0.28	
	PN075	Mothers agair	S463+S465/S463+S465	Smad1/5/9	Q15797	SMAD1_HUM	SMAD1 BSP1	6103	1	7285	1	1106	1	1103	2	1016	2	1834	1	1.00	0.84	0.25	0.15	0.15	0.14	
	NP033	Protein-serine	Pan-specific	PP2A B' (B56)	Q15172	2A5A_HUMAN	PPP2R5A	11052	1	9036	1	4575	1	4623	1	4778	1	4370	1	0.82	1.00	0.40	0.42	0.41	0.43	10
	NN133	Striatin-3	Pan-specific	SG2NA	Q13033	STRN3_HUMA	STRN3 GS2NA	13431	1	9631	1	4743	1	5450	1	5345	1	4962	1	0.72	1.00	0.37	0.41	0.35	0.40	
	NN151	Superoxide di	Pan-specific	SOD (Mn)	P04179	SODM_HUMA	SOD2	8535	1	8377	1	3997	1	4112	1	4170	1	4102	1	0.98	1.00	0.48	0.48	0.47	0.49	
	NK157	Receptor-inte	Pan-specific	RIP2/RICK	O43353	RIPK2_HUMAI	RIPK2 CARDIAK	10691	1	14830	1	8027	1	9524	1	8560	1	7900	1	1.00	0.72	0.53	0.64	0.54	0.58	
	NK160	RhoA protein-	Pan-specific	ROKb (ROCK1)	Q13464	ROCK1_HUM/	ROCK1	12026	1	15853	1	8495	1	10034	1	9168	1	8773	1	1.00	0.76	0.55	0.63	0.54	0.58	
	NP024	Protein-tyrosi	Pan-specific	PTP1B	P18031	PTN1_HUMAN	PTPN1 PTP1B	10408	1	13717	1	8198	1	8838	1	9210	1	7741	1	1.00	0.76	0.56	0.64	0.60	0.67	
	NP016	Protein-serine	Pan-specific	PP2Ca	P35813	PPM1A_HUM	PPM1A PPPM1	8692	1	12601	1	6418	1	6158	1	7018	1	6063	1	1.00	0.69	0.48	0.49	0.51	0.56	
	PN065	Retinoblastom	T356	Rb	P06400	RB_HUMAN	RB1	10162	1	14269	1	7998	1	9415	1	9730	1	8822	1	1.00	0.71	0.62	0.66	0.56	0.68	
	NN027	Cyclo-oxygena	Pan-specific	COX2	P35354	PGH2_HUMAI	PTGS2 COX2	11581	1	12313	1	6652	1	8541	1	8491	1	8039	1	1.00	0.94	0.65	0.69	0.54	0.69	
	NN024	Cell division c	Pan-specific	Cdc42	P60953	CDC42_HUMA	CDC42	10339	1	12015	1	6711	1	7790	1	7813	1	7959	1	1.00	0.86	0.66	0.65	0.56	0.65	
14	PN048-1	Jun proto-onc	S73	Jun	P05412	JUN_HUMAN	JUN	4699	1	7031	1	2068	1	2563	1	2249	1	2945	1	1.00	0.67	0.42	0.36	0.29	0.32	
	NK144-1	Double strand	Pan-specific	PKR1	P19525	E2AK2_HUMA	EIF2AK2 PKR P	6867	1	3754	1	2923	1	3251	1	3501	1	3711	1	0.55	1.00	0.54	0.47	0.43	0.51	
	NN053	Heme oxygen	Pan-specific	HO2	P30519	HMOX2_HUM	HMOX2 HO2	10705	1	8854	1	3840	1	4882	1	3855	1	3755	1	0.83	1.00	0.35	0.46	0.36	0.36	
	NN061-16	Heat shock 90	Pan-specific	Hsp90a	P07900	HS90A_HUMA	HSP90AA1 HS	9426	1	11936	1	3692	1	4585	1	3927	1	3581	1	1.00	0.79	0.30	0.38	0.31	0.33	
	NN048-2	Glucose regul	Pan-specific	Grp78	P11021	GRP78_HUMA	HSPA5 GRP78	8601	1	6454	1	3573	1	3669	1	3722	1	3428	1	0.75	1.00	0.40	0.43	0.42	0.43	
	NN060-3	Heat shock 70	Pan-specific	Hsp70	P08107	HSP71_HUMA	HSPA1A HSPA1	8439	1	7574	1	3592	1	4153	1	4036	1	3740	1	0.90	1.00	0.44	0.49	0.43	0.48	
	NN137-1	Calreticulin	Pan-specific	Calreticulin	P27797	CALR_HUMAN	CALR CRTC	9769	1	8429	1	3907	1	4583	1	5305	1	4873	1	0.86	1.00	0.50	0.47	0.40	0.54	
	NN136-1	Calnexin	Pan-specific	Calnexin	P27824	CALX_HUMAN	CANX	10136	1	9929	1	2733	1	2767	1	3172	1	3141	1	0.98	1.00	0.31	0.27	0.27	0.31	
	NN136-2	Calnexin	Pan-specific	Calnexin	P27824	CALX_HUMAN	CANX	8488	1	6506	1	2799	1	3236	1	3510	1	3335	1	0.77	1.00	0.39	0.38	0.33	0.41	
	NN149-1	Crystallin alph	Pan-specific	Crystallin aB	P02511	CRYAB_HUMA	CRYAB CRYA2	9791	1	12528	1	3326	1	3741	1	3600	1	3771	1	1.00	0.78	0.30	0.30	0.27	0.29	
	PN025	Crystallin alph	S19	Crystallin aB	P02511	CRYAB_HUMA	CRYAB CRYA2	14785	1	11264	1	4054	1	5536	1	5312	1	4876	1	0.76	1.00	0.33	0.37	0.27	0.36	
	PN110	Crystallin alph	S45	Crystallin aB	P02511	CRYAB_HUMA	CRYAB CRYA2	8732	1	10941	1	3649	1	4866	1	4672	1	4436	1	1.00	0.80	0.41	0.44	0.33	0.43	
	NN010	Cellular apopt	Pan-specific	CAS	P55060	XPO2_HUMAI	CSE1L CAS XPO	3059	1	7244	1	1846	1	2336	1	2321	1	2362	1	1.00	0.42	0.33	0.32	0.25	0.32	
	NP001	Leukocyte cor	Pan-specific	CD45	P08575	PTPRC_HUMA	PTPRC CD45	10523	1	12096	1	5784	1	7236	1	7020	1	8171	1	1.00	0.87	0.68	0.60	0.48	0.58	
	PN018	Caveolin 2	S36	Caveolin 2	P51636	CAV2_HUMAI	CAV2	8753	1	11197	1	5778	1	7418	1	7322	1	7372	1	1.00	0.78	0.66	0.66	0.52	0.65	
	NN061-15	Heat shock 90	Pan-specific	Hsp90a	P07900	HS90A_HUMA	HSP90AA1 HS	6144	1	9572	1	2720	1	3035	1	3100	1	3248	1	1.00	0.64	0.34	0.32	0.28	0.32	
	NN061-7	Heat shock 90	Pan-specific	Hsp90	P07900	HS90A_HUMA	HSP90AA1 HS	5562	1	6502	1	3006	1	3416	1	3635	1	3351	1	1.00	0.86	0.52	0.53	0.46	0.56	
14	PN041	Heat shock 27	S78	Hsp27	P04792	HSPB1_HUMA	HSPB1 HSP27	11344	1	11295	1	3483	1	4997	1	4267	1	4400	1	1.00	1.00	0.39	0.44	0.31	0.38	
	NN054-2	Heat shock 70	Pan-specific	Hsc70	P11142	HSP7C_HUMA	HSPA8 HSC70	10208	1	9382	1	3707	1	4666	1	4486	1	4857	1	0.92	1.00	0.48	0.46	0.36	0.44	
18	NN152-1	Heat shock 27	Pan-specific	Hsp27	P04792	HSPB1_HUMA	HSPB1 HSP27	8905	1	9088	1	4634	1	5815	1	5456	1	5193	1	1.00	0.98	0.57	0.64	0.51	0.60	
	NN140-1	Nuclear factor	Pan-specific	Nrf2	Q16236	NF2L2_HUMA	NFE2L2 NRF2	6799	1	7936	1	3079	1	3797	1	3695	1	3852	1	1.00	0.86	0.49	0.48	0.39	0.47	
	NN054-3	Heat shock 70	Pan-specific	Hsc70	P11142	HSP7C_HUMA	HSPA8 HSC70	6475	1	8746	1	2992	1	3276	1	3483	1	3575	1	1.00	0.74	0.41	0.37	0.34	0.40	
	NN061-13	Heat shock 90	Pan-specific	Hsp90a	P07900	HS90A_HUMA	HSP90AA1 HS	5867	1	6640	1	3097	1	3388	1	4224	1	3958	1	1.00	0.88	0.60	0.51	0.47	0.64	
	NN061-6	Heat shock 90	Pan-specific	Hsp90	P07900	HS90A_HUMA	HSP90AA1 HS	6684	1	6944	1	2703	1	3720	1	3783	1	4011	1	1.00	0.96	0.58	0.54	0.39	0.54	
	NN061-12	Heat shock 90	Pan-specific	Hsp90a	P07900	HS90A_HUMA	HSP90AA1 HS	7545	1	7304	1	2892	1	3411	1	3667	1	3393	1	0.97	1.00	0.45	0.45	0.38	0.49	
	NN061-10	Heat shock 90	Pan-specific	Hsp90	P07900	HS90A_HUMA	HSP90AA1 HS	7369	1	5492	1	2601	1	2852	1	3005	1	2958	1	0.75	1.00	0.40	0.39	0.35	0.41	
	NN061-5	Heat shock 90	Pan-specific	Hsp90	P07900	HS90A_HUMA	HSP90AA1 HS	6748	1	6315	1	2284	1	2437	1	2726	1	2685	1	0.94	1.00	0.40	0.36	0.34	0.40	
19	NN120-5	Mitogen-activ	Pan-specific	p38a MAPK	Q16539	MK14_HUMAI	MAPK14 CSBP	10017	1	6998	1	3103	1	3676	1	3584	1	3658	1	0.70	1.00	0.37	0.37	0.31	0.36	
	NN130	Acidic leucine	Pan-specific	I1PP2A	P39687	AN32A_HUM/	ANP32A C15or	10301	1	10412	1	2665	1	2952	1	2828	1	3166	1	1.00	0.99	0.30	0.28	0.26	0.27	
	NN131	Protein SET	Pan-specific	I2PP2A	Q01105	SET_HUMAN	SET	9648	1	6447	1	2767	1	3122	1	2854	1	3319	1	0.67	1.00	0.34	0.32	0.29	0.30	
	NN051	Hsp70/Hsc70	Pan-specific	Hip	P50502	F10A1_HUMA	ST13 AAG2 FA	10679	1	9526	1	3167	1	3661	1	3275	1	3786	1	0.89	1.00	0.35	0.34	0.30	0.31	
	NN052	Heme oxygen	Pan-specific	HO1	P09601	HMOX1_HUM	HMOX1 HO HO	11101	1	7866	1	3195	1	3601	1	3254	1	3867	1	0.71	1.00	0.35	0.32	0.29	0.29	
	NK133-2	Protein-serine	Pan-specific	PKCb1	P05771	KPCB_HUMAN	PRKCB PKCB P	4642	1	7769	1	3723	1	4612	1	4583	1	4851	1	1.00	0.60	0.62	0.59	0.48	0.59	
	PN043	Integrin alpha	S988	Integrin a4	P13612	ITA4_HUMAN	ITGA4 CD49D	8058	1	8933	1	3180	1	3864	1	3386	1	3613	1	1.00	0.90	0.40	0.43	0.36	0.38	
	NK052-1	Epidermal gro	Pan-specific	EGFR	P00533	EGFR_HUMAN	EGFR ERBB ER	11175	1	8873	1	3832	1	4825	1	4203	1	4915	1	0.79	1.00	0.44	0.43	0.34	0.38	
	PK134	ErbB2 (Neu) r	T686	ErbB2 (HER2)	P04626	ERBB2_HUMA	ERBB2 HER2 M	11563	1	11554	1	2485	1	3397	1	2810	1	3086	1	1.00	1.00	0.27	0.29	0.21	0.24	
	NK212	Serine/threon	Pan-specific	Nlk	Q9UBE8	NLK_HUMAN	NLK LAK1	5608	1	5564	1	3616	1	3135	1	4405	1	4471	1	0.99	1.00	0.80	0.56	0.64	0.79	11
	NK097	Mitogen-activ	Pan-specific	MAPKAPK2	P49137	MAPK2_HUM	MAPKAPK2	4883	1	5988	1	4287	1	4009	1	4934	1	4556	1	1.00	0.82	0.76	0.67	0.72	0.82	
	NK002	Activated p21	Pan-specific	ACK1	Q07912	ACK1_HUMAN	TNK2 ACK1	4711	1	4687	1	2982	1	2476	1	3779	1	3706	1	0.99	1.00	0.79	0.53	0.63	0.80	
	NK207	NF-kappa beta	Pan-specific	NIK	Q99558	M3K14_HUM/	MAP3K14 NIK	4850	1	6715	1	2790	1	1858	1	3407	1	3557	1	1.00	0.72	0.53	0.28	0.42	0.51	
20	NK120-10	Mitogen-activ	Pan-specific	p38a MAPK	Q16539	MK14_HUMAI	MAPK14 CSBP	472	1	626	2	220	1	172	1	308	1	291	1	1.00	0.75	0.46	0.27	0.35	0.49	
	NN149	Crystallin alph	Pan-specific	Crystallin aB	P02511	CRYAB_HUMA	CRYAB CRYA2	6167	1	4914	1	1863	1	2087	1	2915	1	2943	1	0.80	1.00	0.48	0.34	0.30	0.47	
	NN154	Ubiquitin	Pan-specific	Ubiquitin	P62979, P629	RS27A_HUMA	RPS27A UBA8	5669	1	8774	1	2595	1	2993	1	3233	1	3173	1	1.00	0.65	0.36	0.34	0.30	0.37	
	NK134-2	Protein-serine	Pan-specific	PKCb2	P05771	KPCB_HUMAN	PRKCB PKCB P	4695	1	7047	1	3787	1	3825	1	4622	1	4140	1	1.00	0.67	0.59	0.54	0.54	0.66	
	NN065	Inhibitor of NI	Pan-specific	IkBb	Q15653	IKBB_HUMAN	NFKBIB IKBB T	1779	1	4679	1	1128	1	1053	1	1420	1	1533	1	1.00	0.38	0.33	0.22	0.24	0.30	
	NK122-2	p21-activated	Pan-specific	PAK1	Q13153	PAK1_HUMAN	PAK1	1482	1	1660	1	1014	2	621	1	913	1	995	1	1.00	0.89	0.60	0.37	0.61	0.55	
	PN069	Retinoblastom	S807+S811	Rb	P06400	RB_HUMAN	RB1	5702	1	8482	1	2212	2	617	1	3474	1	2996	1	1.00	0.67	0.35	0.07	0.26	0.41	
21	NN103	Signal transdu	Pan-specific	STAT2	P52630	STAT2_HUMA	STAT2	7169	1	6677	1	4531	1	3703	1	5481	1	5163	1	0.93	1.00	0.72	0.52	0.63	0.76	
	NK205-2	A-Raf proto-o	Pan-specific	RafA (Araf)	P10398	ARAF_HUMAN	ARAF ARAF1 P	6240	1	6334	1	3379	1	3131	1	4470	1	4627	1	1.00	0.99	0.73	0.49	0.53	0.71	
	NN059-1	Heat shock 60	Pan-specific	Hsp60	P10809	CH60_HUMAN	HSPD1 HSP60	5616	1	7814	1	3350	1	2644	1	3601	1	3717	1	1.00	0.72	0.48	0.34	0.43	0.46	
22	NN107	Signal transdu	Pan-specific	STAT6	P42226	STAT6_HUMA	STAT6	7014	1	10782	1	4163	1	3750	1	5295	1	4911	1	1.00	0.65	0.46	0.35	0.39	0.49	
	PK030-PK031	Inhibitor of NI	S180/S181	IKKa/b	O15111	IKKA_HUMAN	CHUK IKKA TCI	1705	1	4579	1	289	1	361	1	436	1	332	1	1.00	0.37	0.07	0.08	0.06	0.10	
15	NN021	Catenin (cadh	Pan-specific	Catenin b1	P35222	CTNB1_HUM/	CTNNB1 CTNN	7209	1	8878	1	4064	1	4276	1	5581	1	4791	1	1.00	0.81	0.54	0.48	0.46	0.63	
	NN044	Fos-c FBJ mur	Pan-specific	Fos	P01100	FOS_HUMAN	FOS G0S7	7020	1	8333	1	4355	1	4255	1	5046	1	4267	1	1.00	0.84	0.51	0.51	0.52	0.61	
23	NK172-3	Src proto-onc	Pan-specific	Src	P12931	SRC_HUMAN	SRC SRC1	4941	1	4399	1	2028	1	1958	1	2986	1	2361	1	0.89	1.00	0.48	0.40	0.41	0.60	
	NK218	Protein kinase	Pan-specific	PKC h	P24723	KPCL_HUMAN	PRKCH PKCL P	4939	1	7939	1	1496	1	1431	1	1899	1	1796	1	1.00	0.62	0.23	0.18	0.19	0.24	
	NK036	Casein protein	Pan-specific	CK1d	P48730	KC1D_HUMA	CSNK1D HCKID	6231	1	9289	1	191	5	1673	2	1431	7	5076	1	1.00	0.67	0.55	0.18	0.02	0.15	
	NN061-14	Heat shock 90	Pan-specific	Hsp90a	P07900	HS90A_HUMA	HSP90AA1 HS	5568	1	6750	1	2817	1	2782	1	3816	1	3615	1	1.00	0.82	0.54	0.41	0.42	0.57	
	NN061-8	Heat shock 90	Pan-specific	Hsp90	P07900	HS90A_HUMA	HSP90AA1 HS	6115	1	6932	1	2839	1	3062	1	3672	1	3893	1	1.00	0.88	0.56	0.44	0.41	0.53	
	NK044-2	C-terminus of	Pan-specific	Csk	P41240	CSK_HUMAN	CSK	5633	1	8079	1	2816	1	3012	1	3987	1	3945	1	1.00	0.70	0.49	0.37	0.35	0.49	
	NP006	MAP kinase pl	Pan-specific	MKP1	P28562	DUS1_HUMAN	DUSP1 CL100	5087	1	8597	1	2487	1	2535	1	3787	1	3825	1	1.00	0.59	0.44	0.29	0.29	0.44	
	NK016-2	Calcium/calm	Pan-specific	CaMK1d	Q8IU85	KCC1D_HUMA	CAMK1D CAM	7058	1	5163	1	2116	1	2583	1	3120	1	3372	1	0.73	1.00	0.48	0.37	0.30	0.44	
	NK025-5	Cyclin-depend	Pan-specific	CDK1 (CDC2)	P06493	CDK1_HUMAN	CDK1 CDC2 CD	4417	1	3374	1	1191	1	1338	1	1667	1	1856	1	0.76	1.00	0.42	0.30	0.27	0.38	
	NK025-6	Cyclin-depend	Pan-specific	CDK1 (CDC2)	P06493	CDK1_HUMAN	CDK1 CDC2 CD	3717	1	5169	1	1673	1	1774	1	2235	1	2477	1	1.00	0.72	0.48	0.34	0.32	0.43	
	NK027-2	Cyclin-depend	Pan-specific	CDK4	P11802	CDK4_HUMAN	CDK4	4612	1	3579	1	1790	1	2252	1	2680	1	2904	1	0.78	1.00	0.63	0.49	0.39	0.58	
	NN011-NN125	Pro-caspase 1	Pan-specific	CASP1ab	P29466	CASP1_HUMA	CASP1 IL1BC IL	4701	1	4724	1	2523	1	3053	1	3581	1	3897	1	1.00	1.00	0.83	0.65	0.53	0.76	
	NN033	Cytochrome C	Pan-specific	CytoC	P99999	CYC_HUMAN	CYCS CYC	3927	1	8655	1	2719	1	2748	1	3346	1	3042	1	1.00	0.45	0.35	0.32	0.31	0.39	
24	NK130-6	Protein-serine	Pan-specific	PKBb (Akt2)	P31751	AKT2_HUMAN	AKT2	4491	1	6326	1	2503	1	2480	1	3303	1	3611	1	1.00	0.71	0.57	0.39	0.40	0.52	
	NK135	Protein-serine	Pan-specific	PKCd	Q05655	KPCD_HUMAN	PRKCD	6076	1	8791	1	3065	1	3289	1	3505	1	4158	1	1.00	0.69	0.47	0.37	0.35	0.40	
	NK136	Protein-serine	Pan-specific	PKCe	Q02156	KPCE_HUMAN	PRKCE PKCE	5544	1	6909	1	3371	1	3074	1	3966	1	4090	1	1.00	0.80	0.59	0.44	0.49	0.57	
	NN006	B-cell lymphoi	Pan-specific	Bcl2	P10415	BCL2_HUMAN	BCL2	2275	1	4199	1	1863	1	2414	1	1637	1	2347	1	1.00	0.54	0.56	0.58	0.44	0.39	12
	NK200	p21-activated	Pan-specific	PAK2	Q13177	PAK2_HUMAN	PAK2	1064	1	2681	1	785	1	1005	1	717	1	2234	1	1.00	0.40	0.83	0.38	0.29	0.27	
	NN035-NN126	DNA fragment	Pan-specific	DFF35, DFF45	O00273	DFFA_HUMAN	DFFA DFF1 DF	2376	1	3511	1	1502	1	3906	1	1684	1	3711	1	0.90	0.61	0.95	1.00	0.38	0.43	
	NK140	Protein-serine	Pan-specific	PKCq	Q04759	KPCT_HUMAN	PRKCQ PRKCT	4068	1	7022	1	4746	1	4940	1	4868	1	7118	1	0.99	0.57	1.00	0.69	0.67	0.68	
16	PK060-1	Mitogen-activ	T180+Y182	p38a MAPK	Q16539	MK14_HUMAI	MAPK14 CSBP	862	3	3217	3	1692	1	2377	1	1704	1	2781	1	1.00	0.27	0.86	0.74	0.53	0.53	
25	NK196	Jun N-terminu	Pan-specific	JNK2/3	P45984	MK09_HUMAI	MAPK9 JNK2	2371	1	2772	1	1997	1	2517	1	2397	1	2231	1	1.00	0.86	0.80	0.91	0.72	0.86	
26	NK100-3	MAPK/ERK pr	Pan-specific	MEK2 (MAP2K2)	P36507	MP2K2_HUM/	MAP2K2 MEK2	949	1	3699	1	1623	1	2107	1	2273	1	2727	1	1.00	0.26	0.74	0.57	0.44	0.61	
	NK188-1	ZIP kinase (de	Pan-specific	ZIPK	O43293	DAPK3_HUM/	DAPK3 ZIPK	5205	2	9042	1	5719	1	6334	1	5624	1	6809	1	1.00	0.58	0.75	0.70	0.63	0.62	
	PN036	Histone H2A v	S139	Histone H2A.X	P16104	H2AX_HUMAI	H2AFX H2AX	2459	1	4392	1	2513	1	2447	1	2670	1	3467	1	1.00	0.56	0.79	0.56	0.57	0.61	
	NK003	Anaplastic lym	Pan-specific	ALK	Q9UM73	ALK_HUMAN	ALK	3221	1	6074	1	2960	1	2669	1	5060	1	4396	1	1.00	0.53	0.72	0.44	0.49	0.83	
	NK018-2	Calcium/calm	Pan-specific	CAMK2b	Q13554	KCC2B_HUMA	CAMK2B CAM	2960	1	5169	1	1718	2	1843	1	2854	1	4049	1	1.00	0.57	0.78	0.36	0.33	0.55	
	NK213	Mixed-lineage	Pan-specific	MLK3	Q16584	M3K11_HUM/	MAP3K11 MLK	3418	2	6591	1	3754	1	2893	1	3762	1	4502	1	1.00	0.52	0.68	0.44	0.57	0.57	13
	PN013	B-cell linker pr	Y84	BLNK	O75498	O75498_HUMAN		8886	1	9258	1	6768	2	3727	1	6788	2	9045	1	1.00	0.96	0.98	0.40	0.73	0.73	
	NK033	Cyclin-depend	Pan-specific	CDK10	Q15131	CDK10_HUMA	CDK10	4314	1	3922	1	3443	1	3070	1	3923	1	5898	1	0.66	0.73	1.00	0.52	0.58	0.67	
17	PK072-3	Protein-serine	S473	PKBa (Akt1)	P31749	AKT1_HUMAN	AKT1 PKB RAC	5584	1	5020	1	3554	1	2404	2	3585	1	5497	1	0.90	1.00	0.98	0.43	0.64	0.64	
	NN087	Proliferating c	Pan-specific	PCNA	P12004	PCNA_HUMAN	PCNA	8497	1	7433	1	6599	1	4259	1	4142	1	8457	1	0.87	1.00	1.00	0.50	0.78	0.49	
	PK044	Mitogen-activ	T222	MAPKAPK2	P49137	MAPK2_HUM	MAPKAPK2	6087	1	4287	1	3320	1	2858	1	2365	1	4968	1	0.70	1.00	0.82	0.47	0.55	0.39	
	NK145	Polo-like prote	Pan-specific	Plk1	P53350	PLK1_HUMAN	PLK1 PLK	2458	1	2210	1	1417	1	976	2	1041	1	1829	1	0.90	1.00	0.74	0.40	0.58	0.42	
	NN029	Cyclin B1	Pan-specific	Cyclin B1	P14635	CCNB1_HUM/	CCNB1 CCNB	2742	1	7010	1	2437	1	2283	1	2564	1	3036	1	1.00	0.39	0.43	0.33	0.35	0.37	
18	PN047	Jun proto-onc	S63	Jun	P05412	JUN_HUMAN	JUN	837	1	1128	2	419	2	285	1	384	1	850	1	1.00	0.74	0.75	0.25	0.37	0.34	
27	NK217	Jun N-terminu	Pan-specific	JNK1	P45983	MK08_HUMAI	MAPK8 JNK1	2130	1	4427	1	3106	1	3656	1	2524	1	2549	1	1.00	0.48	0.58	0.83	0.70	0.57	14
15	PK043	Yes-related pr	Y507	Lyn	P07948	LYN_HUMAN	LYN JTK8	1034	1	2439	1	1855	1	1495	1	1758	1	1861	1	1.00	0.42	0.76	0.61	0.76	0.72	
	NK161	Macrophage-s	Pan-specific	RONa	Q04912	RON_HUMAN	MST1R PTK8 R	3129	1	6758	1	5880	2	7560	1	3715	2	6872	1	0.89	0.41	0.91	1.00	0.78	0.49	
	PK082-2	Protein-serine	T514	PKCg	P05129	KPCG_HUMAN	PRKCG PKCG	7674	2	9974	2	15023	1	13926	1	10476	2	17553	1	0.57	0.44	1.00	0.79	0.86	0.60	

Supplementary Table 3: Differential analysis of TNBC clusters in TCGA RPPA

TCGA RPPA probe	Symbol	Gene name	Cluster E	Cluster C	Cluster A	Cluster B	Cluster D	Cluster F	85% agreement<br>Concordance
STAT5-alpha-R-V	**STAT5A**	signal transducer and activator of transcription 5A	1.00	0.04	0.09	0.10	0.41	0.42	YES
IGFBP2-R-V	IGFBP2	insulin-like growth factor binding protein 2, 36kDa	1.00	0.31	0.10	0.08	0.03	0.04	
Cyclin_D1-R-V	**CCND1**	cyclin D1	1.00	0.33	0.21	0.02	0.21	0.23	YES
TAZ_pS89-R-C	WWTR1	WW domain containing transcription regulator 1	1.00	0.34	0.07	0.00	0.01	0.07	
Syk-M-V	**SYK**	spleen tyrosine kinase	1.00	0.35	0.22	0.17	0.00	0.00	NO
AR-R-V	AR	androgen receptor	1.00	0.47	0.19	0.01	0.00	0.00	
PR-R-V	PGR	progesterone receptor	1.00	0.87	0.27	0.20	0.19	0.09	
Bcl-2-M-V	**BCL2**	B-cell CLL/lymphoma 2	1.00	0.93	0.39	0.14	0.31	0.21	YES
MSH6-R-C	MSH6	mutS homolog 6	1.00	0.56	0.15	0.16	0.33	0.37	
ATM-R-NA	ATM	ataxia telangiectasia mutated	1.00	0.60	0.22	0.27	0.62	0.55	
Ku80-R-C	XRCC5	X-ray repair complementing defective repair in Chinese hamster cells 5 (double-strand-break	1.00	0.69	0.17	0.35	0.70	0.70	
B-Raf-M-NA	**BRAF**	v-raf murine sarcoma viral oncogene homolog B	0.85	0.67	0.21	0.42	0.61	1.00	YES
beta-Catenin-R-V	**CTNNB1**	catenin (cadherin-associated protein), beta 1, 88kDa	1.00	0.71	0.23	0.45	0.77	0.89	YES
Rb-M-V	**RB1**	retinoblastoma 1	0.98	0.82	0.06	0.40	0.90	1.00	YES
GSK3-alpha-beta-M-V	**GSK3B**	glycogen synthase kinase 3 beta	1.00	0.83	0.42	0.64	0.82	0.75	YES
GSK3-alpha-beta_pS21_S9-R-V	**GSK3A**	glycogen synthase kinase 3 alpha	1.00	0.58	0.47	0.69	0.83	0.86	YES
mTOR-R-V	**MTOR**	mechanistic target of rapamycin (serine/threonine kinase)	0.88	0.73	0.38	0.59	0.89	1.00	YES
Bcl-X-R-C	**BCL2L1**	BCL2-like 1	1.00	0.80	0.31	0.68	0.94	0.96	YES
Annexin_I-R-V	ANXA1	annexin A1	0.84	0.89	1.00	0.93	0.60	0.44	
Bid-R-C	BID	BH3 interacting domain death agonist	0.92	0.80	0.33	0.72	0.93	1.00	
EGFR_pY1173-R-C	**EGFR**	epidermal growth factor receptor	0.93	0.88	0.37	0.70	0.93	1.00	YES
Src_pY527-R-V	**SRC**	v-src avian sarcoma (Schmidt-Ruppin A-2) viral oncogene homolog	1.00	0.86	0.58	0.65	0.93	0.97	YES
ERK2-R-NA	**MAPK1**	mitogen-activated protein kinase 1	1.00	0.86	0.66	0.74	0.90	0.84	YES
N-Cadherin-R-V	CDH2	cadherin 2, type 1, N-cadherin (neuronal)	0.95	0.89	0.49	0.76	0.92	1.00	
VEGFR2-R-C	**KDR**	kinase insert domain receptor (a type III receptor tyrosine kinase)	0.79	0.73	0.54	0.78	0.91	1.00	YES
eEF2K-R-V	**EEF2K**	eukaryotic elongation factor-2 kinase	0.70	0.94	1.00	0.66	0.39	0.35	YES
IGF-1R-beta-R-C	**IGF1R**	insulin-like growth factor 1 receptor	0.51	0.93	0.04	0.00	0.00	1.00	YES
Caspase-7_cleavedD198-R-C	**CASP7**	caspase 7, apoptosis-related cysteine peptidase	0.58	1.00	0.19	0.03	0.04	0.04	YES
53BP1-R-C	TP53BP1	tumor protein p53 binding protein 1	0.58	0.34	0.01	0.46	1.00	0.99	
ER-alpha-R-V	ESR1	estrogen receptor 1	0.65	0.15	0.02	0.50	0.73	1.00	
Cyclin_E1-M-V	CCNE1	cyclin E1	0.55	0.61	0.98	1.00	0.93	0.93	
E-Cadherin-R-V	CDH1	cadherin 1, type 1, E-cadherin (epithelial)	0.54	0.58	0.17	0.07	0.65	1.00	
Chk2_pT68-R-C	**CHEK2**	checkpoint kinase 2	0.51	0.55	0.84	1.00	0.90	0.68	YES
Lck-R-V	**LCK**	lymphocyte-specific protein tyrosine kinase	0.50	0.50	0.41	0.66	0.85	1.00	YES
S6_pS240_S244-R-V	RPS6	ribosomal protein S6	0.50	0.47	0.87	1.00	0.61	0.54	
p70S6K-R-V	**RPS6KB1**	ribosomal protein S6 kinase, 70kDa, polypeptide 1	0.46	0.44	0.00	0.28	1.00	0.79	
Rb_pS807_S811-R-V	**RB1**	retinoblastoma 1	0.44	0.50	1.00	0.68	0.39	0.38	YES
ACC_pS79-R-V	ACACA	acetyl-CoA carboxylase alpha	0.39	0.74	1.00	0.36	0.09	0.02	
Dvl3-R-V	DVL3	dishevelled segment polarity protein 3	0.36	0.58	1.00	0.65	0.46	0.44	
COX-2-R-C	**PTGS2**	prostaglandin-endoperoxide synthase 2 (prostaglandin G/H synthase and cyclooxygenase)	0.23	1.00	0.96	0.04	0.08	0.03	YES
NF-kB-p65_pS536-R-C	**NFKB1**	nuclear factor of kappa light polypeptide gene enhancer in B-cells 1	0.18	0.71	1.00	0.91	0.47	0.44	
PTCH-R-C	PTCH1	patched 1	0.37	0.30	1.00	0.52	0.29	0.23	
HER3-M-C	ERBB3	v-erb-b2 avian erythroblastic leukemia viral oncogene homolog 3	0.35	0.27	1.00	0.55	0.28	0.27	
S6-R-NA	RPS6	ribosomal protein S6	0.24	0.08	1.00	0.56	0.00	0.00	
Chk2-M-C	**CHEK2**	checkpoint kinase 2	0.19	0.28	1.00	0.68	0.46	0.36	NO
HSP70-R-C	**HSPA1A**	heat shock 70kDa protein 1A	0.14	0.36	1.00	0.29	0.05	0.05	NO
Collagen_VI-R-V	COL6A1	collagen, type VI, alpha 1	0.13	0.34	1.00	0.65	0.22	0.11	
Notch3-R-C	NOTCH3	notch 3	0.12	0.13	1.00	0.39	0.02	0.00	
Caveolin-1-R-V	CAV1	caveolin 1, caveolae protein, 22kDa	0.05	0.18	1.00	0.86	0.25	0.13	
Tuberin-R-C	TSC2	tuberous sclerosis 2	0.05	0.57	1.00	0.24	0.02	0.00	
Bak-R-C	BAK1	BCL2-antagonist/killer 1	0.03	0.09	1.00	0.20	0.01	0.00	
MSH2-M-C	**MSH2**	mutS homolog 2	0.01	0.12	1.00	0.57	0.27	0.21	YES
Cyclin_B1-R-V	**CCNB1**	cyclin B1	0.00	0.00	1.00	0.76	0.33	0.17	YES
MAPK_pT202_Y204-R-V	**MAPK3**	mitogen-activated protein kinase 3	0.14	0.31	0.91	1.00	0.49	0.29	NO
DJ-1-R-C	PARK7	parkinson protein 7	0.19	0.46	0.70	1.00	0.48	0.38	
P-Cadherin-R-C	CDH3	cadherin 3, type 1, P-cadherin (placental)	0.31	0.36	0.68	0.93	0.91	1.00	
INPP4B-G-C	INPP4B	inositol polyphosphate-4-phosphatase, type II, 105kDa	0.16	0.20	0.43	0.84	0.83	1.00	
HER2-M-V	**ERBB2**	v-erb-b2 avian erythroblastic leukemia viral oncogene homolog 2	0.19	0.45	0.00	0.10	1.00	0.28	YES
ACC1-R-C	ACACB	acetyl-CoA carboxylase beta	0.01	0.04	0.18	0.05	1.00	0.29	
c-Kit-R-V	KIT	v-kit Hardy-Zuckerman 4 feline sarcoma viral oncogene homolog	0.26	0.42	0.23	0.44	0.99	1.00	
GATA3-M-V	GATA3	GATA binding protein 3	0.23	0.01	0.01	0.58	0.80	1.00	
Caspase-9_cleavedD330-R-C	CASP9	caspase 9, apoptosis-related cysteine peptidase	0.28	0.04	0.65	0.13	0.64	1.00	
ER-alpha_pS118-R-V	ESR1	estrogen receptor 1	0.00	0.00	0.01	0.11	0.53	1.00	
GAB2-R-V	GAB2	GRB2-associated binding protein 2	0.19	0.46	0.13	0.70	0.35	1.00	
Claudin-7-R-V	CLDN7	claudin 7	0.10	0.37	0.29	0.01	0.21	1.00	

Supplementary Table 4: mRNA expression of upregulated proteins in TNBC in TCGA and METABRIC datasets and Neve Cell Line dataset

TCGA Breast

Gene Symt	Reporter II	t-Test	P-value	Q-value	Fold Change
**EGFR**	A_23_P215	12.00189	2.77E-18	7.68E-16	4.8
**CRYAB**	A_24_P206	10.70911	8.45E-17	1.68E-14	3.0
**SOD2**	AK093984_	7.83787	6.49E-11	3.16E-09	2.4
**CAMK1D**	A_23_P124	4.624306	1.09E-05	1.37E-04	1.8
**CDKL1**	A_23_P997	6.396247	1.63E-08	4.25E-07	1.7
**FAS**	NM_00004	4.448376	1.91E-05	2.25E-04	1.6
**ROCK2**	A_23_P209	8.933776	1.80E-13	1.66E-11	1.6
**CDK1**	A_23_P138	5.098631	1.43E-06	2.26E-05	1.6
**PRKDC**	A_23_P330	5.220744	1.01E-06	1.66E-05	1.5
SRC	NM_00541	5.534254	2.71E-07	5.14E-06	1.5
CALR	A_23_P672	5.625353	2.83E-07	5.35E-06	1.4
BIRC2	A_23_P869	5.817717	1.44E-07	2.94E-06	1.4
PDIA4	A_23_P428	4.129636	5.65E-05	5.88E-04	1.4
CSK	A_23_P152	5.665267	2.13E-07	4.17E-06	1.3
KSR1	A_32_P660	7.785325	5.43E-11	2.71E-09	1.3
PAK2	A_23_P106	3.22253	1.05E-03	7.70E-03	1.3
MAPK14	A_23_P214	4.41383	2.20E-05	2.54E-04	1.3
HSP90B1	A_24_P150	4.132559	5.45E-05	5.69E-04	1.3
CTNNB1	A_24_P608	2.716441	4.39E-03	2.67E-02	1.3
MAPK7	A_23_P100	4.593475	1.07E-05	1.35E-04	1.2
RPS6KB2	A_23_P243	2.284334	1.25E-02	6.57E-02	1.2
CSNK1D	A_23_P207	2.903189	2.57E-03	1.68E-02	1.2
HSPA8	A_32_P643	2.745768	3.89E-03	2.41E-02	1.2
HSPH1	A_23_P881	2.282669	1.24E-02	6.51E-02	1.2
NFE2L2	A_23_P576	1.843702	3.50E-02	1.56E-01	1.1
STAT1	A_24_P274	0.978762	1.66E-01	5.41E-01	1.1
TPTE2	NM_13078	0.772255	2.22E-01	6.71E-01	1.1
SET	A_24_P739	0.985814	1.64E-01	5.38E-01	1.1
HSPA5	A_23_P832	1.04744	1.50E-01	5.00E-01	1.1
CDK11A	A_24_P967	1.10023	1.38E-01	4.69E-01	1.1
MAP2K2	A_23_P208	0.809898	2.10E-01	6.46E-01	1.0
CDK11B	A_23_P501	0.916222	1.82E-01	5.79E-01	1.0
HSP90AA1	A_32_P142	0.565447	2.87E-01	8.09E-01	1.0
MAP2K1	A_23_P202	0.666061	2.54E-01	7.41E-01	1.0
ST13	A_32_P156	0.510364	3.06E-01	8.46E-01	1.0
HMOX1	A_23_P120	0.198017	4.22E-01	1.05E+00	1.0
CAMK4	A_23_P250	0.166698	4.34E-01	1.07E+00	1.0
DNAJB1	A_23_P900	-1.61767	9.45E-01	1.60E+00	-1.1
HMOX2	A_24_P381	-2.19281	9.84E-01	1.55E+00	-1.1
ANP32A	A_32_P186	-1.76518	9.59E-01	1.59E+00	-1.1
STAT6	A_23_P478	-1.26915	8.95E-01	1.60E+00	-1.1
PRKCH	A_23_P205	-1.42468	9.20E-01	1.60E+00	-1.1
CANX	A_23_P147	-2.87872	9.97E-01	1.49E+00	-1.2
MAPK10	A_23_P450	-1.2469	8.91E-01	1.60E+00	-1.2
DUSP1	A_32_P171	-3.84502	1.00E+00	1.41E+00	-1.3
PPP2R5A	A_24_P251	-3.52468	1.00E+00	1.43E+00	-1.4
IKBKB	A_23_P216	-5.81172	1.00E+00	1.31E+00	-1.4
PRKCD	A_23_P144	-7.5467	1.00E+00	1.27E+00	-1.5
STRN3	A_23_P392	-5.38178	1.00E+00	1.33E+00	-1.6

Curtis Breast

Gene Symt	Reporter II	t-Test	P-value	Q-value	Fold Change
**EGFR**	ILMN_1798	14.64081	4.53E-35	5.73E-33	2.4
**CRYAB**	ILMN_1729	18.82972	7.56E-50	4.79E-47	4.7
**SOD2**	ILMN_2336	18.00456	9.14E-48	4.85E-45	2.6
CAMK1D	ILMN_1795	7.558551	4.16E-13	7.52E-12	1.2
CDKL1	ILMN_1724	4.237329	1.58E-05	1.29E-04	1.0
FAS	ILMN_1808	7.384478	1.16E-12	2.01E-11	1.1
ROCK2	ILMN_1659	8.590094	4.08E-16	9.69E-15	1.2
**CDK1**	ILMN_1747	12.6753	5.71E-30	4.41E-28	1.6
PRKDC	ILMN_1769	9.579215	4.64E-19	1.45E-17	1.4
SRC	ILMN_1729	5.499005	4.45E-08	4.85E-07	1.2
CALR	ILMN_1736	11.70114	1.97E-26	1.15E-24	1.2
BIRC2	ILMN_2182	8.761066	1.54E-16	3.79E-15	1.3
PDIA4	ILMN_1815	3.329202	4.97E-04	3.33E-03	1.1
CSK	ILMN_1754	6.939414	1.36E-11	2.11E-10	1.2
KSR1	ILMN_1754	3.256944	6.33E-04	4.15E-03	1.0
PAK2	ILMN_1712	-0.66234	7.46E-01	1.18E+00	-1.0
MAPK14	ILMN_1788	3.493121	2.77E-04	1.93E-03	1.1
HSP90B1	ILMN_2096	7.237332	2.28E-12	3.83E-11	1.2
CTNNB1	ILMN_1757	2.554543	5.61E-03	3.04E-02	1.1
MAPK7	ILMN_1709	4.887382	8.47E-07	8.01E-06	1.1
RPS6KB2	ILMN_2364	5.089147	3.08E-07	3.05E-06	1.1
CSNK1D	ILMN_1720	4.894726	8.53E-07	8.06E-06	1.1
HSPA8	ILMN_2413	3.488862	2.80E-04	1.95E-03	1.1
HSPH1	ILMN_1712	6.164856	1.17E-09	1.51E-08	1.2
NFE2L2	ILMN_1790	1.693771	4.57E-02	1.87E-01	1.0
STAT1	ILMN_1777	9.280415	2.07E-18	6.10E-17	1.4
TPTE2	ILMN_1785	3.287028	5.83E-04	3.85E-03	1.1
SET	ILMN_1742	0.896468	1.85E-01	5.44E-01	1.0
HSPA5	ILMN_1773	-8.0016	1.00E+00	1.06E+00	-1.2
CDK11A	ILMN_1711	0.042375	4.83E-01	9.77E-01	1.0
MAP2K2	ILMN_1657	2.791662	2.81E-03	1.64E-02	1.1
CDK11B	ILMN_1765	-0.40609	6.57E-01	1.13E+00	-1.0
HSP90AA1	ILMN_1687	10.06963	1.74E-20	6.13E-19	1.3
MAP2K1	ILMN_1694	6.707613	5.45E-11	7.95E-10	1.2
ST13	ILMN_1765	-2.98944	9.98E-01	1.18E+00	-1.1
HMOX1	ILMN_1800	2.806536	2.67E-03	1.56E-02	1.1
CAMK4	ILMN_1767	1.076974	1.41E-01	4.49E-01	1.0
DNAJB1	ILMN_1775	0.113722	4.55E-01	9.45E-01	1.0
HMOX2	ILMN_1658	-1.12478	8.69E-01	1.23E+00	-1.0
ANP32A	ILMN_1807	-1.70018	9.55E-01	1.23E+00	-1.0
STAT6	ILMN_1763	-3.19461	9.99E-01	1.17E+00	-1.1
PRKCH	ILMN_1780	-0.01506	5.06E-01	1.00E+00	-1.0
CANX	ILMN_1762	0.237514	4.06E-01	8.89E-01	1.0
MAPK10	ILMN_1766	6.240078	9.50E-10	1.24E-08	1.1
DUSP1	ILMN_1781	-4.29241	1.00E+00	1.13E+00	-1.3
PPP2R5A	ILMN_1738	-8.91924	1.00E+00	1.04E+00	-1.3
IKBKB	ILMN_1727	-15.3896	1.00E+00	1.04E+00	-1.5
PRKCD	ILMN_1682	-1.69981	9.55E-01	1.23E+00	-1.0
STRN3	ILMN_1772	-10.3357	1.00E+00	1.05E+00	-1.3

Neve CellLine

Gene Symt	Reporter II	t-Test	P-value	Q-value	Fold Change
**EGFR**	201983_s_	4.660931	1.51E-05	2.81E-03	4.8
**CRYAB**	209283_at	1.860948	3.58E-02	2.60E-01	1.9
**SOD2**	215223_s_	3.815774	2.92E-04	1.52E-02	2.6
CAMK1D	220246_at	0.922518	1.81E-01	6.64E-01	1.1
CDKL1	207766_at	1.806259	3.89E-02	2.74E-01	1.1
FAS	204780_s_	1.855897	3.57E-02	2.60E-01	1.3
ROCK2	202762_at	3.561935	4.53E-04	2.05E-02	1.4
CDK1	203214_x_	2.839291	3.46E-03	6.70E-02	1.4
**PRKDC**	208694_at	3.47297	5.85E-04	2.38E-02	1.7
SRC	221281_at	-0.60106	7.24E-01	1.18E+00	-1.0
CALR	214316_x_	3.047335	1.98E-03	4.94E-02	1.3
**BIRC2**	202076_at	4.290859	4.95E-05	5.25E-03	1.8
PDIA4	211048_s_	-0.4364	6.68E-01	1.16E+00	-1.0
CSK	202329_at	-2.52594	9.92E-01	1.07E+00	-1.2
KSR1	213770_at	-0.1459	5.58E-01	1.12E+00	-1.0
PAK2	205962_at	1.767786	4.20E-02	2.87E-01	1.0
MAPK14	202530_at	1.482769	7.29E-02	3.94E-01	1.1
HSP90B1	216449_x_	1.021819	1.57E-01	6.12E-01	1.1
CTNNB1	201533_at	3.071932	1.83E-03	4.67E-02	1.4
MAPK7	207292_s_	3.097907	1.87E-03	4.74E-02	1.2
RPS6KB2	203777_s_	-4.31088	1.00E+00	1.01E+00	-1.5
CSNK1D	207945_s_	0.25323	4.01E-01	9.84E-01	1.0
HSPA8	208687_x_	0.363632	3.59E-01	9.40E-01	1.0
HSPH1	206976_s_	2.262533	1.46E-02	1.53E-01	1.4
NFE2L2	201146_at	2.683906	5.11E-03	8.30E-02	1.4
STAT1	200887_s_	1.472649	7.44E-02	3.98E-01	1.3
TPTE2	-				
SET	210231_x_	2.473923	8.74E-03	1.14E-01	1.2
HSPA5	211936_at	1.882874	3.32E-02	2.50E-01	1.2
CDK11A	210474_s_	1.530709	6.73E-02	3.76E-01	1.1
MAP2K2	202424_at	3.36337	9.05E-04	3.03E-02	1.4
CDK11B	212401_s_	0.893579	1.89E-01	6.80E-01	1.1
HSP90AA1	210211_s_	0.303261	3.82E-01	9.67E-01	1.0
MAP2K1	202670_at	0.459949	3.24E-01	8.98E-01	1.1
ST13	207040_s_	1.764866	4.25E-02	2.89E-01	1.2
**HMOX1**	203665_at	2.754728	5.32E-03	8.52E-02	1.6
CAMK4	210349_at	1.370251	8.95E-02	4.44E-01	1.1
DNAJB1	200666_s_	0.689809	2.47E-01	7.86E-01	1.1
HMOX2	218120_s_	-1.96613	9.71E-01	1.12E+00	-1.3
ANP32A	201043_s_	0.076524	4.70E-01	1.05E+00	1.0
STAT6	201331_s_	0.568134	2.86E-01	8.46E-01	1.1
PRKCH	206099_at	-2.20916	9.83E-01	1.10E+00	-1.2
CANX	208853_s_	0.542612	2.95E-01	8.57E-01	1.1
MAPK10	204813_at	-2.25334	9.85E-01	1.09E+00	-1.2
DUSP1	201044_x_	0.786621	2.18E-01	7.34E-01	1.1
PPP2R5A	202186_x_	-0.11075	5.44E-01	1.11E+00	-1.0
IKBKB	209341_s_	-0.79604	7.85E-01	1.19E+00	-1.1
PRKCD	202545_at	-2.47635	9.91E-01	1.07E+00	-1.4
STRN3	215505_s_	-0.58512	7.19E-01	1.18E+00	-1.0
